# From telomere dysfunction to seno-antigen selection: a conceptual framework and route-specific evidence appraisal for senolytic immunotherapy

**DOI:** 10.3389/fsysb.2026.1940319

**Published:** 2026-10-27

**Authors:** Sarfaraz K. Niazi

**Affiliations:** College of Pharmacy and Pharmaceutical Sciences, Washington State University, Spokane, DC, United States

**Keywords:** cellular senescence, combinatorial targeting, conceptual framework, evidence appraisal, immune senolysis, immunopeptidomics, seno-antigen, telomere dysfunction

## Abstract

Cellular senescence causally contributes to age-related and chronic diseases, rendering the targeted immune clearance of pathological senescent cells a promising therapeutic avenue. Progress remains limited by heterogeneity across cell lineages, tissues, inducing stressors, and temporal factors. As antigen expression alone does not ensure immune accessibility, functional recognition, or safety for healthy tissues, these aspects warrant thorough evaluation. A conceptual framework is proposed, distinct from a validated or implementable algorithm, for identifying and prioritizing senescence-associated antigens. This framework requires four criteria that a single candidate antigen must meet within the same cellular context: persistent pathological senescence, immune accessibility via a confirmed surface antigen or naturally presented human leukocyte antigen (HLA) peptide, modality-appropriate functional immune recognition, and exclusion from vital healthy or beneficially senescent states. The mechanistic origin, such as telomere dysfunction, serves as an optional stratification axis that defines coherent subsets rather than a universal prerequisite; data lacking matched telomere and antigen information in the same cells remain unverified. The process distinguishes target nomination from product validation: evidence of recognition in a single modality, such as chimeric antigen receptor (CAR) targeting of a surface antigen, does not confirm validation for alternatives like peptide vaccines. Application of the framework to urokinase-type plasminogen activator receptor (uPAR) and glycoprotein nonmetastatic melanoma protein B (GPNMB) indicates that neither candidate currently meets all criteria, and the telomere axis remains untested for both. Given that multivalent vaccination elicits immunity against each component independently, combining candidates that fail individual criteria risks amplifying liabilities rather than ensuring intersectional specificity. This outcome would require an engineered AND-gated recognition system, where the capitalized operators AND, OR, and NOT denote Boolean logic gates rather than emphasis. The framework remains in the validation phase. Each criterion receives an ordinal grade, a non-compensatory decision rule, a route-specific ligand-density window that sets the quantitative condition for recognition and healthy-cell sparing, and a predefined seven-compartment counter-screening panel. Recognition architectures are formally compared, and a pre-registered benchmarking study with specified falsification criteria is outlined as the framework’s validation test.

## 1 Introduction

### 1.1 Cellular senescence as a context-dependent therapeutic target

Cellular senescence constitutes a stress-associated condition distinguished by a persistent restriction of proliferative capacity and diverse alterations in DNA-damage signaling, chromatin organization, lysosomal and metabolic functions, morphology, and secretory activity. This definition is intentionally broader than that of irreversible cell-cycle arrest, as such arrest can also occur during quiescence, differentiation, and transient checkpoint activation, whereas some postmitotic cells develop senescence-like features and inflammatory characteristics without providing informative proliferation indicators. Consequently, the prevailing consensus regards senescence as a multidimensional phenotype that necessitates multiple orthogonal measurements, interpreted within the context of the relevant lineage and tissue (Gorgoulis et al., 2019; Ogrodnik et al., 2024; Suryadevara et al., 2024).

Senescence is not inherently pathological. Programmed senescence supports embryonic patterning, and transient populations of senescent fibroblasts and endothelial cells facilitate cutaneous wound healing through paracrine signaling that supports appropriate tissue remodeling. These findings demonstrate that senescence can function as an adaptive, temporally constrained process when induction, activity, and clearance are carefully coordinated with developmental or reparative stages. Consequently, therapeutic strategies must differentiate between persistent detrimental cell populations and beneficial transient states (Munoz-Espin et al., 2013; Storer et al., 2013; Demaria et al., 2014).

Pathology becomes prominent when senescent cells persist, accumulate, or continuously generate secondary damage through inflammatory cytokines, proteases, extracellular vesicle cargo, matrix remodeling, or paracrine propagation. Genetic elimination of selected p16-associated populations delayed several age-associated disorders in progeroid mice and attenuated organ deterioration in naturally aged mice. Pharmacological studies demonstrated that senescent cells impair physical function, whereas senolytic treatments enhance specific outcomes. These investigations provide causal evidence supporting cell clearance; however, they apply to specific experimental populations and do not establish that all p16-associated or nonproliferating cells should be eliminated (Baker et al., 2011; 2016; Xu et al., 2018; Zhu et al., 2015).

Two distinct inquiries must therefore be distinguished. The first concerns whether senescent cells causally contribute to disease; the evidence summarized above affirms that they do, and this question is no longer the limiting factor. The second asks whether any specific molecular target can distinguish the senescent cells that drive pathology from the senescent and non-senescent cells necessary for healthy physiology. Only the second question remains unresolved, and it is pivotal in determining whether immune senolysis can be conducted safely. The primary therapeutic challenge, therefore, is achieving selectivity, that is, the target’s discriminative capacity, rather than further evidence that senescence is consequential. Senescent phenotypes vary by lineage, tissue type, initiating stress, lesion severity, temporal factors, age, sex, disease state, and immune microenvironment. Cell-type-specific transcriptomic signatures, comprehensive multi-omics profiling across controlled human senescence models, and recent research on human immunity collectively show that the composition and immunogenicity of senescent cells differ by lineage and inducer. This evidence challenges the concept of a universal marker-based target and supports an intersectional definition of a pathological senotype (Sanborn et al., 2025; Anerillas et al., 2026; Goyer et al., 2026).

### 1.2 Telomere dysfunction as a mechanistic anchor

Telomeres constitute the terminal structures of chromosomes, characterized by tandem repeats, a single-stranded overhang, higher-order architecture, and shelterin proteins that safeguard natural termini from being interpreted as DNA breaks. Progressive erosion may lead to critically short chromosome ends; however, telomeres can also become dysfunctional through mechanisms such as uncapping, disruption of shelterin components, oxidative base damage, replication fork failure, transcriptional stress, or telomeric R-loops, even without a significant reduction in average length. Dysfunctional telomeres trigger ataxia-telangiectasia mutated (ATM)- or ATM- and Rad3-related (ATR)-associated deoxyribonucleic acid (DNA) damage response pathways and promote stabilization of p53-p21 and p16-retinoblastoma protein (RB)-mediated senescence programs (Takai et al., 2003; Hewitt et al., 2012; Rossiello et al., 2022).

The distinction between telomere length and telomere function is fundamentally important. A mean measurement across a population may obscure the existence of one or more critically short telomeres, while adequate repeat length does not preclude oxidative damage, structural deprotection, or persistent telomeric signaling. Targeted induction of telomeric 8-oxoguanine has been shown to induce fragility and premature senescence without the conventional process of erosion. Furthermore, manipulating damage-associated telomeric RNA has reduced telomeric DNA damage signaling, senescence, and inflammation in murine models, while also enhancing the properties of hematopoietic cells derived from older human donors. These findings support the perspective that telomeric damage signaling constitutes an active biological process rather than merely a passive biomarker of aging (Barnes et al., 2022; Oppezzo et al., 2026).

Telomere dysfunction is not an omnipresent cause of senescence. Factors such as oncogenic signaling, mitochondrial damage, proteotoxic stress, chemotherapy, irradiation, and inflammation can provoke persistent senescence without substantial evidence that telomeres are the initial lesion. Telomere dysfunction matters not as a replacement for a multidimensional definition but as a mechanistic pathway to distinguish a clinically relevant subclass from chronological aging, generalized DNA damage, and unrelated senescent states. This delineation enhances the biological criteria for antigen discovery (Rossiello et al., 2022; Ogrodnik et al., 2024).

### 1.3 Immune senolysis and the seno-antigen problem

The immune system primarily identifies and removes damaged or senescent cells through natural killer cells, macrophages, and CD4 and CD8 T lymphocytes. Studies show that compromised perforin-dependent cytotoxic surveillance increases senescent cell accumulation and accelerates age-related abnormalities in murine models, supporting the hypothesis that insufficient clearance contributes to these processes. Senescent cells evade immune surveillance through various mechanisms, including the loss or shedding of activating ligands, the expression of inhibitory human leukocyte antigen (HLA) or checkpoint molecules, the presence of suppressive glycans, and immune decline associated with aging (Ovadya et al., 2018; Pereira et al., 2019; Munoz et al., 2019).

Therapeutic immune senolysis aims to strengthen, redirect, or substitute immune surveillance through various methods such as vaccination, engineered cells, antibodies, or checkpoint modulation. These modalities provide capabilities that small molecules do not possess: antibodies can recruit complement and fragment crystallizable (Fc)-dependent effectors; conventional T cells recognize intracellular source proteins presented via HLA-bound peptides; engineered receptors can identify intact surface structures independently of peptide-HLA specificity; and memory lymphocytes or persistent cellular products can sustain surveillance even after the administered material has been eliminated. Nevertheless, these features also elevate the risk of adverse outcomes if the target is misidentified, as a self-reactive response may endure, expand upon antigen re-exposure, or harm healthy cells during episodes of infection, repair, surgery, or aging (Amor et al., 2020; Amor et al., 2024; Demaria et al., 2014).

The term “seno-antigen” is used here as an operational therapeutic designation rather than a universal biological category. A seno-antigen is a molecular structure associated with a defined pathological senescent population that an immune effector can recognize: an intact extracellular domain, glycan, or modified epitope accessible to antibodies and engineered cells, or a naturally processed peptide displayed by an HLA molecule and accessible to a T-cell receptor. Association with senescence is necessary but insufficient, because immune accessibility and functional recognition are separate requirements (Wollscheid et al., 2009; Purcell et al., 2019; Amor et al., 2020).

HLA immunopeptidomics provides direct evidence for the T-cell pathway, as a recovered peptide has undergone processing, transport, HLA loading, and surface presentation. However, presentation does not equate to immunogenicity. A self-peptide displayed may be subject to tolerance, may lack a responsive receptor, may not reach the density necessary for cytotoxic response, or may also be presented by essential healthy cells. Recent studies on human senescent cells indicate that an altered HLA-associated repertoire does not consistently elicit recognition by CD8 T cells or natural killer cells, and that immunogenicity depends heavily on cell type and inducer (Purcell et al., 2019; Goyer et al., 2026). The challenge of surface antigen identification is equally complex, as a candidate transcript may encode an intracellular isoform, a shielded domain, a rapidly internalized protein, or a shed decoy, and may remain accessible on activated leukocytes, endothelium, progenitor cells, or wound-associated stroma (Wollscheid et al., 2009; Suryadevara et al., 2024).

### 1.4 Why is integrative bioinformatics required

Antigen selection encompasses various biological scales that are seldom measured concurrently. Bulk transcriptomics provides robust comparative analyses among groups but complicates interpretation due to variations in cellular composition and states; single-cell techniques elucidate rare cell populations yet are affected by dropout events, ambient RNA contamination, and pseudoreplication; spatial methodologies identify cellular niches and immune proximity; total proteomics validates protein expression; surfaceomics examines extracellular exposure of molecular structures; immunopeptidomics quantifies displayed ligands; and immune assays determine whether accessible structures are involved in immune recognition. No single modality supersedes the others, as each addresses distinct investigative questions. The most informative discovery datasets integrate multiple modalities from the same donors and, ideally, the same cells (Purcell et al., 2019; Sanborn et al., 2025; Suryadevara et al., 2024).

The ranking process should exclude healthy states rather than appending them afterward. A valuable negative reference set comprises proliferating, quiescent, terminally differentiated, interferon-exposed, and activated cells, along with progenitors, healthy aging processes, wound-related senescence, and developmental stages. The multivalent design adds another layer, as the optimal panel is not simply the set of individually highest-ranked candidates; components may offer redundant coverage, share a common regulatory pathway and thus fail collectively, compete during processing, or pose cumulative risks to healthy tissue. Mathematical frameworks for vaccine design demonstrate that panel size, allele frequency, population coverage, redundancy, and probabilistic presentation can be systematically represented as explicit objectives and constraints (Toussaint et al., 2008; Liu et al., 2020; Dai and Gifford, 2023).

The failure modes of a naive discovery pipeline are instructive because they recur across the literature. Selecting the most upregulated transcripts in a senescent-versus-proliferating contrast conflates state with composition, rewards abundant but inaccessible intracellular proteins, ignores whether a protein reaches the surface or is naturally presented, and applies no penalty for expression on indispensable cells. Layering an epitope predictor onto such a list compounds the problem, because prediction inherits the training-data bias of existing immunopeptidomes and cannot distinguish a presented self-peptide that is tolerated from one that is functionally recognized. An integrative framework is therefore not merely a matter of using more data; it is a matter of ordering the evidence so that hard requirements gate soft ones, so that correlated data are not counted as independent confirmation, and so that the decision objective is selectivity under physiological challenge rather than differential abundance (Wollscheid et al., 2009; Purcell et al., 2019; Atkins et al., 2024).

### 1.5 Scope, thesis, and contributions

This review synthesizes telomere biology, senescence heterogeneity, immune surveillance, existing immune-senolytic interventions, single-cell and spatial bioinformatics, proteomics, surfaceomics, HLA immunopeptidomics, epitope prediction, population coverage, and the conceptual framework for candidate prioritization. The emphasis is not on the general assertion that senescent cells contribute to disease but on the specific challenge of defining and evaluating antigens associated with a persistently, mechanistically characterized pathological senescent population. Telomere dysfunction is considered one possible mechanistic layer that a target product profile may select, alongside oncogenic, mitochondrial, inflammatory, therapy-induced, and replicative pathways, rather than as a mandatory criterion of the review (Rossiello et al., 2022; Ogrodnik et al., 2024; Purcell et al., 2019).

The central thesis of this review, summarized in Figure 1, is that a universal senescence antigen is unlikely to provide the specificity and coverage required for safe immune clearance. Instead, a defensible candidate must meet four essential criteria within the same pathological cells: persistent pathological senescence; experimentally verified surface or HLA accessibility; functional immune recognition in the intended modality; and stringent exclusion from indispensable healthy and beneficially senescent states. The mechanistic origin, including telomere dysfunction, constitutes an optional stratification axis that can delineate a coherent subset but is not a universal requirement. It is recorded as untested in the absence of matched telomere and antigen data within the same cells. What is presented here is explicitly a proposed conceptual decision architecture rather than an implemented, validated, or self-optimizing algorithm. No code, executable workflow, objective function, dataset, or prospective benchmark is claimed herein. The construction and testing of such an implementation are acknowledged as necessary future endeavors (Sanborn et al., 2025; Goyer et al., 2026; Dai and Gifford, 2023).

**FIGURE 1:**
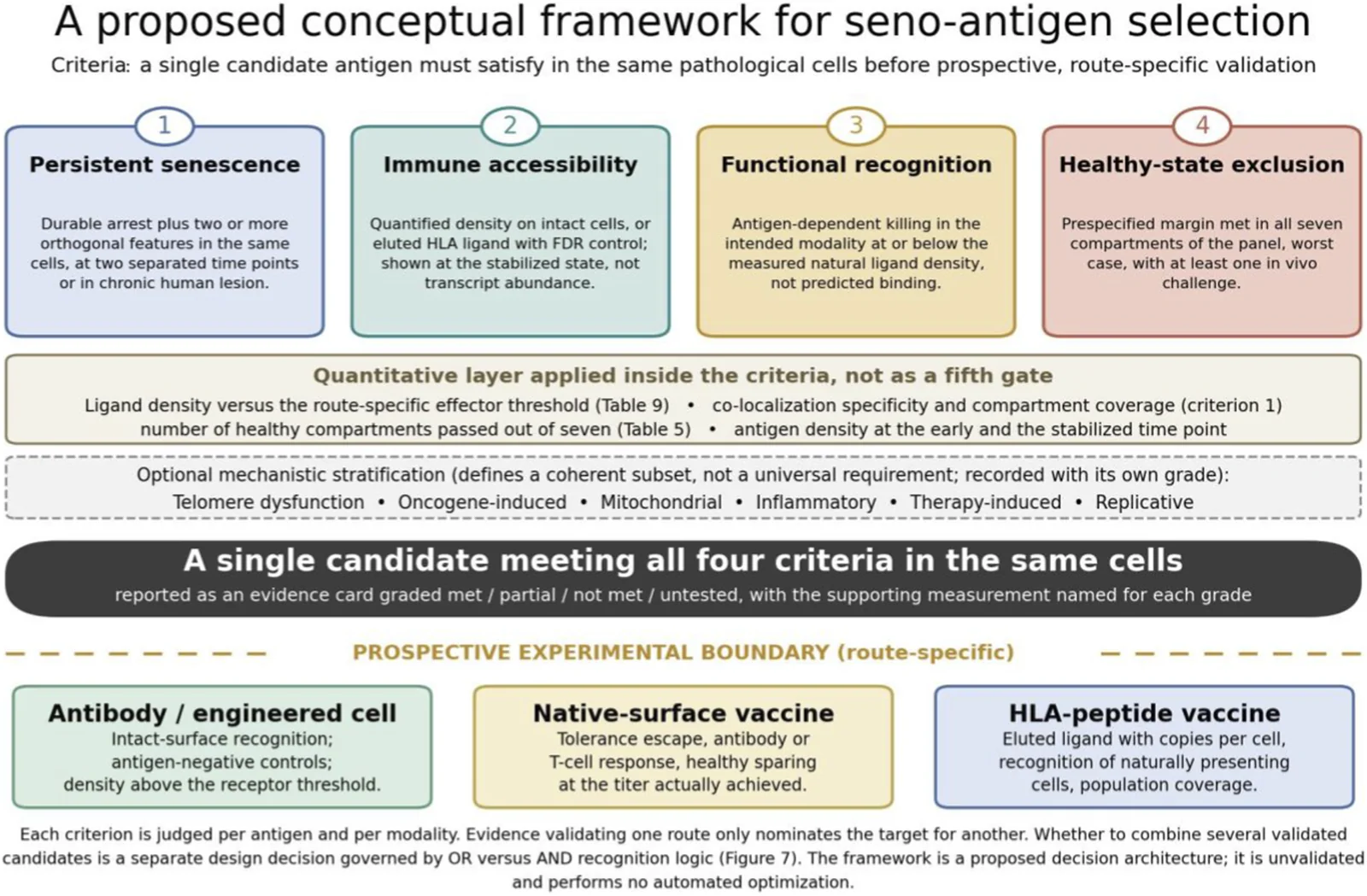
A proposed conceptual framework for seno-antigen selection. Four essential criteria must be satisfied by a single candidate antigen within the same pathological cells, with a mechanistic origin (including telomere dysfunction) serving as an optional stratification axis documented with its own grade. Each criterion is assessed per antigen and per modality; therefore, evidence supporting only one pathway nominates the target for consideration in another. Below the boundary, prospective validation is specific to the pathway. The decision to combine multiple validated candidates is a separate choice governed by OR-versus-AND recognition logic. This framework represents a proposed, unvalidated decision architecture and does not perform automated optimization. The figure also records the quantitative layer that operates inside the criteria rather than as a fifth gate: the route-specific ligand-density window, the co-localization specificity and coverage thresholds for criterion one, the number of healthy compartments passed out of the seven in the prespecified counter-screening panel, and antigen density at the early and the stabilized time point. Abbreviations: AND, Boolean conjunction operator denoting a recognition rule satisfied only when both specified inputs are present on the same cell; FDR, false discovery rate; HLA, human leukocyte antigen; OR, Boolean disjunction operator denoting a rule satisfied when any single component is recognized. AND and OR are capitalized because they name Boolean logic operations and not for emphasis.

A second thesis concerns combination. The decision to target multiple validated candidates simultaneously constitutes a design choice distinct from candidate selection and must conform to the recognition logic inherent to the selected modality. Throughout this review, the capitalized terms AND, OR, and NOT denote Boolean logic operations that define a recognition rule, and the capitalization carries that technical meaning rather than any rhetorical emphasis; an AND-gated receptor engages only when both specified inputs are present on the same cell, an OR architecture engages when any one component is recognized, and a NOT gate vetoes engagement when a healthy-restricted antigen is present. Conventional multivalent vaccination operates on OR logic: it enhances immunity against each component independently and does not require any single cell to express all components. Consequently, combining candidates that fail different criteria yields the union of their liabilities and may increase toxicity to healthy tissues rather than achieving specificity. Intersectional specificity, in which activity requires the concurrent presence of two or more antigens on the same cell, can be achieved only through an engineered AND-gated recognition system, such as a logic-gated chimeric antigen receptor or a synthetic Notch circuit, rather than through traditional vaccination. This distinction, clarified in the practical example and formalized in Figure 2, redefines multivalency as a tool for coverage and escape-resistance among individually qualified antigens, rather than for attaining selectivity (Amor et al., 2020; Yang et al., 2023; Dai and Gifford, 2023).

**FIGURE 2:**
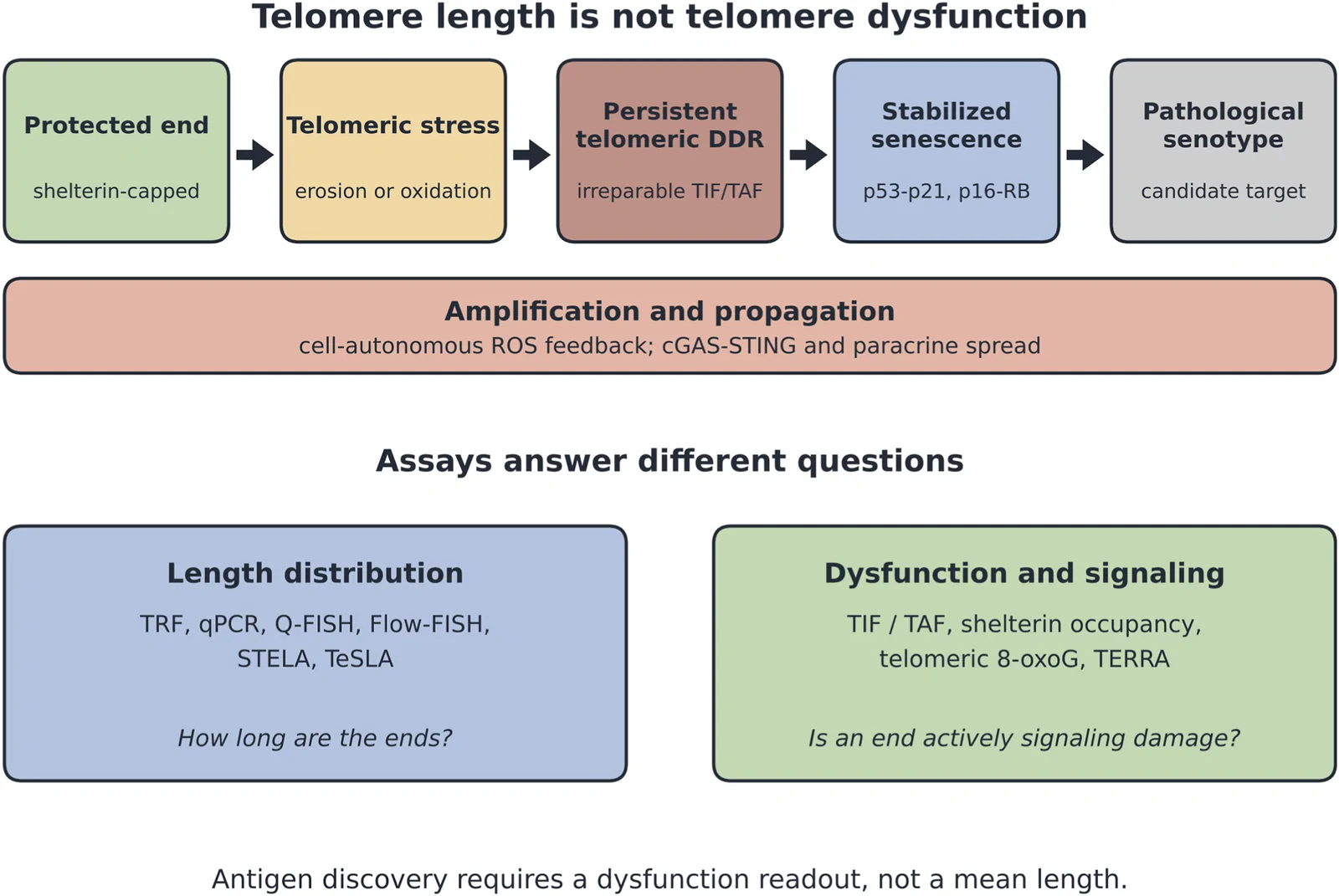
Telomere length does not necessarily indicate telomere dysfunction. A mechanistic continuum extends from protected chromosome termini through telomeric stress, characterized by a persistent telomeric DNA damage response, to stabilized senescence and culminates in a pathological senotype, involving both cell-autonomous and paracrine amplification. Amplification is cell-autonomous, through mitochondrial ROS that renew nuclear and telomeric damage, and paracrine, through cytosolic telomeric DNA sensed by cGAS-STING, ATM-c-ABL-YAP1 signaling to IL-18, and extracellular vesicles carrying telomeric DNA to neighboring cells. Representative assays are named in the panel and compared in detail in Table 4. Length-distribution assays and dysfunction or signaling assays address distinct questions and should not be conflated under a single designation. Abbreviations: 8-oxoG, 8-oxoguanine; ATM, ataxia-telangiectasia mutated; cGAS, cyclic GMP-AMP synthase; c-ABL, cellular Abelson tyrosine-protein kinase; DDR, DNA-damage response; DNA, deoxyribonucleic acid; Flow-FISH, flow cytometry combined with fluorescence *in situ* hybridization; IL-18, interleukin-18; p16-RB, p16-retinoblastoma protein; qPCR, quantitative polymerase chain reaction; Q-FISH, quantitative fluorescence *in situ* hybridization; ROS, reactive oxygen species; STELA, single telomere length analysis; STING, stimulator of interferon genes; TAF, telomere-associated DNA damage focus; TERRA, telomeric repeat-containing RNA; TeSLA, telomere shortest length assay; TIF, telomere dysfunction-induced focus; TRF, terminal restriction fragment; YAP1, Yes-associated protein 1.

The contributions of this review are therefore fourfold: four compulsory, route-aware selection criteria with explicit grading including an untested category; a conceptual two-route decision architecture that separates surface and HLA evidence and separates target nomination from product validation; a worked example that applies the criteria to two published candidates and concludes that neither currently passes; and an evidence-gated development sequence, free of calendar forecasts, in which reversible modalities precede durable self-directed immunity. The framework is offered as a method for deciding whether a candidate warrants progression, not as a validated pipeline or an antigen list.

## 2 Evidence synthesis and scope

This article is a single-author, structured-narrative review with a conceptual framework component and is not a systematic review; it makes no claim to systematic-review completeness, and one investigator selected and interpreted the evidence, a limitation stated plainly here. The objective is to reconcile heterogeneous experimental traditions and articulate a decision framework rather than to estimate a single intervention effect, for which a narrative synthesis is appropriate. Narrative flexibility creates risks of selective retrieval and overrepresentation of confirmatory evidence, which are reduced but not eliminated by documenting the review questions, conceptual boundaries, information sources, eligibility logic, and the treatment of contradictory findings, and by applying the SANRA quality criteria as a drafting self-audit. Because searching, screening, extraction, interpretation, and candidate grading were all performed by a single person, no second-reviewer adjudication of inclusion or grading was available, and independent verification is recommended before the framework informs any clinical decision (Grant and Booth, 2009; Ferrari, 2015; Snyder, 2019; Baethge et al., 2019).

Six measures constrained that process, and each is described here, including what it does and does not accomplish. The review questions, concept blocks, eligibility criteria, and block-combination logic were prespecified before candidate appraisal and are reported in the Supplementary Methods, which constrains retrieval and makes the search reproducible by a second party. The grading rubric in Table 1 was written before any candidate was graded, so grading was rule application rather than impression, and every grade in Table 2 is traceable to a named study and a named finding rather than to a global judgment about a candidate. A separate search was performed for negative, null, and safety-adverse findings for each candidate, and the candidate-evidence log records those findings alongside supporting ones. The default-to-untested rule is an asymmetric safeguard against confirmation bias, because a narrative review ordinarily goes wrong by allowing a favorable account to fill evidentiary gaps, and a rule that converts every gap into an explicit untested grade removes the mechanism by which that happens. An adversarial self-audit followed, in which grading was repeated against the rubric with the declared aim of identifying a candidate that passes, and the failure of that attempt forms the basis for the negative conclusion reported in Section 7. The SANRA criteria were applied as a drafting self-audit (Baethge et al., 2019).

**TABLE 1:** Comparative methodologies for quantifying telomere length, telomere dysfunction, and telomere-associated signaling. The measured parameter is distinguished from the biological interpretation to prevent conflation into a singular depiction of telomere integrity.

Method or readout	Primary quantity	Principal strength	Principal limitation	Appropriate interpretation
Terminal restriction fragment (Southern)	Distribution and mean fragment length	Absolute size distribution; reference method	Large high-quality DNA; poor sensitivity for ultrashort ends; includes subtelomeric DNA	Broad length distributions, not damage signaling (Mender and Shay, 2015)
Quantitative PCR	Relative telomeric repeat abundance	High throughput, low DNA, large cohorts	Relative only; sensitive to DNA quality and plate effects; no single-cell resolution	Group-level relative length under strict QC (Cawthon, 2002)
Q-FISH/Flow-FISH	Per-chromosome or per-cell length	Single-cell and cell-type resolution	Technically demanding; probe and fixation effects	Cell-type-resolved length and heterogeneity (Lansdorp et al., 1996; Rufer et al., 1998)
STELA/TeSLA	Individual and shortest telomeres	Resolves the checkpoint-relevant lower tail	Chromosome-specific; labor-intensive; limited throughput	Shortest-end burden most linked to senescence onset (Baird et al., 2003; Lai et al., 2017)
TIF/TAF co-localization	DNA-damage foci at telomeres	Direct evidence of active dysfunction	Requires imaging; sensitive to fixation and scoring	Actively dysfunctional ends, not mere shortness (Hewitt et al., 2012)
Telomeric 8-oxoG; shelterin occupancy	Oxidative lesion; end protection	Mechanistic cause of dysfunction	Specialized assays; context-dependent	Damage or deprotection independent of length (Barnes et al., 2022)
TERRA; cytosolic telomeric DNA	Telomeric transcription; displaced DNA	Links telomere state to inflammation	Heterogeneous RNA species; attribution difficult	Signaling and paracrine potential (Azzalin et al., 2007; Siametis et al., 2024)

Abbreviations: 8-oxoG, 8-oxoguanine; DNA, deoxyribonucleic acid; Flow-FISH, flow cytometry combined with fluorescence *in situ* hybridization; PCR, polymerase chain reaction; Q-FISH, quantitative fluorescence *in situ* hybridization; QC, quality control; RNA, ribonucleic acid; STELA, single telomere length analysis; TAF, telomere-associated DNA, damage focus; TERRA, telomeric repeat-containing RNA; TeSLA, telomere shortest length assay; TIF, telomere dysfunction-induced focus.

**TABLE 2:** Interpretation of common senescence indicators in heterogeneous tissue. Each individual readout is inadequate; they must be interpreted in conjunction with alternative-state controls within the pertinent lineage.

Readout	What it indicates	Principal caveat and states it fails to exclude
p16 (CDKN2A)	Cell-cycle inhibition; senescence-associated	Expressed in some activated immune and progenitor cells; low sensitivity in several senescent populations (Gorgoulis et al., 2019)
p21 (CDKN1A)	Acute and chronic checkpoint activation	Transient after any DNA damage; does not establish persistence (Gorgoulis et al., 2019)
SA-beta-galactosidase	Lysosomal expansion	Positive in macrophages and some quiescent or highly confluent cells; pH-dependent (Hernandez-Segura et al., 2017)
Lamin B1 loss	Nuclear envelope remodeling	Also altered in quiescence and differentiation (Hernandez-Segura et al., 2017)
Ki-67 negativity	Non-proliferation	Shared with quiescence and terminal differentiation; not senescence-specific (Gorgoulis et al., 2019)
SASP factors	Secretory, inflammatory output	Overlaps with activation and acute injury; strongly context-dependent (Basisty et al., 2020)
Persistent DDR/TIF	Durable damage signaling	Stronger evidence when telomeric; requires imaging and controls (Hewitt et al., 2012)

Abbreviations: CDKN1A, cyclin-dependent kinase inhibitor 1A; CDKN2A, cyclin-dependent kinase inhibitor 2A; DDR, DNA-damage response; DNA, deoxyribonucleic acid; Ki-67, marker of proliferation Ki-67; p16, cyclin-dependent kinase inhibitor 2A protein product; p21, cyclin-dependent kinase inhibitor 1A protein product; SA-beta-galactosidase, senescence-associated beta-galactosidase; SASP, senescence-associated secretory phenotype; TIF, telomere dysfunction-induced focus.

None of these measures is equivalent to independent duplicate screening and extraction. A single investigator cannot measure agreement against himself, and an adversarial self-audit is weaker than adjudication by a second reviewer because the audit and the original grading share the same priors. Two commitments follow. The candidate-evidence log, the grading rubric as applied, and the extraction sheets are deposited so any reader can regrade the candidates and identify disagreements; the grades are therefore offered as reproducible claims rather than conclusions to be accepted. Independent duplicate grading by two investigators, blinded to candidate identity where feasible and reporting chance-corrected agreement (Landis and Koch, 1977), is the standard that a future implementation must meet, and it is incorporated into the prospective benchmarking design in Section 9.2. One structural point bears on interpretation. Single-investigator appraisal ordinarily inflates favorable conclusions, whereas this review concludes that no candidate passes, including the two most advanced candidates in the field and candidates in which the author holds no interest, so any residual bias is against the conclusion drawn rather than toward it.

A targeted narrative search of PubMed, Embase, Scopus, and Web of Science was conducted up to 15 July 2026, limited to English-language, peer-reviewed or recognized-preprint articles, using concept blocks for senescence phenotype, telomere dysfunction, immune accessibility and antigen presentation, bioinformatics and multi-omics, selectivity and safety, and translation. This was supplemented by backward and forward citation searching and targeted consultation of primary resource and dataset publications. Each search combined the senescence-phenotype block with the immune-accessibility block; the telomere, multi-omics, safety, and senolysis blocks were added as optional expanders rather than simultaneous requirements, preventing the exclusion of non-telomeric and non-bioinformatic evidence. Candidate-specific searches were performed separately for each targeted antigen. The Supplementary Methods detail the representative search strategy, block-combination logic, eligibility criteria, handling of overlapping datasets, methodology for identifying contradictory evidence, and the SANRA self-assessment. Because this is a targeted narrative search rather than a systematic review, completeness and adherence to PRISMA screening protocols are not claimed (Bramer et al., 2018; Page et al., 2021; Rethlefsen et al., 2021; Miake-Lye et al., 2016). The supplementary methods also include a candidate-evidence log for the studies evaluated in Tables 3 and 10. The primary unit of analysis is the candidate-evidence relationship rather than the publication itself. Negative or contradictory findings are retained, as they may indicate cell-type restriction, inducer dependence, or genuine irreproducibility. Quantitative meta-analysis was deemed inappropriate because measures such as differential expression, peptide abundance, surface intensity, cytotoxicity, and lifespan endpoints are not comparable. Instead, the synthesis emphasizes descriptive associations, mechanistic perturbations, direct evidence from presentation, functional recognition, and therapeutic efficacy, avoiding stronger causal claims from less direct evidence (Snyder, 2019; Wilkinson et al., 2016).

**TABLE 3:** Report-Level Evidence Map for Published Strategies Addressing Immune Recognition or Elimination of Senescence-Associated Cells. Each row represents a distinct report, including those from overlapping research groups and reports pertaining to different targets. “Replication and triangulation” delineate within-study repetition, cross-species triangulation, and verification by an independent laboratory. A comprehensive risk-of-bias assessment was not conducted for each report; the reporting notes column provides details on the study design in which the methodologies were evaluated (Eskiocak et al., 2026; Amor et al., 2020; Suda et al., 2021), and other reports are marked as not subject to individual auditing. The 2024 correction to Amor et al. (2020), which replaced a duplicated micrograph without altering the conclusions, is duly documented. Selected quantitative effects and sample sizes are presented for the worked-example and GPNMB-variant candidates within the Supplementary Candidate-Evidence Log; remaining reports are summarized qualitatively.

Report	Target; intended route	Species, model, and design	Replication and triangulation	Reported design features/reporting notes	Principal biological limitation
Frescas et al., 2017	Oxidized vimentin; antibody discovery	Human fibroblasts; oxidative surface-epitope discovery; no *in vivo* model	None (discovery only)	Discovery report; no *in vivo* cohort; design safeguards not applicable	No senolysis or safety data
Amor et al., 2020	uPAR; CAR-T (engineered cell)	Intervention in mouse lung adenocarcinoma and CCl4/diet liver fibrosis; anti-uPAR CD28^−^CD3ζ CAR, 3 × 10^6^ cells. Human tissue validation (expression only) in liver fibrosis, carotid endarterectomy, and pancreatic intraepithelial neoplasia (PanIN) specimens	Within-study repetition (2 experiments); cross-model triangulation across the 2 mouse intervention models; human tissue validation is separate from the intervention evidence	n = 3 mice/group, two independent experiments; randomization and masking not reported; a 2024 Author Correction (Amor et al., 2024b) replaced a duplicated micrograph and stated the conclusions were unchanged	uPAR on injury/myeloid cells; no prespecified healthy-tissue safety margin
Amor et al., 2024a	uPAR; CAR-T (engineered cell)	Aged mice; low-dose anti-uPAR CAR-T; metabolic endpoints	Within-study repetition; overlapping group (not independent-lab)	Design safeguards not individually audited; see primary source	Safety margin across healthy compartments not established
Eskiocak et al., 2026	uPAR; CAR-T (engineered cell)	Young and aged mice; intestinal aging; 15 Gy abdominal-irradiation injury/regeneration challenge; mucosal immune readouts	Within-study repetition; overlapping group	Mice not randomized; one observer blinded, data collection and analysis otherwise not blinded; no formal sample-size calculation; defined exclusions applied	Physiological-challenge (regeneration) benefit shown; not a full multi-compartment safety margin
Suda et al., 2021	GPNMB; native-surface antibody vaccine (antibody/NK-ADCC)	Mouse HFD, ApoE-KO atherosclerosis, progeroid; extracellular-domain peptide vaccine vs. control vaccine	Within-study repetition across models	Control-vaccine and Gpnmb-ablation controls; randomization and masking not reported in the examined text	Population/tissue-level senescence evidence; human immunogenicity and safety margin untested
Suda et al., 2022	GPNMB biology (not a vaccine study)	GPNMB depletion and overexpression *in vitro* and *in vivo*; lysosomal integrity, vascular function, and cellular lifespan; no vaccination tested	Mechanistic study; same group; not vaccine replication	Design safeguards not individually audited; see primary source	Supports GPNMB biology and context-dependent protective and safety-liability considerations, not vaccine efficacy or replication of Suda et al., 2021
Cao et al., 2024	GPNMB; dendritic-cell vaccine (cellular, CD8)	Mouse HFD young/aged and natural aging; Gpnmb-CP-pulsed DC vaccine vs. protein vaccine	Within-study repetition	Design safeguards not individually audited; see primary source	Distinct cellular (CD8) route; protein vaccine ineffective in aged mice; safety margin untested
Zhang et al., 2025	GPNMB; nanovaccine GK-NaV (cellular, CD8)	Mouse HFD-induced progeroid; K36-Gpnmb self-assembled nanovaccine; DC uptake, CD8 activation	Within-study repetition	Design safeguards not individually audited; see primary source	Distinct cellular route; should not be merged with the antibody-route Suda vaccine; safety margin untested
Yoshida et al., 2020	CD153; peptide vaccine	Mouse HFD; adipose senescent T cells; complement-dependent cytotoxicity	Within-study repetition	Design safeguards not individually audited; see primary source	CD153 on non-senescent activated T cells; narrow senotype coverage
Yu et al., 2025	CD38; peptide vaccine	Aged mice; myeloid immunometabolic cells	Within-study repetition	Design safeguards not individually audited; see primary source	CD38 has essential immunometabolic roles; healthy-state margin untested
Yang et al., 2023	NKG2D ligands; CAR-T (engineered cell)	Mouse; stress-ligand-positive senescent cells	Within-study repetition	Design safeguards not individually audited; see primary source	Ligands are general stress signals on activated healthy cells; selectivity unresolved
Deng et al., 2024	NKG2D ligands; CAR-T (engineered cell)	Mouse and non-human primate; stress-ligand-positive cells; tolerability	Cross-species triangulation (mouse + NHP)	Design safeguards not individually audited; see primary source	Selectivity unresolved; general stress-ligand expression
Majewska et al., 2024	PD-L1/PD-L2; checkpoint/antibody	Mouse therapy-induced senescence; checkpoint modulation	Within-study repetition	Design safeguards not individually audited; see primary source	Checkpoints inducible and not senescence-exclusive
Iltis et al., 2025	GD3; antibody	Mouse therapy-induced senescence; NK-linked mechanism	Within-study repetition (different target from Majewska)	Design safeguards not individually audited; see primary source	GD3 absent from several oncogene-induced senescent states
Ichim et al., 2025	Senescent-cell lysate; DC vaccine	Mouse tumor models; polyclonal response	Within-study repetition	Design safeguards not individually audited; see primary source	Mediating antigens undefined; not a defined-target test

Abbreviations: ADCC, antibody-dependent cellular cytotoxicity; ApoE-KO, apolipoprotein E knockout; CAR, chimeric antigen receptor; CAR-T, chimeric antigen receptor T cell; CCl4, carbon tetrachloride; CD, cluster of differentiation; CD3-zeta, cluster of differentiation 3 zeta signaling chain; DC, dendritic cell; GD3, disialoganglioside 3; GPNMB, glycoprotein nonmetastatic melanoma protein B; gpnmb, murine glycoprotein nonmetastatic melanoma protein B gene; Gy, gray; HFD, high-fat diet; IgM, immunoglobulin M; NHP, non-human primate; NK, natural killer; NKG2D, natural killer group 2 member D; PanIN, pancreatic intraepithelial neoplasia; PD-L1, programmed death-ligand 1; PD-L2, programmed death-ligand 2; uPAR, urokinase-type plasminogen activator receptor.

## 3 Telomere dysfunction as an upstream driver of pathological senescence

Telomere dysfunction should be regarded as a mechanistically defined pathway leading to cellular senescence rather than being a mere synonym for aging. While replicative erosion can produce critically short chromosome ends, dysfunction may also result from the loss of end protection, oxidative damage, replication fork failure, transcriptional stress, or R-loop accumulation without significant telomere shortening. Consequently, a population with reduced mean telomere length does not necessarily have a high burden of damaged telomeres. Distinguish between chronological age, mean telomere length, and senescence-associated gene expression, as these are independent parameters and should not be used interchangeably (Karlseder et al., 2002; d'Adda di Fagagna et al., 2003; Hewitt et al., 2012; Barnes et al., 2022). Figure 3 illustrates the continuum from protected chromosome termini through telomeric stress, persistent DNA damage response, and stabilized senescence to a pathological senescence phenotype, and it also delineates length-distribution assays from direct dysfunction assays.

**FIGURE 3:**
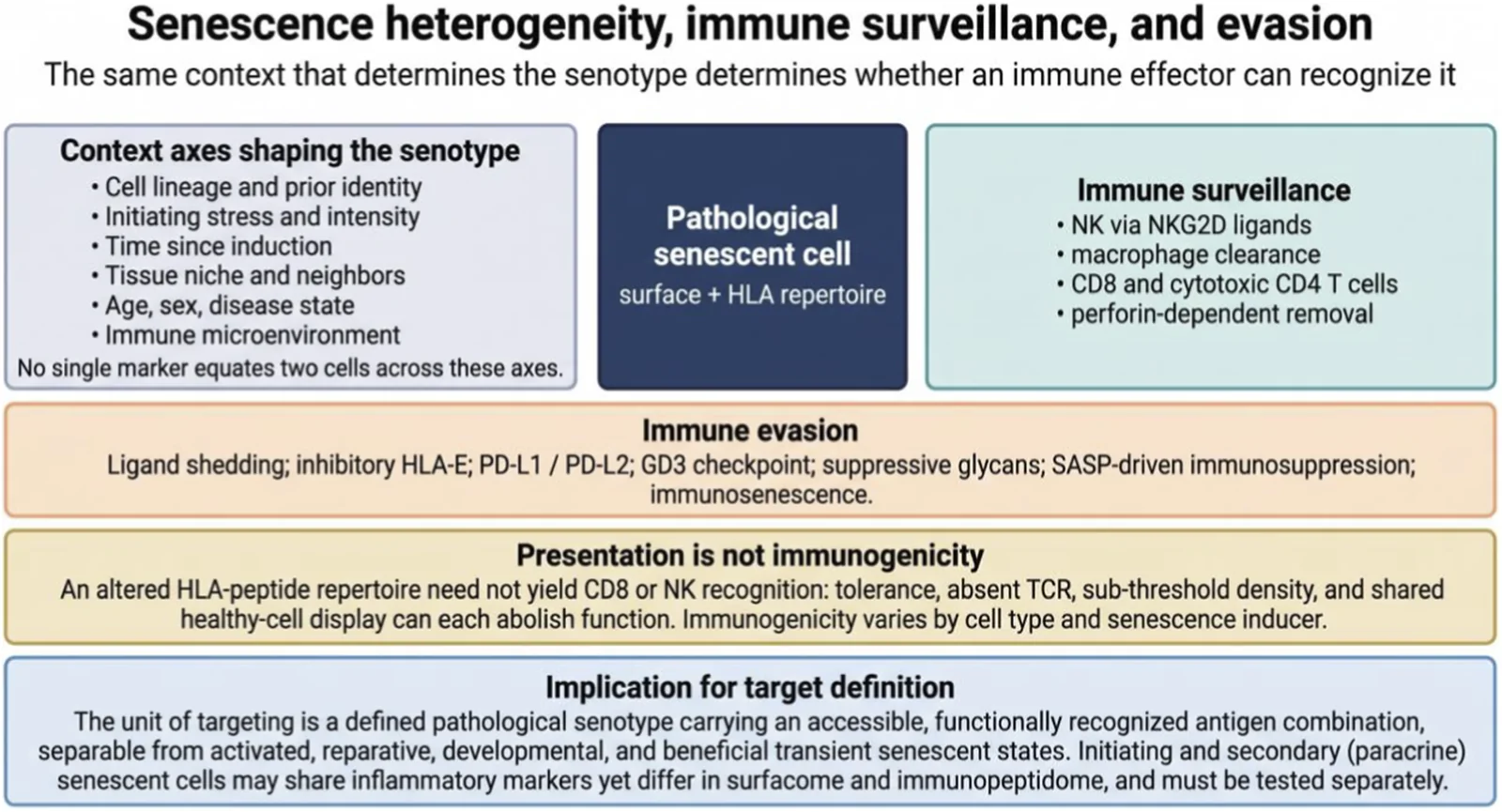
Senescence heterogeneity, immune surveillance, and evasion. The contextual axes that define the senotype also determine whether an immune effector can recognize it. Presentation differs from immunogenicity, and the target entity is a specific pathological senotype that can be distinguished from activated, reparative, developmental, and beneficial transient states. Abbreviations: CD4 and CD8, cluster of differentiation 4 and 8; GD3, disialoganglioside 3; HLA, human leukocyte antigen; HLA-E, human leukocyte antigen E; NK, natural killer; NKG2D, natural killer group 2 member; D; PD-L1 and PD-L2, programmed death-ligand 1 and 2; SASP, senescence-associated secretory phenotype; TCR, T-cell receptor.

### 3.1 Architecture, protection, and the length-versus-function distinction

Mammalian telomeres consist of tandem TTAGGG repeats, a G-rich overhang, higher-order structures, including the T-loop, and the six-subunit shelterin complex, in which telomeric repeat-binding factor 1 (TRF1) and telomeric repeat-binding factor 2 (TRF2) bind to double-stranded repeats. Protection of telomeres 1 (POT1) recognizes single-stranded DNA, and TRF1-interacting nuclear factor 2 (TIN2) and TIN2-interacting protein 1 (TPP1) connect the respective modules. Protection represents an active signaling state rather than a passive consequence of terminal repeats: TRF2 inhibits ATM-associated recognition and classical end joining, while POT1 represses ATR signaling. Removing shelterin exposes chromosome ends to normally suppressed pathways, suggesting that shelterin’s functional status can matter as much as telomere length (de Lange, 2005; 2018; Sfeir and de Lange, 2012).

The shortest telomeres may matter more than the average telomere length within a population. Individual chromosome ends show significant allelic and cell-to-cell variation, and senescent cell cultures may contain ultrashort telomeres that bulk measurement techniques underrepresent; accordingly, researchers developed single-telomere and shortest-length assays to address this limitation. Modulating TRF2 altered the length threshold at which cells undergo senescence, suggesting that cells can tolerate additional sequence loss when shortened telomere ends remain protected, whereas acute uncapping triggers cell cycle arrest without extensive erosion (Baird et al., 2003; Lai et al., 2017; Harley et al., 1990). Furthermore, telomeres transcribe telomeric repeat-containing RNA (TERRA), creating a mechanistic interface among telomere structure, transcriptional stress, R-loop formation, DNA-damage signaling, and innate immune sensing (Azzalin et al., 2007; Siametis et al., 2024).

### 3.2 Persistent DNA-damage response, inflammation, and paracrine propagation

Telomeric lesions trigger a DNA-damage response that remains persistent because telomeric damage is repaired inefficiently, sustaining checkpoint signaling and maintaining cell cycle arrest (Fumagalli et al., 2012; Rossiello et al., 2022). A regulatory feedback loop exists linking checkpoint signaling, mitochondrial dysfunction, and reactive oxygen species. Because telomeric DNA is particularly vulnerable to oxidative guanine lesions, this feedback mechanism can turn an initially localized telomeric defect into a more extensive and persistent stress response (Passos et al., 2010; Barnes et al., 2022). Senescent cells release chromatin fragments into the cytoplasm, where cyclic guanosine monophosphate-adenosine monophosphate (GMP-AMP) synthase triggers stimulator of interferon genes (STING)-dependent inflammatory responses. Cytosolic DNA should be regarded as a mechanistic class comprising nuclear, mitochondrial, and, in specific contexts, telomeric DNA, rather than being attributed to a single source (Dou et al., 2017; Gluck et al., 2017).

Direct evidence for a telomere-specific paracrine mechanism comes from a transcription-stress model in which loss of transcription elongation factor IIS (TFIIS) induces polymerase stalling, telomeric R-loop accumulation, uncapping, and cellular senescence. This process involves cytoplasmic telomeric DNA and extracellular vesicles containing telomeric DNA, which activate inflammatory signaling and induce senescence in recipient cells (Siametis et al., 2024). Moreover, telomere dysfunction triggers inflammation through an ATM-cellular Abelson tyrosine-protein kinase-Yes-associated protein 1 (ATM-c-ABL-YAP1) axis, promoting interleukin-18 signaling and showing that inflammatory responses from dysfunctional telomeres are not restricted to a single pathway (Chakravarti et al., 2020). This propagation is especially important in antigen discovery, since secondary paracrine senescent cells may display inflammatory markers identical to those of primary telomere-dysfunctional cells but lack the primary lesion. Therefore, researchers must validate a telomere-associated target in both primary and secondary populations rather than assuming they share the same surfaceome (Hewitt et al., 2012; Siametis et al., 2024).

### 3.3 Measuring telomere length and dysfunction

Align the measurement strategy with the biological question rather than treating assays as interchangeable. Bulk assays estimate central tendency; shortest-telomere assays probe the lower tail; imaging methods reveal heterogeneity at the chromosome or cell level; and telomere-associated damage assays assess whether an end actively participates in a DNA-damage response. Table 4 delineates the measured quantity from the biological inference, as overinterpretation may occur when relative abundance, shortest-end burden, telomeric damage, and telomerase activity are collectively referred to as telomere health (Cawthon, 2002; Baird et al., 2003; Mender and Shay, 2015; Lai et al., 2017).

**TABLE 4:** Analytical modalities and their evidentiary value in the discovery of seno-antigens. Directness increases from group-level association to functional recognition.

Modality	Question answered	Directness	Principal limitation
Bulk transcriptomics	Group-level differential abundance	Low	Confounds composition with state change (Love et al., 2014)
Single-cell/single-nucleus	Lineage- and state-resolved expression	Moderate	Dropout, ambient RNA, pseudoreplication (Luecken and Theis, 2019)
Spatial transcriptomics/proteomics	Niche and immune proximity	Moderate	Resolution and segmentation limits
Total, secretome, EV proteomics	Protein production and secretion	Moderate-high	Does not establish surface exposure (Basisty et al., 2020)
Cell-surface proteomics	Extracellular accessibility	High	Sensitive to labeling and topology (Wollscheid et al., 2009; Bausch-Fluck et al., 2018)
HLA immunopeptidomics	Naturally presented ligands	High	Presentation is not immunogenicity (Purcell et al., 2019)
Functional immune assays	Route-specific antigen-dependent recognition and senolytic clearance	Highest	Requires modality-appropriate effectors and antigen-dependence controls; HLA matching only for HLA-restricted assays (Goyer et al., 2026)

Abbreviations: EV, extracellular vesicle; HLA, human leukocyte antigen; RNA, ribonucleic acid.

For this review, a population is classified as telomere-dysfunction-associated pathological senescence only when three separable propositions hold: the cells have entered a persistent senescent state, telomere dysfunction is present and mechanistically linked to that state, and persistence has deleterious consequences in the relevant tissue. Antigen accessibility and healthy-tissue selectivity are subsequent targeting requirements rather than criteria that establish the underlying state. Telomere dysfunction therefore offers a stronger mechanistic anchor than chronological age or a generic senescence score, but only when measured directly and interpreted alongside persistence and pathology (Gorgoulis et al., 2019; Rossiello et al., 2022; Purcell et al., 2019).

### 3.4 Telomere maintenance, replicative limits, and therapeutic context

Most somatic cells lack sufficient telomerase activity to prevent progressive telomere shortening, leading to replicative senescence after repeated divisions once one or more chromosome ends become critically short. In contrast, germline, stem, and numerous cancer cells maintain telomeres via telomerase activity or the alternative lengthening of telomeres (ALT) pathway. This biological asymmetry has two significant implications for antigen discovery. First, the pathological cell populations of interest are generally telomerase-low or telomerase-negative cells that have accumulated dysfunction, distinguishing them from telomerase-active progenitors that must be preserved. Second, telomere maintenance status itself may serve as a discriminating variable, as telomerase reactivation or ALT engagement modifies the damage landscape and influences the subsequent cellular state (Harley et al., 1990; Kim et al., 1994; Herbig et al., 2004).

Telomere-initiated senescence activates the same p16-RB and p53-p21 pathways as other senescence mechanisms; however, the timing and reversibility differ. Oncogene-induced senescence results in telomeric and non-telomeric DNA damage, as evidenced by telomeric DNA damage foci in premalignant human lesions, thereby linking telomere dysfunction to a tumor-suppressive barrier *in vivo*. Conversely, loss of p16 may allow cells to escape telomere-driven arrest. These findings reinforce the notion that telomere dysfunction constitutes one entry point into a common senescence program rather than representing a distinct terminal state. Moreover, a telomere-anchored target should be validated at the resultant senotype level rather than inferred from the initiating lesion (Suram et al., 2012; Jacobs and de Lange, 2004; Gorgoulis et al., 2019). These observations suggest assessing telomere maintenance status, damage burden, and effector pathway engagement together. This is because a candidate antigen that correlates with telomerase-low, dysfunctional cells offers greater selectivity against telomerase-active, healthy progenitors than an antigen based solely on a generic senescence score.

### 3.5 Discriminating mechanistic strata within one tissue

Telomere-driven and inflammatory or paracrine senescence coexist in the same tissue and often in the same lesion, and a discovery dataset that scores senescence with a general state signature cannot tell them apart. The discrimination is empirical and rests on four contrasts, each measurable in human tissue.

The first contrast is the lesion itself. Telomere-driven senescence carries DNA-damage-response foci that co-localize with telomeric signal, detectable as telomere-associated foci by combined immunofluorescence and fluorescence *in situ* hybridization, whereas paracrine senescence carries either non-telomeric damage foci or no persistent damage response at all, its arrest being driven by inflammatory signaling rather than by a genomic lesion (Hewitt et al., 2012; Fumagalli et al., 2012). This is the primary discriminator and the only one that operates at single-cell resolution in intact tissue. The second contrast is the distribution rather than the mean of telomere length, since telomere-driven strata are characterized by ultrashort telomeres that single-telomere length analysis resolves and that averaging conceals entirely (Baird et al., 2003).

The third contrast is the dependence of the state on a continuing external signal. Telomeric damage persists because the lesion is refractory to repair, so the damage response does not resolve and the state becomes cell-autonomous once established (Fumagalli et al., 2012). Paracrine senescence, by contrast, is induced and sustained by interleukin-1 and nuclear factor kappa B-dependent signaling from neighboring cells and depends on the continuation of that signal (Acosta et al., 2013; Nelson et al., 2012). The fourth contrast follows directly and is a perturbation test: interruption of the paracrine signal by interleukin-1 receptor blockade or Janus kinase inhibition attenuates the paracrine stratum while leaving telomere-associated foci intact, and telomere-directed interventions that reduce telomeric damage signaling attenuate the telomere-driven stratum specifically (Barnes et al., 2022; Oppezzo et al., 2026).

The spatial signatures also differ and can be tested with existing platforms. Paracrine senescence forms clusters radiating from a primary focus because the inducing signal is local and diffusible, whereas telomere-driven senescence tracks replicative history and lineage rather than proximity and is distributed according to turnover. The consequence for antigen discovery is direct. If a candidate antigen is enriched only in the paracrine stratum, a telomere-stratified target product profile is the wrong profile for that candidate, and the reverse holds equally. This is the operational reason that the telomere axis is a stratifier rather than a criterion and the reason that unmatched data leave the axis graded untested rather than presumed satisfied.

## 4 Senescence heterogeneity and immune surveillance

Cellular senescence is a group of stress-adapted states characterized by sustained proliferative arrest, with heterogeneity in transcriptional activity, chromatin configuration, metabolic processes, secretory behavior, surface marker expression, antigen processing, and vulnerability to immune elimination. Cellular lineage, the initial lesion, the severity and duration of the stress response, the time elapsed since induction, and the tissue microenvironment shape the phenotype. Consequently, a single conventional marker cannot confirm that 2 cells are in equivalent senescent states (Hernandez-Segura et al., 2017; Gorgoulis et al., 2019; Sanborn et al., 2025). The term ‘senotype’ is employed to describe a context-specific senescent phenotype encompassing intrinsic components, such as cell-cycle arrest, checkpoint activation, persistent damage signaling, and organelle dysfunction, and extrinsic components, including senescence-associated secretory phenotype (SASP) factors, extracellular vesicles, surface ligands, immune checkpoints, and the HLA-associated peptide repertoire (Hernandez-Segura et al., 2017; Basisty et al., 2020). Figure 4 illustrates this heterogeneity within the surrounding immune ecosystem.

**FIGURE 4:**
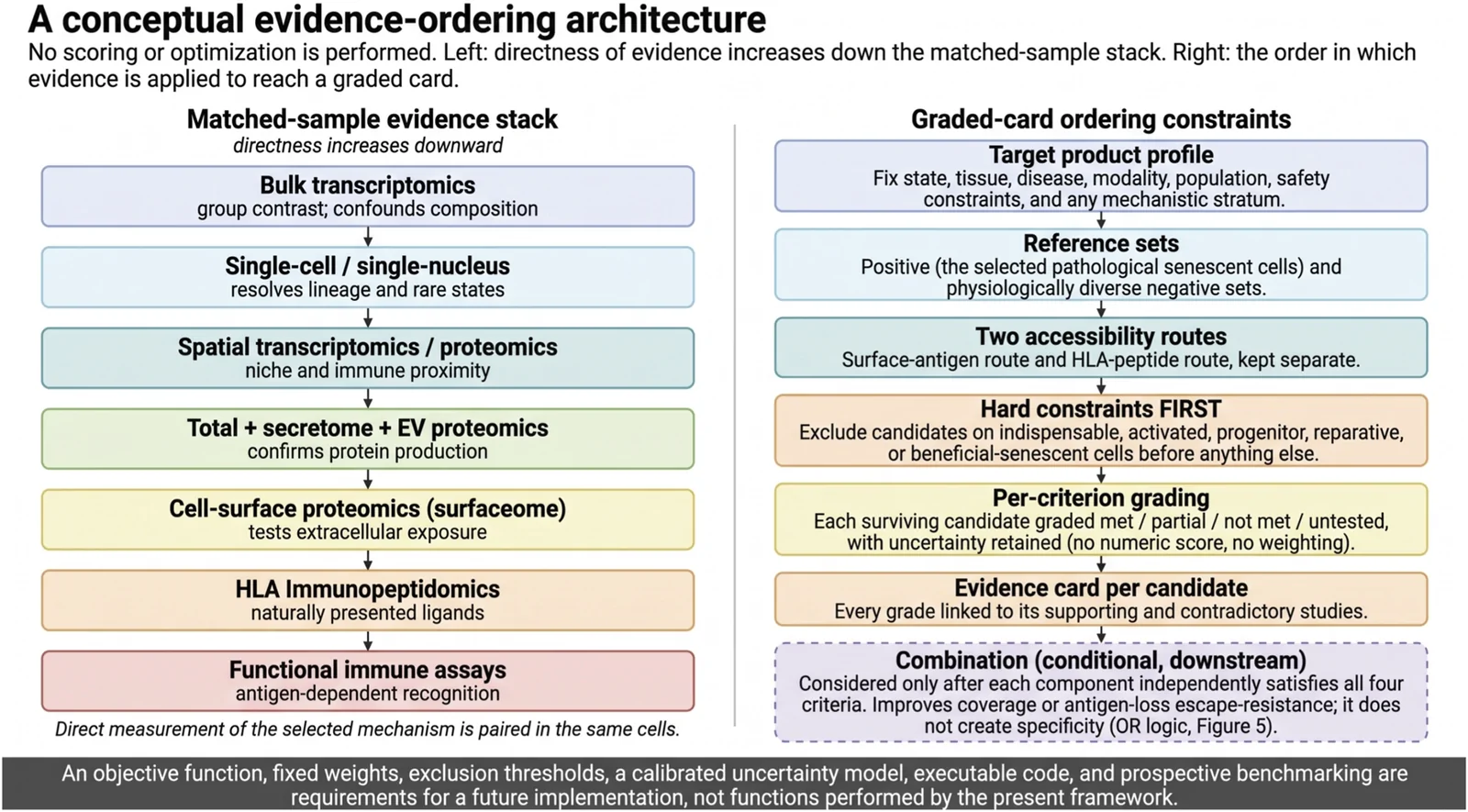
A conceptual evidence-ordering architecture. The matched-sample stack on the left orders evidence by directness, with the selected mechanism measured directly in the same cells. The right column shows the order in which evidence is applied: a target product profile, positive and negative reference sets, two separated accessibility routes, hard exclusions applied before anything else, per-criterion grading with retained uncertainty rather than a numeric score, and an evidence card per candidate. Combination is shown as a conditional, downstream step that follows only after each component independently satisfies the four criteria. An objective function, weights, thresholds, a calibrated uncertainty model, code, and prospective benchmarking are requirements for a future implementation, not functions the present framework performs. Abbreviations: EV, extracellular vesicle; HLA, human leukocyte antigen; OR, Boolean disjunction operator denoting a recognition rule satisfied when any single component is recognized.

### 4.1 Limitations of canonical markers and context-dependent function

No single marker, including p16, p21, senescence-associated beta-galactosidase, Lamin B1 loss, or absence of proliferation markers, is sufficient in isolation, as its expression, sensitivity, and specificity vary by cell type, inducer, time point, tissue, and platform. Table 5 summarizes how to interpret common readouts in heterogeneous tissue and highlights which alternative states they fail to exclude. In practice, never deem a candidate antigen indicative of senescence unless the source population meets a multi-feature definition, with appropriate controls for alternative states (Gorgoulis et al., 2019; Hernandez-Segura et al., 2017; Suryadevara et al., 2024).

**TABLE 5:** Prespecified healthy-state counter-screening panel for criterion four. A met grade requires every compartment to pass on density or on functional challenge with an antigen-dependence control. A partial grade must state the number of compartments passed and name those omitted. The margin is evaluated at the worst-case donor, time point, and activation state, not the mean.

Compartment	Model system	Readout	Pass condition	Failure mode detected
Tissue repair	Human wound-edge fibroblasts and keratinocytes at days 3, 7, and 14; organotypic wound model; *in vivo* dermal wounding and a regeneration challenge such as partial hepatectomy	Antigen density on intact cells across the time course; wound closure, tensile strength, and regenerative index under therapy	Density at least tenfold below the route threshold at every time point, or unimpaired repair with an antigen-dependence control	Transient induction on reparative cells, invisible in resting-tissue atlases
Acute inflammation	Monocytes, macrophages, and endothelial cells exposed to lipopolysaccharide, tumor necrosis factor, and interferon gamma, sampled at 6, 24, and 72 h	Antigen density; for peptide routes, re-elution of the ligand under interferon exposure	Density at least tenfold below threshold at every stimulus and time point	Interferon-driven upregulation of surface antigen and of antigen presentation
Infection	Acute viral challenge *in vivo*; latent herpesvirus reactivation model; human peripheral cells during acute infection where available	Pathogen clearance, viral load, and antigen density on responding cells	No impairment of clearance or of latency control, with an antigen-dependence control	Elimination of cells required for host defense, the failure mode with the longest latency
Activated immune cells	T cells activated with anti-CD3 and anti-CD28 at 24 and 72 h; activated natural killer cells; plasmablasts; activated tissue-resident macrophages	Antigen density on each activated subset; vaccination response *in vivo*	Density at least tenfold below threshold, and preserved antibody and T-cell responses to an unrelated vaccine	Activation-induced expression producing progressive immune attrition
Progenitor populations	CD34-positive hematopoietic progenitors; intestinal crypt LGR5-positive cells; satellite cells; epidermal basal cells; alveolar type 2 cells	Antigen density; colony-forming and organoid-forming capacity under therapy; hematopoietic recovery after myelosuppressive stress	Density at least tenfold below threshold and preserved clonogenic capacity	Stem and progenitor depletion, with delayed and potentially irreversible presentation
Beneficially senescent cells	Transient wound-associated senescent fibroblasts and endothelium; oncogene-induced senescent cells serving a tumor-suppressive role; developmental programmed senescence surrogates	Antigen density; wound remodeling quality; long-term tumor incidence and surveillance	Density at least tenfold below threshold, or no increase in tumor incidence over the full observation period	Removal of adaptive senescence, including loss of tumor suppression
Aged healthy tissue	Age-matched non-diseased donor tissue from the target organ and from organs not intended for treatment; aged rather than young animals	Antigen density and burden of antigen-positive cells; systemic tolerability in aged hosts	Density at least tenfold below threshold in non-target organs, with antigen-positive burden quantified in the target organ	Widespread low-level expression, harmless per cell but unacceptable in aggregate

Abbreviations: CD3, cluster of differentiation 3; CD28, cluster of differentiation 28; CD34, cluster of differentiation 34; LGR5, leucine-rich repeat-containing G-protein-coupled receptor 5.

Function is equally context-dependent. Senescence plays a role in embryonic patterning, wound healing, fibrosis resolution, and tumor suppression. Lineage-resolved investigations show that certain p16-associated populations support tissue repair or integrity rather than contributing to pathology. A molecular characteristic that is highly enriched in irradiated fibroblasts may be absent from endothelial cells with dysfunctional telomeres or secondary senescent cells. Furthermore, a marker broadly associated with senescence may also identify activated, differentiated, or reparative cells. Therefore, the pertinent target is a specific pathological senotype that presents an accessible, immunologically actionable antigen combination, rather than an abstract, universal senescent cell (Munoz-Espin et al., 2013; Demaria et al., 2014; Grosse et al., 2020; Zhao et al., 2024).

### 4.2 Surveillance, evasion, and the presentation-immunogenicity gap

Both innate and adaptive immune mechanisms recognize senescent cells. Natural killer cells respond to stress-induced natural killer group 2 member D (NKG2D) ligands; macrophages phagocytose opsonized or ligand-marked cells; and cytotoxic CD4 and CD8 T lymphocytes participate in specific contexts. Senescent cells evade these immune responses by shedding activating ligands, expressing inhibitory HLA-E and checkpoint molecules such as programmed death-ligand 1 (PD-L1) and programmed death-ligand 2 (PD-L2), displaying the ganglioside disialoganglioside 3 (GD3), and secreting immunosuppressive factors, with age-related immunosenescence further impairing immune surveillance (Ovadya et al., 2018; Pereira et al., 2019; Munoz et al., 2019; Iltis et al., 2025).

The most consequential point for a bioinformatics framework is that presentation is not immunogenicity. Human data show that an altered immunopeptidome need not yield effective CD8 or natural-killer recognition, that immunogenicity depends on cell type and inducer, and that predicted binding cannot infer a responsive T-cell repertoire, overcome tolerance, or establish healthy-tissue safety. Functional recognition and cross-reactivity must therefore remain experimentally measured outputs rather than labels generated from the same features used to train a presentation model (Purcell et al., 2019; Goyer et al., 2026; Deng et al., 2024). This constraint defines the boundary between what computational prioritization can promise and what only prospective experiments can deliver, and it motivates the two-route verification design developed in Section 6.

### 4.3 SASP heterogeneity, extracellular vesicles, and surveillance dynamics

The senescence-associated secretory phenotype is inherently heterogeneous and varies over time, rather than constituting a fixed signature. Quantitative proteomic analysis across various inducers shows that the secretome differs by cell type and stimulus, with a core set of shared factors coexisting alongside inducer-specific components. Furthermore, soluble proteins, matrix-remodeling enzymes, and extracellular vesicles each contribute unique signals. This has direct implications for target identification, as a secreted or vesicle-associated protein may indicate the secretory activity of a cell population without being present on the plasma membrane in a form accessible to antibodies or engineered receptors (Basisty et al., 2020; Cvjetkovic et al., 2016).

Extracellular vesicles complicate research in two significant ways. They transport surface and luminal proteins that may be present in bulk or surface preparations but do not accurately represent the intact cell surfaceome of the originating population. Additionally, they transmit senescence and immunomodulatory signals to neighboring cells, leading to secondary populations whose markers overlap with those of the primary population but whose antigenic surface characteristics may differ. Consequently, a discovery pipeline must distinguish cell-intrinsic surface antigens from vesicle-derived contaminants and verify candidates on intact cells rather than on lysates or vesicle-enriched fractions (Cvjetkovic et al., 2016; Siametis et al., 2024). The temporal aspect is equally crucial: early, reversible, and late, stabilized senescent states vary in secretory output and immune visibility. Therefore, a marker validated at one time point may misclassify the same lineage at another (Hernandez-Segura et al., 2017; Saul et al., 2022).

Surveillance operates dynamically and reciprocally. Efficient initial elimination by natural killer cells and macrophages can resolve transient senescence during processes such as wound healing and fibrosis mitigation. Conversely, cellular populations that endure beyond this initial phase do so for several reasons that are not mutually exclusive, including a permissive tissue microenvironment, declining effector function in the aged immune system, physical sequestration from effector cells, and selection for cell-intrinsic immune evasion. Only the last of these implies that the persistent population has diverged antigenically from the transient population, and the magnitude of that effect is an open empirical question rather than an established property of persistent senescence. To the extent that immune evasion does contribute, a target antigen predominantly expressed on readily cleared transient cells may be underrepresented on the persistent cell populations that principally contribute to disease progression. Accordingly, the framework emphasizes identifying antigens validated on persistent cells and treats standardized, cross-platform definitions of senescence as essential for comparing candidate targets across studies (Krizhanovsky et al., 2008; Sagiv et al., 2013; Ovadya et al., 2018; Gurkar et al., 2023).

### 4.4 Temporal stability as a measured property of the antigen

Because senescent states change over time, the antigen that identifies a population at one time point may not identify it at another, and the framework treats temporal stability as a property to be measured rather than assumed. Three requirements follow.

Criterion one requires explicit temporal specificity. Durable arrest must be demonstrated at two time points separated by an interval relevant to the disease, by lineage tracing, or by measurement in chronic human lesions rather than in acute induction models; reversibility on withdrawal of the inducer is a disqualifying finding for the persistent stratum rather than a partial grade.

Criterion two requires demonstrating accessibility at the late, stabilized time point. The therapeutic target is the persistent population, and an antigen detected only in early senescence identifies the transient population that immune surveillance already clears efficiently. This carries an implication that the field has not confronted directly. Persistent senescent cells are those not cleared during the initial phase of surveillance, and several mechanisms can explain their persistence. A permissive tissue microenvironment, declining effector function in the aged immune system, physical sequestration from effector cells, and selection for cell-intrinsic immune evasion are all plausible contributors, and they are not mutually exclusive. Only the last of these implies that the persistent population has diverged antigenically from the early senescent population. To the extent that immune evasion does contribute, the antigens most easily detected on early senescent cells will be enriched for those least likely to remain on the cells that drive disease, but the size of that effect is an open empirical question rather than an established property of persistent senescence. Whichever mechanism predominates, a candidate validated only at an early time point may be optimized against a population that does not persist, and early-only evidence is therefore graded as nomination rather than as accessibility.

The evidence card records antigen density at early and late time points wherever longitudinal data exist, along with the direction of change, since declining antigen predicts escape under therapeutic pressure, while an antigen that rises as the state stabilizes is preferable on both efficacy and safety grounds. The HLA immunopeptidome is handled under the same rule with one additional requirement. Because the presented repertoire shifts with the secretory program, with proteasome composition, and above all with interferon exposure, a peptide nominated from an early state, from a stimulated state, or from a cultured model must be re-eluted from the stabilized state in the disease context before criterion two is graded as met, and immunopeptidomic data acquired under interferon stimulation must be labeled as such (Hernandez-Segura et al., 2017; Saul et al., 2022). This requirement disqualifies a substantial fraction of existing immunopeptidomic evidence in senescence from supporting a met grade, which is a correct consequence rather than an inconvenience.

## 5 Current evidence for immune-mediated senolysis

Genetic ablation established the rationale for removing selected senescence-associated populations before a clinically applicable target existed, and pharmacological studies demonstrated that benefits could be achieved without continuous genetic deletion. These studies also emphasized the limitations of systemic drugs whose activity relies on variable intracellular survival pathways (Baker et al., 2011; 2016; Zhu et al., 2015; Xu et al., 2018; Kirkland and Tchkonia, 2020). Immune-mediated senolysis translates this principle into a target-dependent therapeutic approach. The most compelling claim, selective immune senolysis, necessitates more than just improved glucose tolerance, reduced fibrosis, or marker expression. It requires an accessible antigen enriched in a specific pathological senotype, an antigen-dependent immune response, reduction of orthogonally defined senescent cells within the relevant tissue, a causal connection between clearance and therapeutic benefit, and the preservation of essential healthy and beneficially senescent cell populations (Gorgoulis et al., 2019; Purcell et al., 2019; Goyer et al., 2026).

These requirements can be organized as sequential evidence gates: target definition and accessibility; an antigen-dependent mechanism confirmed by blocking, knockout, or antigen-negative controls; direct measurement of senescent-cell burden at single-cell resolution; mechanistic linkage between clearance and benefit; selectivity and long-term safety under physiological challenge; and durability paired with controllability. The published strategies below satisfy different subsets of these gates, and Table 3 records their reported evidence and principal limitations (Amor et al., 2020; Amor et al., 2024a; Suda et al., 2021; Yoshida et al., 2020; Iltis et al., 2025).

### 5.1 Active vaccination against defined targets

An early antibody-discovery study isolated a natural IgM antibody that recognizes an oxidized, malondialdehyde-modified form of vimentin exposed on senescent cells, demonstrating that senescence-associated oxidative remodeling can generate surface epitopes absent in proliferating cells. This represents an early step toward antigen discovery rather than a completed vaccination experiment (Frescas et al., 2017). Yoshida and colleagues developed a CD153 peptide vaccine that eliminated a specific adipose T-cell population in high-fat-diet mice, eliciting sustained antibody production and complement-dependent cytotoxicity, which conferred metabolic benefits. However, it targeted a metabolically relevant T-cell state rather than a conserved senescence antigen (Yoshida et al., 2020). Suda and colleagues identified GPNMB through transcriptomic comparison of senescent endothelial cells and translated this into a peptide vaccine that reduced GPNMB-positive and senescence-associated cells, improved metabolic and vascular phenotypes, and conferred a sex-dependent lifespan benefit in progeroid mice. Subsequent research demonstrated that GPNMB supports lysosomal integrity and is also expressed by macrophage-lineage, osteoclast-related, and injury-responsive cells (Suda et al., 2021; 2022). A CD38 vaccine reduced age-related immunometabolic myeloid expansion and improved physical, cognitive, and metabolic parameters; however, because CD38 has broad physiological functions, the translational challenge is to remove the pathological population while maintaining protective immune functions (Yu et al., 2025; Piedra-Quintero et al., 2020).

Delivery methods can influence whether a nominated antigen elicits a therapeutic response within an aged immune system. Both a GPNMB dendritic-cell vaccine and a self-assembling GPNMB nanovaccine performed better than the corresponding protein vaccine in aged or progeroid mice. Aging also impairs the antigen-presenting pathway on which a senolytic vaccine relies (Cao et al., 2024; Zhang et al., 2025). Active vaccination provides sustained surveillance with a limited number of doses; however, this durability also raises safety concerns, as vaccine-induced memory targeting elements shared with infection-responsive leukocytes, reproductive tissues, or progenitors could cause episodic toxicity long after immunization (Grosse et al., 2020; Zhao et al., 2024).

### 5.2 Engineered cells, antibodies, and lysate platforms

uPAR-directed CAR-T cells recognize an intact surface antigen independently of HLA, reduce senescence-associated cells in oncogene-induced liver senescence and fibrosis, enhance survival in a senescence-inducing lung cancer regimen, and, in subsequent studies, improve metabolic function in aged mice with cellular persistence exceeding 15 months. An intestinal investigation revealed that removing uPAR-positive cells augmented rather than impaired regeneration in the aged gut (Amor et al., 2020; Amor et al., 2024; Eskiocak et al., 2026). This constitutes one of the most internally consistent evidence chains regarding immune-senolytics; however, uPAR is upregulated during injury, inflammation, and remodeling, and the absence of overt toxicity in murine models does not confirm safety during human infection, surgical procedures, or wound healing. NKG2D-ligand-directed CAR-T cells target a family of stress-induced ligands, reduce senescent cells, and improve outcomes in aged mice, while decreasing senescence-associated cells in aged nonhuman primates without observed adverse effects, representing the most advanced cross-species validation in this field. Nevertheless, stress can induce NKG2D ligands rather than restrict them to senescence (Yang et al., 2023; Deng et al., 2024).

Antibody and checkpoint strategies occupy an intermediate, more controlled position: PD-L1-directed depletion and PD-L2 inactivation restore cytotoxic clearance of senescent or therapy-induced senescent cells, and antibody targeting of the GD3 ganglioside checkpoint restores local natural killer function while reducing senescence and fibrosis (Majewska et al., 2024; Iltis et al., 2025). Whole-cell and lysate approaches present a broad antigen repertoire; a dendritic-cell product pulsed with senescent-fibroblast lysate generated polyvalent cytotoxic immunity and antitumor activity but did not identify which antigens mediate the effect, so lysate vaccination is best regarded as a discovery platform to be deconvoluted into a defined panel before human translation (Ichim et al., 2025; Carreno et al., 2015).

### 5.3 Critical appraisal and the telomere-specific gap

Numerous studies have demonstrated immune recognition and the elimination of specific pathological senotypes across various preclinical models. These findings rely primarily on within-study replication and cross-model triangulation rather than independent laboratory validation. While some interventions yield durable functional benefits, the evidence remains predominantly preclinical. Target specificity remains a central unresolved issue, and claims regarding clearance vary in their directness. Specific markers such as CD153 delineate an adipose tissue T-cell state; GPNMB identifies certain, but not all, senotypes; CD38 broadly marks functional immunometabolic cells; uPAR is also induced during injury; NKG2D ligands serve as general stress signals; and GD3 is absent in several oncogene-induced senescent states. None of these markers are universal, and several may be particularly useful because they identify limited subsets. Safety evaluations are less advanced than efficacy assessments and seldom include infection challenges, wound healing, regeneration, or long-term tumor surveillance in aged animals (Yoshida et al., 2020; Suda et al., 2021; Yang et al., 2023; Iltis et al., 2025; Amor et al., 2024a).

Critically, no reviewed intervention specifically targets telomere-dysfunction-associated senescence as an independently validated antigenic state. Existing studies identify antigens from age-associated, metabolic, fibrotic, therapy-induced, replicative, or oncogenic models and then test whether targeting modifies pathology. Whether telomere-dysfunctional cells share GPNMB, uPAR, NKG2D ligands, or a distinct HLA peptidome remains an empirical question requiring direct pairing of telomere-damage measurements with surfaceomic and immunopeptidomic data. This gap defines the bioinformatics opportunity: existing interventions provide candidate antigens, platform lessons, and failure modes, but not a validated telomere-anchored target panel (Rossiello et al., 2022; Sanborn et al., 2025; Goyer et al., 2026).

### 5.4 Transferable lessons from neoantigen cancer vaccines

Personalized cancer vaccination represents the most advanced precedent for computationally guided antigen selection that has been translated into clinical practice. Its trajectory offers valuable insights for developing senolytic antigens. Early studies showed that individually predicted and synthesized neoepitopes could stimulate polyfunctional CD4 and CD8 T cell responses and demonstrate durable clinical efficacy, using both synthetic long peptides and lipoplex mRNA delivery methods. These studies also showed that researchers could produce and administer a defined multi-epitope panel within clinically relevant timelines (Carreno et al., 2015; Ott et al., 2017; Sahin et al., 2017; Kreiter et al., 2015). Subsequent clinical trials in glioblastoma and pancreatic cancer indicated that vaccine-induced T cells can traffic to tumor sites and that responder status correlates with the induction of high-magnitude, persistent T cell clones. This provides a model for the pharmacodynamic endpoints that a senolytic vaccine should specify in advance (Hu et al., 2021; Rojas et al., 2023; Sethna et al., 2025).

Two lessons are particularly compelling. Firstly, prediction functions as a filter rather than a decision-maker: neoantigen programs still depend on eluted-ligand evidence and functional validation because reliance solely on prediction tends to overestimate immunogenicity, reflecting the presentation-versus-immunogenicity gap that is central to this review (Richman et al., 2019; Wells et al., 2020; Purcell et al., 2019). Secondly, the seno-antigen issue is more challenging in a specific and instructive manner. Neoantigens arise from tumor-specific mutations absent in normal tissues, thereby ensuring selectivity, at least in part, through the target’s origin. Conversely, seno-antigens are self-proteins shared with healthy cells; therefore, selectivity must be empirically established by excluding healthy states rather than inferred from non-self-recognition. The cancer vaccine field therefore validates the discovery-to-panel workflow and shows why the healthy-state exclusion criterion cannot be relaxed when targeting senescence (Sahin et al., 2017; Goyer et al., 2026).

The modalities also differ in how they can control risk, which is as important as efficacy. Active vaccination and persistent engineered cells create durable, self-amplifying surveillance that is difficult to withdraw if a healthy-tissue liability emerges, whereas antibodies, transient cellular products, and checkpoint modulation act for a defined interval and can be stopped, dose-adjusted, or reversed. A rational development sequence exploits this difference by using a controllable modality to test the core biological hypothesis, namely, that removing an antigen-positive population produces benefit without unacceptable harm, before committing to a durable format against the same target, and only once the target has satisfied all four compulsory criteria in that modality. Read this way, the existing literature does not identify a finished product or a validated stand-alone target, but supplies candidate targets with route-specific preclinical support, delivery lessons, and, most usefully, a catalog of the failure modes that a controllable first-in-human design must probe (Amor et al., 2024a; Majewska et al., 2024; Iltis et al., 2025; Grosse et al., 2020).

## 6 Data modalities and computational prioritization

The reliability of a prioritization pipeline is constrained by the evidentiary content of its inputs. No single modality can, within a single measurement, confirm that a cell is persistently senescent, that its state is telomere-dysfunctional, that a candidate is surface-exposed or HLA-presented, that it is absent from essential healthy cells, and that an effector can recognize it selectively. Table 6 delineates the question each modality addresses and the directness and primary limitation of its evidence, while Figure 5 displays these modalities as a matched-sample evidence stack that supports a constrained prioritization pipeline (Purcell et al., 2019; Suryadevara et al., 2024; Sanborn et al., 2025).

**TABLE 6:** Grading rubric for the four compulsory criteria and the optional telomere axis. The rubric defines the minimum evidence required for each grade, differentiates between untested results and negative outcomes (not met), and treats unresolved conflicting findings as either context-stratified or lower-certainty grades rather than merging them. It further specifies functional recognition as route-specific, antigen-dependent recognition and senolytic clearance, rather than simply killing. Additionally, it mandates a prespecified, route-specific safety margin to exclude healthy states and identifies critical thresholds. The grading of functional recognition is based on preclinical, route-appropriate, antigen-dependent evidence. Readiness for human application is monitored separately (see the Human-translation column of Table 2), rather than by downgrading the preclinical functional recognition grade, to avoid selectively demanding human evidence for a specific route.

Criterion	Met (minimum evidence)	Partial	Not met	Untested/hard stop
Persistent senescence	Durable proliferative arrest where arrest is biologically assessable, or, in postmitotic lineages, lineage-appropriate evidence of a persistent pathological senescent state, plus ≥2 orthogonal senescence features in the target cells in the disease context, demonstrated at two separated time points or in a chronic human lesion and meeting the co-localization specificity and coverage thresholds stated in Section 7	One orthogonal feature only, arrest of uncertain durability, or a single-marker call	Phenotype attributable to quiescence, differentiation, or another non-senescent state	Untested if multi-feature data are absent; conflicting findings are not merged into one grade but yield a context-stratified or lower-certainty grade
Immune accessibility	Direct detection on intact cells (surface) or eluted HLA ligand with FDR control, demonstrated at the late stabilized senescent state rather than at an early or inducer-stimulated state, with ligand density recorded in calibrated units	Direct total-protein evidence without intact-cell surface localization, or intact-cell detection shown only at an early or stimulated state	Intracellular-only or shed-decoy evidence	mRNA or transcript-only evidence, or no intact-cell surface or eluted-ligand assay performed
Functional recognition	Route-specific antigen-dependent recognition and senolytic clearance in the intended modality (killing, complement, antibody-dependent cellular cytotoxicity, phagocytic clearance, natural-killer, or checkpoint de-repression), confirmed by antigen blocking, knockout, or antigen-negative controls (activity is lost when the antigen is removed), with recognition demonstrated at a ligand density at or below the measured natural density for the route (Section 6.3.2)	Recognition or clearance in the intended modality but without antigen-dependence controls (uncontrolled recognition)	No clearance despite an adequate route-appropriate assay in the intended modality	Untested if not assayed in the intended modality; a positive result from another route (for example, CAR-T for a vaccine) is nomination only and is graded untested
Healthy-state exclusion	A prespecified route-specific safety margin is met across essential, activated, progenitor, reparative, and beneficial-senescent cells: target density below the effector-activation threshold, or no antigen-dependent injury at clinically relevant exposure, shown by a defined assay, applied to every one of the seven compartments of the counter-screening panel in Table 7, at the worst-case donor, time point, and activation state and at the conservative bound of the confidence interval, with at least one *in vivo* challenge	Margin met in some but not all required healthy compartments, or demonstrated only *in vitro*; the grade must state numerically how many of the seven compartments in Table 7 were passed and name those not tested	Antigen-dependent injury, or expression at or above the activation threshold on an indispensable healthy population (HARD STOP)	Untested (default) whenever the prespecified healthy-cell panel and exposure have not been tested; graded untested, not met
Telomere axis (optional)	Direct telomere-damage measurement paired with the antigen in the same cells	Telomere data in a related but not matched sample	Antigen enriched in telomere-intact senescence	Untested (default) whenever matched same-cell data are absent; unrelated models do not establish convergence

Abbreviations: FDR, false discovery rate; FISH, fluorescence *in situ* hybridization; HLA, human leukocyte antigen.

**FIGURE 5:**
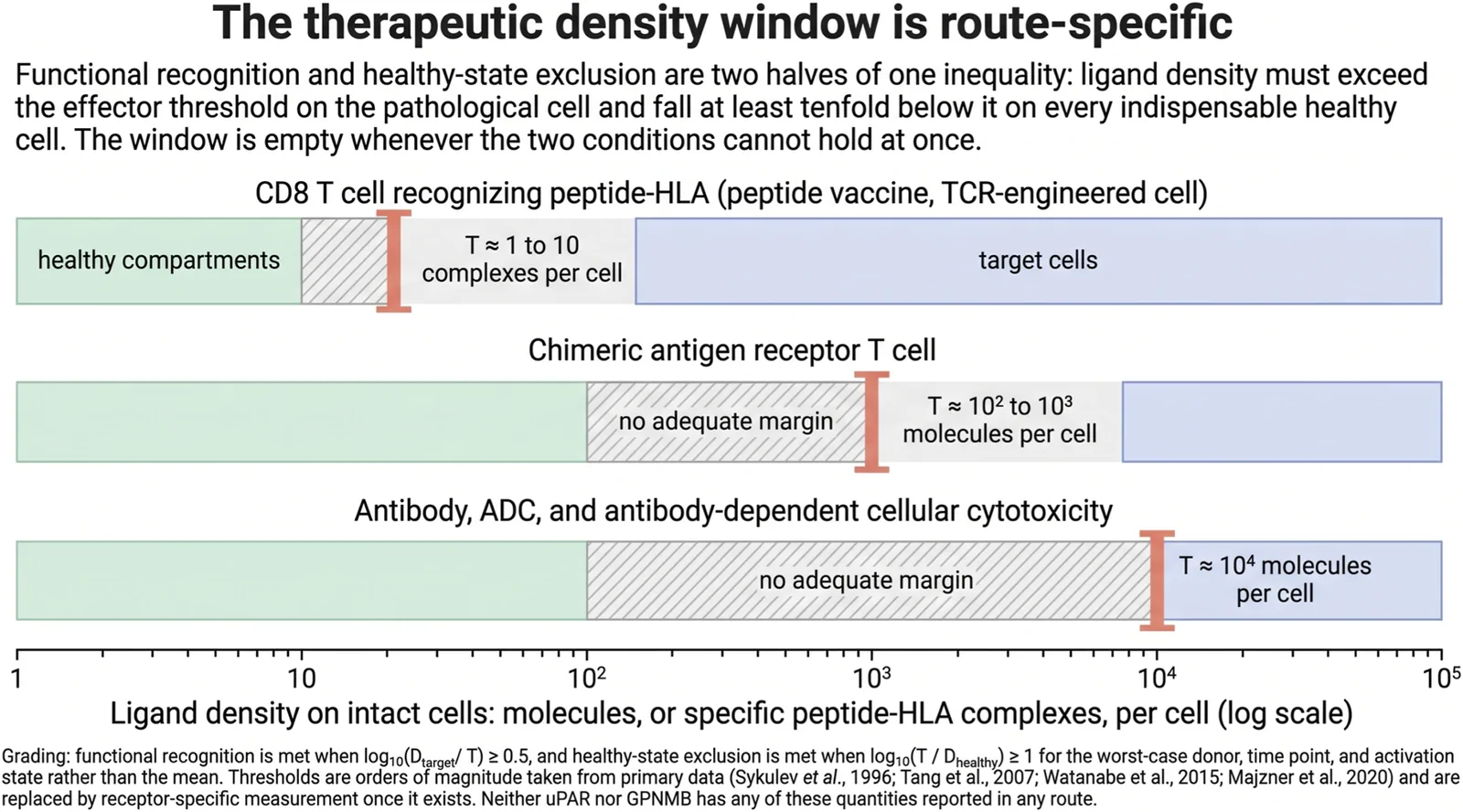
The therapeutic density window is route-specific. Functional recognition and healthy-state exclusion are two-halves of one inequality. Ligand density must exceed the effector threshold on the pathological cell and fall at least tenfold below it on every indispensable healthy cell, evaluated at the worst-case donor, time point, and activation state. Thresholds differ across routes by several orders of magnitude, so a density that is safe for one modality can be dangerous for another. The window can be empty for an antigen that is genuinely senescence-enriched. Abbreviations: ADC, antibody-drug conjugate; CD8, cluster of differentiation 8; D_healthy, ligand density on the healthy cell; D_target, ligand density on the pathological target cell; GPNMB, glycoprotein nonmetastatic melanoma protein B; HLA, human leukocyte antigen; T, effector activation threshold; TCR, T-cell receptor; uPAR, urokinase-type plasminogen activator receptor.

### 6.1 Reference sets, harmonization, and the matched-sample backbone

Here, the discovery process is characterized as a conceptual decision architecture rather than an implemented algorithm. The subsequent description provides a specification of the evidence sequence and the constraints that a future implementation must adhere to; it does not encompass, nor does it claim to include, an objective function with fixed weights, exclusion thresholds, an uncertainty model, executable code, an input dataset, or a prospective benchmark, all of which constitute necessary future work. The fundamental premise is that discovery should be regarded as a constrained decision problem rather than merely searching for genes exhibiting the largest fold changes, because a naive approach that identifies senescent cells, performs differential expression analysis, and selects the most upregulated transcripts fails at multiple stages: messenger RNA abundance alone does not determine protein production, surface localization, natural HLA presentation, immune recognition, or selectivity (Wollscheid et al., 2009; Purcell et al., 2019). Instead, the architecture begins with an explicit target product profile that specifies the biological state, tissue, disease, immune mechanism, intended population, product format, and non-negotiable safety constraints. Subsequently, it defines a positive reference set of the intended pathological senescent cells, optionally stratified by mechanism, and a physiologically diverse negative set (Ogrodnik et al., 2024; Dai and Gifford, 2023).

Constructing a reference set requires donor-aware classification and cross-study meta-analysis, with explicit adjustments for batch effects, class imbalance, and pseudo replication, because treating cells from the same donor as independent replicates can inflate confidence intervals. Harmonization depends on standardized gene and protein identifiers, interoperable metadata, explicit software versioning, and reusable formats aligned with FAIR principles. Additionally, it relies on public resources such as senescence signature libraries and atlases, human protein-expression and surfaceome references, immunopeptidome repositories, and HLA allele-frequency databases, which are utilized for counter-screening rather than solely for discovery purposes (Wilkinson et al., 2016; Sanborn et al., 2025; Suryadevara et al., 2026; Uhlen et al., 2015; Bausch-Fluck et al., 2018).

### 6.2 Two verification routes and hard exclusions

Because surface antigens and HLA peptides are recognized by distinct effector mechanisms and exhibit distinct failure modes, the pipeline delineates two separate verification pathways. The surface-antigen pathway assesses extracellular topology, isoform architecture, target density, shedding, internalization, and detection on intact cells, considering transcript-only evidence as hypothesis-generating and necessitating direct surface validation prior to classifying a differentially expressed gene as an antibody or receptor target (Wollscheid et al., 2009; Bausch-Fluck et al., 2018; Purcell et al., 2019). Conversely, the HLA-peptide pathway evaluates naturally eluted ligands using defined false-discovery-rate controls, allele assignment, and abundance metrics, and uses epitope-prediction models and allele-frequency data to estimate population coverage. This pathway strictly separates presentation from functional immunogenicity (Sarkizova et al., 2020; Wells et al., 2020; Toussaint et al., 2008).

Within this specification, exclusions are strict constraints that precede any comparative evaluation, ensuring that candidates with high accessibility in essential tissues, activated immune cells, progenitors, repair states, or beneficial senescent cells are excluded rather than penalized. Such candidates are permitted to persist based on other advantageous properties. The process of satisfying this constraint is defined operationally rather than through assertion: a candidate is deemed to meet healthy-state exclusion criteria only when a predefined, route-specific safety margin is demonstrated across critical compartments, including activated, progenitor, reparative, and beneficially senescent cells, such as maintaining target density below the effector-activation threshold for the relevant modality, or demonstrating the absence of antigen-dependent injury at clinically relevant exposure levels within a specified assay. If the relevant panel and exposure have not been tested, the criterion is classified as untested rather than met, given that the absence of evidence of harm does not constitute evidence of safety. Only candidates that meet this constraint are then compared across parameters such as directness, donor and cell-type prevalence, accessibility, functional recognition within the intended modality, healthy-tissue findings, missing data, and uncertainty. These comparisons are recorded as an evidence card that links each judgment to its supporting and contradictory studies. The purpose of organizing evidence in this manner is to prevent correlated transcriptomic or predictive evidence from being counted as independent validation and to prioritize subsequent experiments that could most significantly influence a go/no-go decision. This approach intentionally avoids presenting a single numeric ranking, as the weights and thresholds needed to produce one are neither established nor supported by the evidence, thereby avoiding unwarranted precision (Purcell et al., 2019; Dai and Gifford, 2023; Zeng and Gifford, 2019).

#### 6.2.1 Derivation and adequacy of the four criteria

The four criteria are not a list assembled from the literature by inspection. They are the four necessary links in the causal chain between a molecule and a therapeutic effect, and they follow from asking what must hold jointly, in the same cells, for an immune effector to remove a pathological cell because of antigen A. The cell must occupy the state the therapy intends to remove and remain in it long enough to be a target; this is persistence. Antigen A must be physically available to the recognition apparatus, either as an intact extracellular structure or as a peptide occupying an HLA groove, which is accessibility. Engagement of A must be sufficient to trigger the effector mechanism actually deployed, and the clearance must disappear when A is removed, which is functional recognition. Cells whose survival is required must not display A above the activation threshold of that effector, which is healthy-state exclusion. Each is a necessary condition of a recognition event, and failure of any one voids the therapeutic claim irrespective of the strength of the other three, which is why the architecture is non-compensatory.

Each criterion also maps onto a documented and recurrent failure mode, providing empirical rather than deductive justification. Criterion one addresses misclassification of quiescent, differentiated, transiently arrested, and transiently reparative populations as pathological senescence (Gorgoulis et al., 2019; Ogrodnik et al., 2024). Criterion two addresses nomination of transcripts encoding intracellular isoforms, shielded domains, rapidly internalized proteins, or shed decoys (Wollscheid et al., 2009; Bausch-Fluck et al., 2018). Criterion three addresses the presentation-immunogenicity gap, which human senescence data show is the norm rather than a theoretical concern (Purcell et al., 2019; Goyer et al., 2026). Criterion four addresses on-target off-tumor toxicity, the dominant clinical failure mode of self-antigen-directed immunotherapy (Fedorov et al., 2013; Srivastava et al., 2019).

The criteria are necessary but not asserted to be sufficient. Sufficiency for clinical relevance requires quantitative conditions that operate within the criteria rather than alongside them: the ligand density margin between target and healthy compartments, the fraction of the pathological compartment carrying the antigen, the temporal stability of expression, and the permissiveness of the immune microenvironment. These are treated as quantitative modifiers within criteria two, three, and four, as specified in Section 6.2.2, Section 6.3.1, and Section 6.3.2, rather than as additional binary gates, because converting a continuous quantity into a fifth pass-or-fail criterion would reintroduce the arbitrariness the framework is meant to avoid. The criteria set would be shown inadequate if a candidate satisfying all four in its intended modality failed prospectively for a reason not captured by them. The three most plausible such reasons are antigen modulation and internalization kinetics under effector pressure, immunosuppression within the senescent niche, and effector exhaustion in aged hosts. The first fits criterion two as a density measurement over time; the second and third fit criterion three, which requires demonstrating recognition in the relevant microenvironment and host age. Whether that accommodation is adequate is an empirical question, and the study that would answer it is specified in Section 9.2.

#### 6.2.2 A prespecified healthy-state counter-screening panel

Healthy-state exclusion fails as a criterion if it remains a general requirement, so it is specified here as a fixed panel that can be executed, costed, and audited. Table 7 enumerates seven compartments, each with a model system, a readout, a pass condition, and the failure mode it detects. The grading rule is arithmetic rather than descriptive. A met grade requires every compartment to pass. A partial grade must state the number of compartments passed out of seven and name those omitted, so that a qualitative hedge becomes an auditable count. Untested remains the default.

**TABLE 7:** Route-specific evidence requirements and transferability. Each therapeutic route possesses its own recognition mechanism and validation criteria; evidence generated for one route nominates, but does not validate, a target for another.

Route/product	Recognition mechanism	What functional recognition requires	What transfers to other routes
Antibody/ADC	Intact-surface epitope; Fc or payload effector	Intact-cell binding at adequate density; antigen-dependent killing; healthy-cell sparing	Nominates a surface target for engineered cells; does not establish vaccine tolerance or T-cell immunity
Engineered cell (CAR/logic-gated)	Receptor-defined surface antigen; HLA-independent; AND-gating possible	Receptor-dependent lysis with antigen-negative controls; on-target off-tumor mapping	Nominates the surface antigen; does not establish natural HLA presentation, tolerance, or vaccine immunogenicity
Native-surface-antigen vaccine	Host antibody and T-cell response to a self surface protein	Overcome tolerance; effective titer or T-cell response; antigen-dependent clearance; healthy sparing	Confirms accessibility of the antigen; does not transfer CAR killing data as vaccine efficacy
HLA-restricted peptide vaccine	CD8/CD4 recognition of naturally presented peptides	Eluted human ligands; population coverage; demonstrated human immunogenicity and cytotoxicity	Murine or predicted data nominate; human presentation and immunogenicity remain required

Abbreviations: ADC, antibody-drug conjugate; AND, boolean conjunction operator denoting engagement only when both specified inputs are present on the same cell; CAR, chimeric antigen receptor; CD4, cluster of differentiation 4; CD8, cluster of differentiation 8; Fc, fragment crystallizable region of an immunoglobulin; HLA, human leukocyte antigen.

Two design principles govern the panel. Expression data alone cannot establish functional sparing, so the panel pairs density measurement with functional challenge: a compartment in which the antigen is present but below the effector threshold passes on density, while a compartment in which density cannot be measured must pass on a functional challenge with an antigen-dependence control, because an unimpaired endpoint in the absence of such a control may reflect nothing more than inadequate effector exposure. The summary statistic is the worst case rather than the average, so the reported quantity is the highest density observed across donors, time points, and activation states, evaluated at the conservative bound of its confidence interval, since a therapy encounters the worst-case cell rather than the mean cell. The temporal structure of the panel matters for the same reason. Injury-responsive and activated compartments are sampled across a time course rather than at a single point, because an antigen may be absent at baseline and induced transiently, which is the pattern a resting-tissue expression atlas cannot detect and which has repeatedly produced clinical surprises in self-antigen-directed immunotherapy (Srivastava et al., 2019).

### 6.3 Explicit grading and the separation of nomination from validation

Because the framework’s outputs are qualitative judgments, the grades must be defined rather than asserted. Table 1 specifies, for each criterion, the minimum evidence required for a grade of met, the conditions that yield partial, what constitutes not met, and the distinct grade of untested, which applies when the necessary measurement has not been made in the relevant cells and which is not the same as negative evidence. The rubric also states how it resolves conflicting studies, records missing data as untested rather than as a pass or a fail, and identifies findings that act as hard stops. Applying this rubric requires the evidence card to name every supporting and contradictory study and the exact finding used for each judgment, which is why the appraisal in Table 3 records model, controls, and limitations at the study level (Purcell et al., 2019; Goyer et al., 2026).

The framework further distinguishes target nomination from product validation, as the four criteria must be met within the modality used. Table 8 outlines route-specific requirements for antibody, engineered-cell, native-surface-antigen, and HLA-restricted peptide vaccine products and specifies evidence transferability among them. Recognition of an intact surface antigen by a chimeric antigen receptor confirms the antigen for the engineered-cell pathway but only nominates it for a vaccine, since a receptor circumvents the tolerance, affinity maturation, and natural processing prerequisites that a vaccine must satisfy. Similarly, murine peptide-vaccine activity nominates but does not validate a human peptide vaccine, which additionally requires naturally presented human ligands, coverage across pertinent alleles, and demonstrable human immunogenicity. Evidence obtained from one pathway may thus support nomination but must never be regarded as validation of another (Amor et al., 2020; Purcell et al., 2019; Sarkizova et al., 2020).

**TABLE 8:** Ordinal scoring scheme with anchored levels and a non-compensatory decision rule. Untested is recorded separately from a score of 0 and can never yield a pass. A candidate advances only when the minimum score across the four compulsory criteria is 3 within the intended modality; a hard stop terminates the candidate regardless of the remaining scores.

Criterion	Score 0, not met	Score 1 to 2, partial	Score 3, met	Measurement that upgrades the score	Evidence that may not be reused for another criterion
Persistent pathological senescence	Phenotype attributable to quiescence, differentiation, or transient checkpoint arrest; hard stop if reversible on inducer withdrawal	1, one orthogonal feature only, or arrest of undocumented durability; 2, two features at a single time point in an acute model	Two or more orthogonal features in the target cells in the disease context, at two separated time points or in chronic human lesion, meeting the co-localization thresholds in Section 7	Multiplexed same-cell imaging of two or more senescence features with a proliferation marker, in human lesional tissue with an age-matched non-diseased comparator	A transcript-level senescence score may support this criterion or accessibility, not both
Immune accessibility	Intracellular-only localization, or evidence attributable to shed antigen or extracellular vesicles	1, total-protein evidence without intact-cell localization; 2, intact-cell detection shown only at an early or inducer-stimulated state	Direct detection on intact cells at the late stabilized state, or eluted HLA ligand with false-discovery-rate control, allele assignment, and copies per cell, with ligand density recorded in calibrated units for the density-window assessment	Quantitative flow cytometry in antibody binding capacity units on intact cells, or targeted immunopeptidomics with an isotope-labeled standard	Surfaceome prediction and transcript abundance may neither substitute for nor be added to direct detection
Functional recognition	No clearance in an adequate route-appropriate assay; hard stop if clearance persists in antigen-negative controls	1, recognition without clearance; 2, clearance without antigen-dependence controls	Route-specific antigen-dependent recognition and senolytic clearance, lost on antigen blocking, knockout, or antigen-negative target, at a ligand density at or below the measured natural density	Route-appropriate cytotoxicity or clearance assay with paired antigen-negative and blocked conditions, on naturally presenting rather than antigen-loaded targets	Predicted immunogenicity may never contribute; evidence from another route contributes to nomination only
Healthy-state exclusion	Antigen-dependent injury, or density at or above the effector activation threshold on an indispensable population (hard stop)	1 to 2, margin demonstrated in some but not all compartments; the number of compartments passed out of seven must be stated numerically	Prespecified margin met in every compartment of Table 7 panel, at the worst-case donor and time point and at the conservative bound of the confidence interval, with at least one *in vivo* challenge	Quantitative density measurement across the counter-screening panel, plus functional challenge with an antigen-dependence control	Absence of reported toxicity in an unrelated study may never be used and is recorded as untested
Telomere axis, optional stratifier and not scored toward the four	Antigen enriched in telomere-intact senescence	Telomere data in a related but unmatched sample	Direct telomere-damage measurement paired with the antigen in the same cells	Telomere-associated DNA damage foci by immuno-FISH on the same section as antigen detection, or single-telomere length analysis on the sorted population	Mean telomere length may not be used as evidence of telomere dysfunction

Abbreviations: FISH, fluorescence *in situ* hybridization; immuno-FISH, combined immunofluorescence and fluorescence *in situ* hybridization.

#### 6.3.1 Ordinal scoring and the non-compensatory decision rule

The rubric in Table 1 defines grades; Table 9 converts them into a scoring scheme that a future implementation can apply mechanically. The scheme is deliberately ordinal and non-compensatory rather than a weighted numeric score. A weighted sum lets strong evidence on one axis offset failure on another, so a candidate with outstanding functional recognition and unacceptable expression on hematopoietic progenitors would outrank a candidate that is merely adequate everywhere, inverting the safety logic the framework enforces. The appropriate formal object for a decision of this shape is an ordinal grading with a lexicographic rule, which is the structure used in evidence appraisal generally (Guyatt et al., 2008).

**TABLE 9:** Route-specific activation thresholds and the quantitative variables that determine whether a given ligand density yields functional recognition. Thresholds are orders of magnitude taken from the cited primary literature and are intended to be replaced by candidate-specific and receptor-specific measurements.

Route and effector	Approximate activation threshold	Governing quantitative variables	Assay for density on intact cells	Prespecified margin for a met grade
CD8 T cell recognizing peptide-HLA (peptide vaccine, TCR-engineered cell)	One to ten specific complexes per cell for cytolysis (Sykulev et al., 1996)	Copies of the specific peptide-HLA per cell; total HLA class I surface level; receptor avidity capped by thymic selection; functional avidity as peptide-titration EC50	Targeted immunopeptidomics with an isotope-labeled synthetic standard; multimer-calibrated functional titration	Target above threshold; every indispensable compartment at least tenfold below, including compartments under interferon exposure
Chimeric antigen receptor T cell	Approximately 10 squared to 10 cubed molecules per cell, tunable by receptor design (Watanabe et al., 2015; Majzner et al., 2020)	Surface molecules per cell; receptor density; hinge and transmembrane composition; activation motif number; a separate and higher threshold for cytokine release	Quantitative flow cytometry in antibody binding capacity units on intact viable cells	Target at least threefold above threshold; every indispensable compartment at least tenfold below at every sampled time point after injury
Antibody, antibody-drug conjugate, and antibody-dependent cellular cytotoxicity	Approximately 10 to the fourth molecules per cell and above, payload dependent (Tang et al., 2007)	Surface molecules per cell; immunoglobulin affinity and avidity; Fc receptor polymorphism; internalization rate; shed antigen acting as a sink	Quantitative flow cytometry, with shed antigen quantified in the relevant fluid	Target above threshold with shed antigen quantified; indispensable compartments at least tenfold below
Natural killer cell engagement of induced ligands	Set by the balance of activating and inhibitory ligands rather than by a single density	Activating ligand density; HLA-E and class I inhibitory ligand density; the ratio rather than either term alone	Paired quantitative flow cytometry of activating and inhibitory ligands on the same cells	A ratio-based margin, with the inhibitory ligand quantified alongside the activating ligand in target and healthy compartments
Native-surface-antigen vaccine eliciting host antibody	As for antibody routes, with the additional requirement of tolerance escape	All antibody-route variables, plus the titer and affinity actually achieved in the host, which cannot be set by design	Quantitative flow cytometry, plus measurement of elicited titer and affinity in the treated host	The antibody-route margin, evaluated at the highest titer and affinity achieved rather than at the mean

Abbreviations: CAR, chimeric antigen receptor; CD8, cluster of differentiation 8; EC50, half-maximal effective concentration; HLA, human leukocyte antigen; HLA-E, human leukocyte antigen E; TCR, T-cell receptor.

The scheme has three components. The ordinal scale itself carries anchored descriptors and, for each level, the minimum measurement that earns it. The decision rule is lexicographic: a candidate advances only when the minimum score across the four criteria equals 3 within the intended modality; any criterion graded untested yields the verdict nomination pending a named measurement; any hard stop terminates the candidate irrespective of every other score. The ranking rule for candidates that pass reports a vector rather than a scalar, comprising the density margin defined in Section 6.3.2, the fraction of the pathological compartment covered, the directness of the evidence, and the prevalence of the finding across donors and lesions. Candidates are ordered on that vector by dominance, and where no candidate dominates, the framework reports a tie and names the discriminating experiment rather than breaking the tie with weights it cannot justify.

Independence between criteria is enforced by an evidence-provenance rule. Two criterion judgments may not rest on the same measurement. Transcript enrichment may support criterion one or criterion two but never both, because a single differential expression result cannot independently establish that a cell is senescent and that a protein is surface-exposed. Predicted binding may never support criterion three under any circumstance. Evidence from one therapeutic route may support nomination but never grading in another, which is the transferability rule stated in Table 8. The evidence card carries a provenance column recording which measurement supports which grade, so that correlated evidence counted twice becomes visible on the face of the card. Every measurement named in the upgrade column of Table 9 is an assay in routine use; what the scheme requires is not new methodology but that the assays be performed in the same cells and reported in comparable units.

#### 6.3.2 The quantitative density window

Functional recognition is not a property of an antigen. It results from an inequality among three quantities: the effective ligand density on the pathological cell, the activation threshold of the deployed effector mechanism, and the ligand density on every cell whose survival is required. Let D denote ligand density in molecules or specific peptide-HLA complexes per cell, measured on intact cells by a calibrated assay, and let T denote the activation threshold of the deployed effector in the same units. Functional recognition is graded met only when the base-ten logarithm of the ratio of D on target cells to T is at least 0.5, and healthy-state exclusion is graded met only when the base-ten logarithm of the ratio of T to the highest D observed on any indispensable healthy population is at least 1. The two conditions define a therapeutic density window, illustrated in Figure 6, whose width rather than the mere presence of the antigen determines whether a candidate is viable. The window can be empty for an antigen that is genuinely senescence-enriched, which is the quantitative content of the claim that enrichment is not selectivity.

**FIGURE 6:**
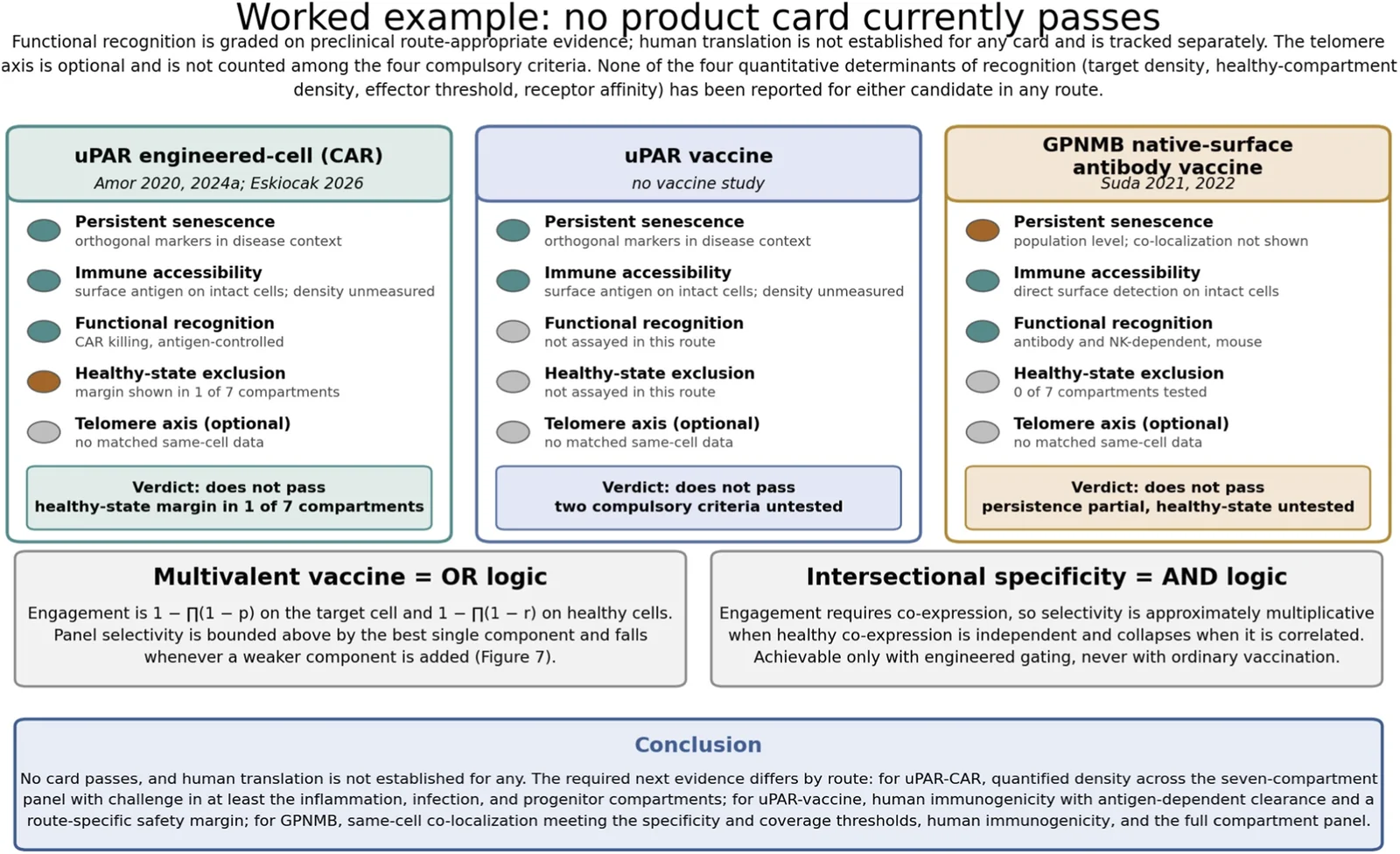
This illustrative example demonstrates that, to date, no evaluated product card has met all established criteria. The assessment encompasses uPAR for its engineered-cell (CAR) and vaccine products, as well as GPNMB for its native-surface antibody vaccine, in accordance with four mandatory standards. Functional recognition is evaluated based on preclinical, route-appropriate evidence. To date, none of the evaluated cards has achieved human translation and is therefore presented separately. Additionally, the telomere axis remains unexplored for each. No card has satisfied all the criteria; moreover, no other reviewed candidate has demonstrated the capacity to meet these four essential standards. The lower panels compare conventional multivalent vaccination, which employs OR logic and combines component liabilities, with engineered AND-gated recognition, the sole pathway to intersectional specificity. This analysis delineates the specific route-based experiments required for each product. The card grades now record the healthy-state criterion as a count of compartments passed out of the seven specified in Table 7, rather than as an undifferentiated partial, and note that none of the four quantitative determinants of recognition defined in Section 6.3.2 has been reported for either candidate in any route. Abbreviations: AND, Boolean conjunction operator; CAR, chimeric antigen receptor; GPNMB, glycoprotein nonmetastatic melanoma protein B; NK, natural killer; OR, Boolean disjunction operator; p, per-component probability of engagement on the target cell; r, per-component probability of engagement on a healthy cell; uPAR, urokinase-type plasminogen activator receptor. AND and OR are capitalized because they name Boolean logic operations and not for emphasis.

Antigen amount and HLA peptide density both enter as D, and they must be measured as copies per cell on intact cells rather than inferred from transcript abundance, total protein, or spectral counts, none of which is proportional to surface availability. Effector activation level enters as T, and T differs across routes by several orders of magnitude, which is the most important quantitative fact for this framework and the reason that route-specific grading is not a formality. T-cell receptor engagement of a small number of specific peptide-HLA complexes suffices for cytolysis, with as few as one to three complexes per target cell capable of triggering a response (Sykulev et al., 1996). Chimeric antigen receptor activity has a threshold in the range of hundreds to thousands of molecules per cell, varies with receptor design, hinge and transmembrane composition, and the number of immunoreceptor tyrosine-based activation motifs, and is higher for cytokine release than for cytolysis (Watanabe et al., 2015; Majzner et al., 2020). Antibody-dependent cellular cytotoxicity still requires higher surface densities and is also governed by immunoglobulin affinity and Fc receptor engagement (Tang et al., 2007). A candidate safely below threshold for a peptide vaccine may be far above it for a chimeric antigen receptor, and the reverse is equally possible. Table 10 records these thresholds with the assay appropriate to each route.

**TABLE 10:** Worked-example evidence cards, one per candidate and intended route. Grading unit: functional recognition is graded on preclinical, route-appropriate antigen-dependent evidence with controls; readiness for human use is recorded separately in the Human-translation column, so that human evidence is not required selectively for one route while preclinical evidence is accepted for another. uPAR is graded separately for its two products (engineered-cell CAR and vaccine). Grades: + met; ∼ partial or context-dependent; − not met; UT untested; the telomere axis is optional and is not counted among the four compulsory criteria. uPAR and GPNMB were selected based on objective criteria: documented antigen-dependent clearance, distinct therapeutic routes, and available primary safety-relevant evidence.

Candidate; route	Persistent senescence	Immune accessibility	Functional recognition	Healthy-state exclusion	Human translation	Telomere	Verdict	Required next evidence
uPAR; engineered-cell (CAR)	+	+	+ (controlled mouse CAR killing)	∼ (margin in 1 of 7 compartments; density unmeasured in all 7)	Not established	UT	Does not pass (healthy-state margin shown in 1 of 7 compartments)	Quantified antigen density across the seven-compartment panel of Table 7 with a post-injury time course; functional challenge in at least the inflammation, infection, and progenitor compartments; human evidence; matched telomere pairing
uPAR; vaccine	+	+	UT (no vaccine assay, even preclinical)	UT	Not established	UT	Does not pass (two criteria untested)	Preclinical then human vaccine immunogenicity and antigen-dependent clearance; a route-specific safety margin across Table 7 panel; telomere pairing
GPNMB; native-surface antibody vaccine	∼ (population and tissue level; same-cell co-localization not shown)	+	+ (controlled mouse antibody/NK-ADCC)	UT (0 of 7 compartments tested)	Not established	UT	Does not pass (persistent partial; healthy-state untested)	Same-cell co-localization meeting the specificity and coverage thresholds in Section 7; human immunogenicity and ADCC; the full seven-compartment panel; telomere pairing

Abbreviations and symbols: ADCC, antibody-dependent cellular cytotoxicity; CAR, chimeric antigen receptor; GPNMB, glycoprotein nonmetastatic melanoma protein B; NK, natural killer; uPAR, urokinase-type plasminogen activator receptor; UT, untested; +, criterion met; ∼, criterion partially met.

Binding affinity enters as the term that converts density into occupancy and therefore into effective rather than nominal density. For antibody and receptor routes, affinity and density trade off within limits, because synapse geometry and receptor downmodulation impose a ceiling beyond which higher affinity no longer lowers the density threshold and can worsen selectivity by enabling engagement of low-density healthy cells. For the peptide route, the constraint differs in kind and is more restrictive, because the affinity of the available T-cell receptor repertoire against a self peptide is capped by thymic selection, so a naturally presented senescence-associated self peptide is typically recognized only by low-avidity receptors, as the autoimmune literature has shown directly for beta cell antigens (Bulek et al., 2012). Peptide-HLA binding affinity, which prediction tools estimate, is therefore the wrong quantity to optimize peptide affinity for the HLA molecule governs presentation, while receptor affinity for the complex governs recognition, and only the latter determines whether a presented peptide is functionally visible.

The evidence card records D on target cells, D on the worst-case healthy compartment, T for the intended route as specified in Table 10, and receptor or antibody affinity where relevant, and marks each as measured or missing. None of these four quantities has been reported in the published literature for uPAR or GPNMB in any route, as recorded in the worked example in Section 7.

### 6.4 Combination as a conditional, downstream decision

When several candidates each meet the four criteria within the intended modality, their combination constitutes a valid design question. However, the combination is not simply the sum of individual scores; components may duplicate coverage of the same senotype or allele, share a regulatory pathway and fail collectively, compete during processing, or contribute cumulatively to healthy-tissue risk. For OR-logic vaccination, the combined product preserves each component’s individual liabilities; this supports vaccine design that incorporates panel size, allele frequency, population coverage, diversity, and probabilistic presentation as objectives and constraints, serving as appropriate formal tools once qualified components are available. The seno-antigen problem would extend these frameworks to include cell-lineage coverage, surface accessibility, exclusion of beneficial senescence, and modality-specific safety measures. Conversely, intersectional specificity is not a property of panel composition but rather a recognition-logic attribute derived solely from engineered AND-gated systems, and it is outside the scope of conventional vaccination strategies. Combination is therefore treated as a conditional, downstream design decision rather than a mechanism to salvage candidates that fail individual criteria (Toussaint et al., 2008; Liu et al., 2020; 2024; Dai and Gifford, 2023).

### 6.5 Single-cell and spatial analysis without false confidence

Single-cell and spatial data are essential for identifying rare senescent cell populations and their immune environment; however, they introduce statistical challenges that, if not properly addressed, can lead to highly confident yet irreproducible candidate lists. The primary concern is pseudoreplication: treating thousands of cells from a limited number of donors as independent samples artificially inflates statistical significance and yields differential expression findings that lack generalizability. Before declaring a gene enriched for senescence, researchers should use donor-aware analysis methods, such as pseudo-bulk aggregation or mixed models, rather than naive per-cell testing. Benchmark studies demonstrate that this methodological choice significantly influences which candidates are ultimately identified (Squair et al., 2021; Zimmerman et al., 2021; Murphy and Skene, 2022).

Integration and annotation add further risk. Batch-correction and dataset-integration methods can remove genuine biological signal along with technical variation, and over-integration can merge distinct senescent states, so integration should be benchmarked and its effect on the target population inspected rather than assumed benign (Luecken et al., 2022; Hao et al., 2021). Ambient RNA, doublets, and dropout can create spurious co-expression, and spatial platforms add segmentation error that can assign a neighbor’s transcripts to a senescent cell. The framework therefore treats single-cell nomination as hypothesis-generating and requires a candidate to survive donor-level statistics, integration sensitivity analysis, and orthogonal protein confirmation before advancing to the surface or HLA routes (Squair et al., 2021; Luecken et al., 2022).

### 6.6 Immunopeptidomics and epitope prediction in practice

The HLA-peptide pathway depends on the quality of eluted-ligand data and the controlled application of prediction methods. Mass spectrometry immunopeptidomics captures naturally presented ligands; however, identification rates, false discovery rate management, and allele deconvolution depend on factors such as the instrument used, sample quantity, and the analysis pipeline. Spectral intensity prediction has increasingly become significantly important for improving the identification of non-tryptic HLA peptides. Consequently, validate candidate peptides with defined false discovery thresholds, accurate allele assignment, and, where feasible, targeted reacquisition rather than relying solely on a single discovery run. Additionally, deposit data in community repositories to facilitate reanalysis (Purcell et al., 2019; Wilhelm et al., 2021; Adams et al., 2024; Perez-Riverol et al., 2025).

Predictors of binding and presentation, such as pan-allele eluted-ligand models, serve as valuable tools for prioritization and estimating population coverage. However, these models are trained on data that sample alleles unevenly and are incapable of directly determining immunogenicity. The performance of these predictors diminishes for alleles that are underrepresented in training datasets, alleles that are often more prevalent in populations that are typically underrepresented. Consequently, predictions should include allele-specific confidence levels and be validated against eluted-ligand evidence rather than treated as definitive (Reynisson et al., 2020; O'Donnell et al., 2020; Gfeller et al., 2023; Atkins et al., 2024). Curated resources containing experimentally validated epitopes and HLA ligands provide critical support for counter-screening against known self- and pathogen-derived peptides, which is essential for anticipating cross-reactivity before functional testing.

#### 6.6.1 Operational tests for tolerance escape

Because presentation is not immunogenicity, a framework that grades the peptide route must specify how a tolerized self peptide is distinguished from a functionally immunogenic one. Five ordered tests are proposed, each with an explicit pass condition, and the sequence matters because each test is informative only if the preceding one has passed.

The first test confirms presentation. The peptide must be recovered as a naturally eluted ligand at a false discovery rate below one percent, with unambiguous allele assignment, and with targeted reacquisition against a heavy isotope-labeled synthetic standard that also yields copies per cell. Discovery-run identification without targeted confirmation is insufficient, and copies per cell is required because it sets the density at which every subsequent functional test must be conducted. The second test establishes availability of the repertoire. Peptide-HLA multimer enrichment from the naive CD8 compartment of HLA-matched healthy donors determines whether precursors exist at all, and human naive precursor frequencies for foreign and self epitopes fall within a conserved range of roughly one per hundred thousand to one per million CD8 cells (Alanio et al., 2010), so a frequency indistinguishable from background after enrichment in multiple donors is the operational signature of clonal deletion. This test is necessary but weak, since surviving low-avidity precursors are common.

The third test is decisive. Naive precursors from at least three HLA-matched healthy donors are primed *in vitro* without artificial enhancement, and the resulting T cells must recognize and kill naturally presenting senescent cells rather than peptide-pulsed targets. Recognition that occurs only against exogenously loaded targets is not evidence of immunogenicity, because pulsing routinely raises surface density orders of magnitude above natural presentation and therefore tests the receptor rather than the antigen. A peptide recognized when loaded but not when naturally presented is, operationally, a tolerized peptide, and this single comparison separates the two categories more reliably than any repertoire or predictive measure. The fourth test quantifies functional avidity as the peptide concentration producing half-maximal killing, with the requirement that killing occur at or below the copies per cell measured in the first test, which converts the question from whether recognition is possible into whether it is possible at the density the disease actually presents.

The fifth test places the result in its tolerance context. Thymic expression of the source gene in medullary thymic epithelium, and its dependence on promiscuous autoimmune-regulator-driven expression, predict the depth of central tolerance, and the balance between conventional and regulatory T-cell responses in the priming culture predicts the peripheral outcome. A peptide whose source protein is abundantly expressed in thymic epithelium and which elicits only low-avidity or regulatory-dominated responses is graded as tolerized even when some recognition is detectable. This sequence applies with full force to senescence-associated peptides because they derive from proteins expressed in healthy tissue, placing them at the unfavorable end of the tolerance spectrum relative to tumor neoantigens. No reviewed senescence candidate has undergone the third and fourth tests in human cells, so this review grades criterion three as untested for every peptide-route candidate (Purcell et al., 2019; Goyer et al., 2026).

### 6.7 Uncertainty quantification and reproducible reporting

Because each stage of the process includes measurement error, incomplete coverage, and model uncertainty, future implementations should propagate these uncertainties rather than dismiss them. Deterministic point rankings suggest a level of precision the data do not support and may foster overcommitment to a single candidate; therefore, the current framework provides graded criteria instead of a definitive numeric score. A quantitative approach would require calibrated probabilistic outputs, explicit modeling of missing evidence, and detailed reporting of alleles, tissues, and cell types lacking evidence, thereby enabling differentiation between candidates that have been tested and passed versus those that have merely been untested. These are prerequisites for future developments, not capabilities asserted presently. Even in its qualitative form, the framework identifies the experiment whose results would most significantly reduce decision uncertainty, fulfilling the evidence card’s practical intent (Zeng and Gifford, 2019; Dai and Gifford, 2023). Ensuring reproducibility means documenting inputs, versions, parameters, and outputs in line with FAIR principles, so any future candidate list can be regenerated, audited, and prospectively benchmarked against experimental data as more information becomes available (Wilkinson et al., 2016; Perez-Riverol et al., 2025).

### 6.8 Integrating direct mechanistic phenotyping into discovery

When a target product profile selects a mechanistic stratification, that mechanism requires a specific, often unmet data requirement: it must be measured directly in the same samples, ideally in the same cells, from which candidate antigens are identified. Telomere dysfunction is the clearest example, as most senescence discovery datasets categorize cells using transcriptomic or marker-based scores that do not indicate telomeric damage. Consequently, a candidate can be labeled senescence-associated without evidence that the population exhibits telomere dysfunction rather than being induced by oncogene activity, therapy, or inflammation. For a product stratified by telomere status, addressing this gap requires combining single-cell or spatial expression data with a direct dysfunction readout, such as telomere-associated DNA-damage foci, shortest-end assays, or telomeric damage markers. Conversely, if a product is stratified by another mechanism, measure the corresponding oncogenic, mitochondrial, inflammatory, or therapy-induced signature within the same cells. This approach ensures that the positive reference set reflects the specific mechanism rather than a general state score (Hewitt et al., 2012; Rossiello et al., 2022; Suryadevara et al., 2024).

Emerging multimodal and imaging methods are making this integration increasingly feasible. Dedicated human senescence atlases are increasingly standardizing the definition and dissemination of senescent states across laboratories. The framework considers a candidate mechanistically stratified only when three interconnected observations appear in matched data: the population directly supports the selected mechanism, it meets a multi-feature persistent-senescence definition, and the candidate antigen is enriched in that specific population rather than in the broader senescence-associated compartment. In cases where matched data are not yet available, the mechanistic axis is classified as untested rather than presumed to be satisfied, explicitly distinguishing the recent example below for uPAR and GPNMB, whose telomere axis remains unverified (Sanborn et al., 2025; Suryadevara et al., 2026; Barnes et al., 2022).

#### 6.8.1 A matched-measurement study design

The requirement for matched data is meaningless without a design that can deliver it. The following four-layer design applies to human diseased tissue with age-matched non-diseased comparators, with at least six donors per group and the donor rather than the cell as the unit of analysis.

Layer one is same-section multiplexed imaging followed by telomere immuno-FISH. Imaging mass cytometry or iterative immunofluorescence measures the candidate antigen, two or more orthogonal senescence features, a proliferation marker, and lineage markers with subcellular resolution (Giesen et al., 2014; Gut et al., 2018), and the same section is then processed for telomere-associated DNA damage foci. This layer is the backbone of the design rather than a supporting measurement, because it is the only one that delivers mechanism, phenotype, and antigen in the same cell. Layer two is index-sorted single-cell sequencing of dissociated tissue with antibody-derived tags for the candidate surface antigen and HLA class I (Stoeckius et al., 2017), linking surface protein to transcriptional state in the same cell and allowing imaging-derived gates to map onto transcriptional definitions comparable across studies.

Layer three is immunopeptidomics on the sorted target population, and here the design meets a limitation that should be stated rather than designed around. Mass-spectrometry-based elution of HLA ligands presently requires on the order of ten million or more cells, so matched immunopeptidomics at single-cell resolution is not achievable with current technology. The design therefore specifies population-level immunopeptidomics on cells sorted using gates derived from layers one and two, and the resulting claim is presentation within a phenotypically and mechanistically defined stratum rather than presentation by individually characterized cells. This is the weakest link in the chain and the main technical barrier to fully implementing the framework. Layer four is function: the sorted target population and each healthy comparator population from Table 7 panel are placed in co-culture with autologous or HLA-matched effectors appropriate to the intended route, with antigen blocking, knockout, and antigen-negative conditions run in parallel, so that criteria three and four are evaluated on the same cells whose mechanism and phenotype were established above.

The primary endpoint is the proportion of antigen-positive cells that are both telomere-damage positive and multi-marker senescent, and the variance governing sample size is between-donor rather than between-cell. Six donors per group is adequate to detect a difference of approximately thirty percentage points in that proportion at eighty percent power under inter-donor variability typical of human lesional imaging studies, an illustrative calculation that requires pilot variance estimates before it is treated as a definitive sample size. Where fresh human tissue is unavailable, substitution proceeds in a fixed order, from primary human cells of the relevant lineage with a defined inducer, to induced pluripotent stem cell-derived models, to animal models, and the grade is capped at partial for any layer executed in a substituted system, because the framework requires matched data in the disease context.

## 7 Worked example: applying the framework to reviewed candidates

To demonstrate the criteria’s practical application, two reviewed candidates, uPAR and GPNMB, were evaluated in their intended modalities using only published evidence, in accordance with the explicit rubric in Table 1. These candidates were selected based on objective criteria rather than informal prominence: each has documented antigen-dependent clearance of senescent cells; they represent distinct therapeutic approaches (an engineered-cell product and a native-surface vaccine); and both have primary safety-relevant evidence available for assessment. Since all four mandatory criteria must be evaluated within the intended modality, uPAR was graded separately for its two products, the engineered-cell CAR and a prospective vaccine. The grades reflect preclinical validation appropriate to each route: functional recognition is deemed achieved when controlled preclinical studies demonstrate route-specific antigen-dependent clearance, and preparedness for human application is documented separately as a human-translation status, rather than by downgrading the preclinical grade, ensuring that human evidence is not selectively required for a single route. The graded evaluations are depicted in Figure 7 and summarized in Table 2. This exercise is illustrative rather than definitive, as a comprehensive evaluation would require matched-sample data on telomeres, surfaceome, immunopeptidome, and human immunogenicity, data the field has not yet fully developed. Its purpose is to illustrate that the framework produces an auditable card with an explicit verdict and clearly delineated subsequent evidence, and that, based on current evidence, no card passes the evaluation (Amor et al., 2020; Amor et al., 2024; Suda et al., 2021; 2022; Eskiocak et al., 2026; Cao et al., 2024). This selection rule intentionally and conservatively prioritizes the best-evidenced candidates. Applying a stringent framework to weakly evidenced candidates and finding that they fail demonstrates nothing, since their failure is already implied by the absence of evidence, whereas applying it to the two candidates with the most complete published evidence establishes an upper bound on the readiness of the field: if the strongest available candidates do not pass, no candidate with less evidence can be assumed to pass. The converse inference is not available, and the limitation of the exercise is that two failing cards cannot demonstrate how the framework behaves when a candidate passes, which only the prospective study in Section 9.2 can show.

**FIGURE 7:**
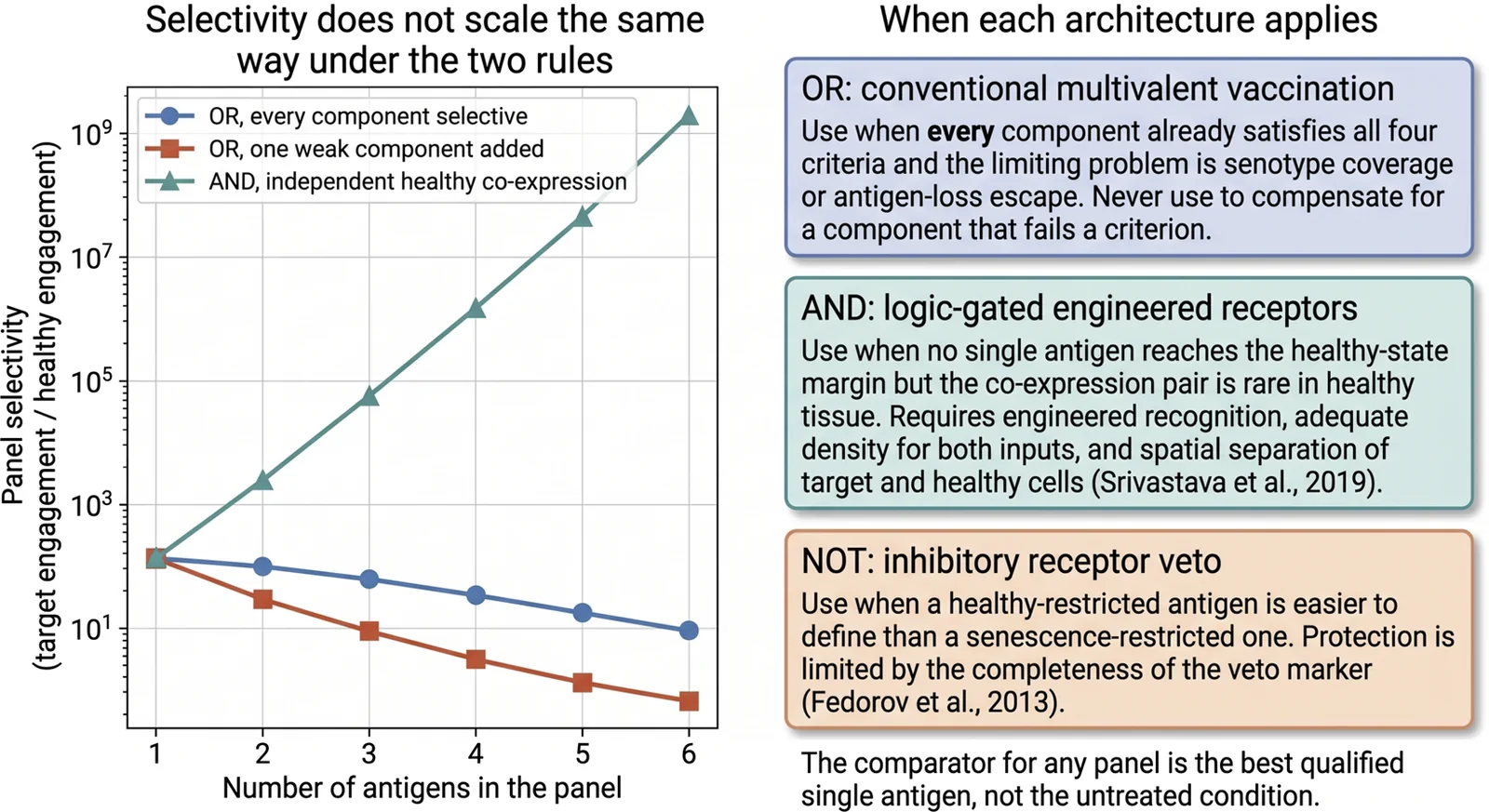
Recognition architectures do not scale the same way. Under OR logic, panel selectivity is bounded above by the best single component and falls when a weaker component is added, so a panel inherits the union of component liabilities. Under AND logic, selectivity is approximately multiplicative when healthy co-expression is independent, and the curve shown assumes both that independence and reliable co-expression on target cells; practical gates use two inputs, and correlated healthy co-expression collapses the gain toward the better single antigen. The panel on the right states the conditions under which each architecture, including inhibitory NOT gating, is the appropriate choice. Abbreviations: AND, Boolean conjunction operator, engagement only when both inputs are present on the same cell; NOT, Boolean negation operator, engagement vetoed when a healthy-restricted antigen is present; OR, Boolean disjunction operator, engagement when any single component is recognized. The operators are capitalized because they name Boolean logic operations and not for emphasis.

uPAR meets the criterion of persistent senescence, as it is induced across multiple senescence models, including therapy-induced, oncogene-induced, and culture-induced, in non-proliferative cells that co-express SA-beta-galactosidase, p16, and the SASP factor IL-6. This co-expression has been validated in human liver fibrosis tissue. Moreover, uPAR satisfies immune accessibility requirements as an intact cell-surface antigen detectable on senescent cells. uPAR products meet these criteria. Regarding the engineered cell (CAR) approach, functional recognition is confirmed, as chimeric antigen receptor-mediated killing is receptor-dependent and reduced by antigen-negative controls. Healthy-state exclusion for the CAR approach is considered partial rather than untested because relevant physiological challenge evidence currently exists: Eskiocak et al. subjected mice to a 15 Gy abdominal irradiation and intestinal injury model, reporting that uPAR CAR-T therapy improved epithelial regeneration, barrier integrity, and mucosal immune function without impairing repair. This provides genuine on-target challenge evidence; however, it does not satisfy the prespecified safety margin across the essential, activated, progenitor, reparative, and beneficially senescent compartments required for a met grade. Additionally, uPAR remains expressed on injury-responsive myeloid and stromal cells, so it only partially meets the criterion. Consequently, the CAR assessment does not meet the standard; the only unmet criterion is healthy-state exclusion. Regarding a prospective vaccine product, functional recognition has not been tested, as no vaccine trials targeting uPAR have been conducted. A positive CAR-T outcome indicates the target but does not validate the vaccine. Similarly, healthy-state exclusion has not been tested for the vaccine route, as this pathway offers no safety margin. Therefore, the vaccine criterion is unmet, with two of the four mandatory criteria remaining untested. The optional telomere axis was not tested for either product. The subsequent evidence differs based on the approach: for the CAR route, a predefined multi-compartment safety margin extending beyond the irradiation model to include infection, surgery, and other repair contexts; for the vaccine route, assessments of human immunogenicity, antigen-dependent clearance, and a route-specific safety margin (Amor et al., 2020; Amor et al., 2024; Eskiocak et al., 2026; Goyer et al., 2026). Applying the quantitative specification in Section 6.2.2, this evidence constitutes a margin demonstrated in one of the seven required compartments, with antigen density unmeasured in all seven, and is now recorded as the grade rather than the undifferentiated description of partial. The required measurement is antigen density on intact cells in calibrated antibody binding capacity units at baseline, 24 h, 72 h, and 7 days after injury in each compartment, reported as the margin computed from the maximum rather than the mean across donors, time points, and activation states and evaluated at the conservative bound of its confidence interval, with functional challenge in at least the inflammation, infection, and progenitor compartments. None of the four quantitative determinants of recognition set out in Section 6.3.2, namely, target density, healthy-compartment density, the effector threshold for this receptor, and receptor affinity, has been reported for uPAR in either route.

GPNMB exhibits a distinct pattern, with its intended human product being a native-surface-antigen vaccine designed to elicit antibodies against the extracellular domain. Consequently, it should be evaluated in terms of the antibody-mediated pathway rather than the HLA-peptide requirements. Its association with persistent senescence is partial: GPNMB is enriched in cells identified as senescent through senescent-cell transcriptomics and shows increased expression in tissue from atherosclerotic patients. Additionally, genetic ablation of GPNMB-positive cells reduces adipose tissue senescence; however, this evidence is limited to population and tissue levels. The co-localization of GPNMB with two or more orthogonal senescence markers within the same cells *in vivo*, an essential criterion for a met grade, is not documented. Furthermore, genetic ablation indicates pathogenicity rather than directly confirming the senescent phenotype of targeted cells. GPNMB’s immune accessibility is supported because it is a transmembrane protein expressed on intact senescent cells, where it was directly detected and used as the basis for antibody targeting rather than inferred from transcript levels. Under the framework’s preclinical grading protocol, functional recognition is satisfied: in murine models, vaccination targeting the GPNMB extracellular domain elicited antibodies and reduced GPNMB-positive cells relative to controls via an antibody-dependent, natural killer-mediated mechanism, exemplifying controlled antigen-dependent clearance through the native-surface pathway. This grading is based on the same preclinical criteria employed for the uPAR CAR assessment. The separate human translation section notes the absence of evidence in humans as unestablished, rather than downgrading the preclinical grade, because human evidence should not be mandatory solely for the vaccine route while preclinical evidence is accepted for the engineered-cell route. The assessment of healthy-state exclusion remains untested, given that no predefined safety margin was demonstrated within essential healthy compartments, and GPNMB is expressed in macrophage lineages, bone-associated cells, and neural cells. The telomere axis remains untested for identical reasons. Accordingly, GPNMB does not meet the framework requirements based on current evidence, because persistent senescence identification is partial and healthy-state exclusion remains untested. The same preclinical grading criteria apply to GPNMB variants extending to cellular routes, including dendritic-cell vaccines and nanovaccines. However, their functional recognition is deemed partial rather than met; they indicate CD8 activation, cytotoxicity, and reduced target cells, but lack evidence of blocking, knockout, carrier-only, or antigen-negative targets that show loss of senolytic clearance when GPNMB recognition is absent, an essential requirement for a met grade. Human translation evidence for these variants is likewise not established. Additional mechanistic research focusing on GPNMB’s biological functions, such as cellular depletion, overexpression, and lysosomal integrity, rather than vaccination strategies, provides context-dependent insights into potential protective and safety considerations, as documented in studies by Suda et al. (2021), Suda et al. (2022), Cao et al. (2024), Zhang et al. (2025), and Sanborn et al. (2025). The co-localization requirement can now be stated numerically rather than descriptively. A met grade on criterion one requires that at least seventy percent of antigen-positive cells within the target lesion co-express at least two orthogonal senescence features, with the lower bound of the exact binomial ninety-five percent confidence interval above fifty percent (Clopper and Pearson, 1934), measured in at least three independent donors with at least two hundred antigen-positive cells scored per donor, with the donor rather than the cell as the unit of analysis and with age-matched non-diseased tissue from the same organ as the comparator. A coverage statement is required alongside specificity: the fraction of the multi-marker senescent compartment that is antigen-positive, with a proposed minimum of thirty percent, because an antigen that is perfectly specific but present in only a small minority of the pathological compartment cannot support clearance. These thresholds are prespecified conventions rather than empirically calibrated values, since no dataset yet links antigen-positive cell burden to therapeutic benefit, and they are declared in advance precisely so that they cannot be chosen after the data are seen. The seventy percent figure is the region in which most engaged cells are in the intended state, even after allowing for the segmentation error and antibody cross-reactivity typical of multiplexed tissue imaging, and the fifty percent lower bound prevents a small or unusually favorable sample from carrying the grade. No published study reports this measurement for GPNMB at single-cell resolution in human tissue.

It is imperative to clarify the limitations of these two cards. Because each candidate omits evaluation of certain criteria, an initial, inaccurate interpretation might be that their combination provides complementary coverage, thereby supporting the rationale for a multivalent vaccine. This inference is incorrect. A conventional multivalent vaccine employs OR logic, inducing immunity against uPAR and GPNMB independently; immunity against uPAR will target uPAR-positive healthy or injury-responsive cells regardless of GPNMB presence. Thus, combining two antigens with unresolved safety profiles in healthy tissues effectively accumulates their respective liabilities. Consequently, the uncharacterized safety of GPNMB in normal tissues and the unresolved injury-cell expression of uPAR are both carried in the combined formulation, potentially increasing the risk to healthy tissues rather than diminishing it. Achieving intersectional specificity, where cytotoxicity occurs solely when both antigens are co-expressed on the same cell, is a feature of engineered AND-gated recognition systems, such as logic-gated chimeric antigen receptors or synthetic-Notch circuits, and cannot be attained through standard vaccination against the same two antigens (Figure 7). While multivalency remains valuable, it is applicable only among individually qualified antigens, specifically to enhance coverage across complementary senotypes or HLA haplotypes and to prevent antigen-loss escape, rather than substituting for the inherent selectivity each component must possess (Amor et al., 2020; Yang et al., 2023; Dai and Gifford, 2023).

Generalizing the exercise to the broader candidate set aligns with this conclusion, provided that the generalization is no stronger than the evidence justifies. NKG2D ligands meet immune accessibility criteria and, in the context of the engineered-cell approach, show functional recognition, as supported by the most advanced cross-species data available. However, their exclusion during healthy states remains unresolved rather than definitively refuted, since these ligands are general stress signals induced on many activated healthy cells; nonetheless, no study has demonstrated antigen-dependent injury or expression above the effector-activation threshold on an essential cell population. Their telomere axis remains unexamined. CD38 meets accessibility criteria but broadly marks functional immunometabolic cells; thus, its exclusion in healthy states remains unresolved. CD153 defines a specific adipose tissue T-cell state that is more selective within that niche but provides limited coverage of senotypes. GPNMB also features cellular-route variants distinct from the antibody-route Suda vaccine, namely, a dendritic-cell vaccine and a self-assembling nanovaccine that engage CD8 responses; these would necessitate their own route-specific protocols. Only uPAR and GPNMB have comprehensive criterion-by-criterion documentation in this context; therefore, it is not asserted that all reviewed candidates have been conclusively shown to fail. A defensible statement is that neither exemplified product meets all four criteria, and no other reviewed candidate has demonstrated compliance with all four criteria within its intended modality. According to the OR-logic argument previously presented, assembling candidates into a conventional multivalent vaccine would not compensate for the missing evidence. A defensible panel can only be assembled from components that inherently satisfy all four criteria and are then combined to achieve coverage or escape resistance, rather than to offset each other’s liabilities (Yang et al., 2023; Deng et al., 2024; Yu et al., 2025; Yoshida et al., 2020; Cao et al., 2024; Zhang et al., 2025; Dai and Gifford, 2023).

## 8 Designing multivalent senolytic vaccines

Multivalency addresses the observed heterogeneity among components that have already met established qualification criteria individually. It does not, however, imply permission to amalgamate self-antigens that individually fail one or more criteria. The fundamental prerequisite for assembling a panel is that each intended component satisfies the four criteria within the selected modality; only then does combination become appropriate. Such a combination aims to target complementary cell lineages, HLA genotypes, or to mitigate antigen-loss escape. As conventional vaccination employs logical OR operations, a panel inherently inherits the off-target liabilities of all its components. Consequently, introducing a component expressed on healthy populations integrates that toxicity into the overall product, irrespective of the other components. The proper comparator for a multivalent panel is therefore the most thoroughly validated single antigen, as well as the smallest and least complex panel. The primary design objective should be to achieve comprehensive coverage and resistance to escape, rather than the intersectional selectivity attainable solely through an engineered AND-gated system (Toussaint et al., 2008; Liu et al., 2020; Dai and Gifford, 2023).

Because recognition mechanisms vary by route, a product must adhere to a specified format rather than assuming that a surface antigen module, multiple HLA-I peptides, and an HLA-II helper module can be combined interchangeably without regard for their individual recognition pathways. A native-surface-antigen vaccine and an HLA-restricted peptide vaccine activate different effector mechanisms, impose distinct tolerance constraints, and follow separate manufacturing processes. The decision to combine such components must be carefully justified and thoroughly characterized, rather than adopted by default. Regardless of the chosen format, each component must independently satisfy the criteria for a healthy state and beneficial senescence in its respective route prior to combination. Platform selection involves balancing definitional precision against durability and delivery considerations. Table 11 provides a comparative overview of various platforms, including defined peptide and protein platforms, mRNA and DNA platforms, viral vectors, heterologous prime-boost strategies, nanoparticle and *in vivo* antigen-presenting cell targeting, and *ex vivo* dendritic cell platforms. Using a defined peptide, protein, or mRNA product is preferable to an undefined lysate as a final translational product, as it permits detailed characterization of identity, dose, mechanism, potency, and cross-reactivity on a component-by-component basis. Conversely, lysate approaches remain valuable during the discovery phase.

**TABLE 11:** Platform options for a multivalent senolytic vaccine, comparing compositional definition, durability, and delivery.

Platform	Strengths	Limitations and best use
Defined peptide/protein	Compositional definition; component-level potency and safety	Weaker immunogenicity in aged recipients; needs adjuvant; best for final defined products (Suda et al., 2021)
mRNA/DNA	Rapid, multivalent, encodes full antigens and helper epitopes	Requires delivery optimization; allows controllable dosing and transient antigen expression, but the induced adaptive immunity is not thereby reversible (Liu et al., 2024)
Viral vector/prime-boost	Strong cellular immunity; broad coverage	Vector immunity; harder to withdraw; discovery and boosting
Nanoparticle/*in vivo* APC targeting	Controls size, density, depot, co-adjuvant; multivalent	Formulation complexity; best for panels in aged recipients (Zhang et al., 2025)
*Ex-vivo* dendritic cell	Bypasses aged-APC defects; potent priming	Individualized manufacturing; complex QC (Cao et al., 2024)
Whole-cell/lysate	Broad polyvalent repertoire; exposes modified epitopes	Undefined composition; batch variability; discovery only (Ichim et al., 2025)

Abbreviations: APC, antigen-presenting cell; DC, dendritic cell; DNA, deoxyribonucleic acid; mRNA, messenger ribonucleic acid; QC, quality control.

Panel assembly must anticipate immunological interactions among components rather than treating them as independent. Immunodominance can cause a dominant epitope to suppress responses to other components, contributing to selectivity or lineage breadth; therefore, component ratios, linker and scaffold design, and delivery methods must be meticulously calibrated to maintain subdominant yet safety-critical responses. Examine sequence similarity among components, as well as between a component and healthy-cell proteins, to minimize unintended cross-reactivity. Furthermore, the panel should encompass mechanistic diversity, for example, by combining a surface-antigen module recognized independently of HLA with HLA-presented peptides, thereby ensuring that escape through antigen loss or presentation defects does not incapacitate the entire immune response. These complex interactions make empirical panel testing essential, because the safety and coverage of a combination cannot be reliably inferred solely from individual components (Toussaint et al., 2008; Liu et al., 2024; Dai and Gifford, 2023; Fritsche et al., 2021).

The primary engineering objective is not to maximize antibody titer, T-cell frequency, peptide count, or predicted coverage in isolation, but to develop a controllable immune response that recognizes multiple independently validated pathological features while remaining below the injury threshold in all essential healthy states. Coordinating antibody, CD8, and CD4 responses requires careful consideration of immunodominance and antigen competition, since a single dominant epitope can inhibit responses to selectivity-conferring components. Furthermore, delivery strategies must account for immunosenescence, which adversely affects dendritic cell phagocytosis, antigen processing, migration, and naive T cell priming in older recipients, the intended population. Multivalent senolytic vaccination becomes credible only when parameters such as breadth, selectivity, mechanism, population equity, and temporal control are collectively optimized and then validated through prospective antigen-dependent cytotoxicity (Cao et al., 2024; Liu et al., 2020; Dai and Gifford, 2023).

Delivery and adjuvant selection are not ancillary formulation details but are pivotal in determining whether a specified panel yields the desired response profile. Lipoplex and lipid-nanoparticle mRNA delivery systems can be adjusted to enhance uptake by antigen-presenting cells and to co-deliver multiple encoded antigens alongside helper epitopes. Furthermore, adjuvant and particle design influence the balance between antibody-mediated and cytotoxic responses. For a senolytic panel intended for elder recipients, delivery must account for age-associated reductions in antigen uptake, processing, and priming, while also avoiding an inflammatory milieu that could itself provoke or exacerbate bystander senescence. These delivery parameters also affect response durability; therefore, a platform that supports dose-controlled, finite-duration responses is preferable in initial human trials. Conversely, reserve durable formats for panels that have demonstrated safety under physiological challenge (Kranz et al., 2016; Kreiter et al., 2015; Cao et al., 2024; Zhang et al., 2025).

### 8.1 A formal comparison of recognition architectures

The claim that conventional multivalency operates on OR logic while intersectional specificity requires AND-gated recognition can be derived rather than asserted. Let a pathological cell carry per-cell engagement probabilities p1 through pn for the panel components, and let a cell of a healthy compartment h carry r1 through rn. Under OR logic, which describes conventional multivalent vaccination and any pool of independently acting effectors, the probability of engagement is one minus the product of the complements of the p terms on the target and one minus the product of the complements of the r terms on healthy cells, and both quantities increase monotonically with panel size. Panel selectivity, defined as the ratio of these two probabilities, is bounded above by the largest individual ratio of p to r in the panel and decreases strictly whenever a component with lower individual selectivity is added. No combination of antigens under OR logic can therefore exceed the selectivity of its best single component, and adding a component with poor healthy-state exclusion necessarily degrades the panel. This inequality, illustrated in Figure 2, is the formal content of the argument that combination cannot rescue candidates that fail individually.

Under AND logic, engagement requires co-expression, so the relevant quantities are joint rather than marginal probabilities. If antigen expression is conditionally independent within the healthy compartment, selectivity approximates the product of the individual selectivities, so two antigens each carrying a tenfold margin yield approximately a hundredfold margin. The qualifier is essential. The multiplicative gain is realized only to the extent that the two antigens are not co-expressed in healthy tissue, and where healthy co-expression is correlated, the gain collapses toward the better single antigen, vanishing entirely in the limit of perfect correlation. The design question for an AND gate is therefore not whether each antigen is individually selective but whether their joint frequency in the healthy compartment is low, and the framework requires that joint healthy-compartment frequency be reported as a measured quantity for any proposed combination.

The conditions of applicability follow from the algebra, are summarized in Table 12, and are illustrated in Figure 2. OR combination is appropriate when every component independently satisfies the four criteria and the limiting problem is coverage of heterogeneous senotypes or resistance to antigen-loss escape; its purpose is breadth and never selectivity. AND gating is appropriate when no single antigen achieves the required healthy-state margin, but a co-expression pattern is rare in healthy tissue, and only where the modality permits engineered recognition, which excludes conventional vaccination and any polyclonal host antibody response. The costs of AND gating are concrete rather than theoretical: induced-expression circuits require priming time and spatial separation between target and healthy cells, and they fail when the two are intermingled, as was shown directly for logic-gated ROR1-directed cells, which produced efficacy without toxicity when normal antigen-positive cells were anatomically segregated from tumor but not when they were co-localized (Srivastava et al., 2019). Both inputs also have their own density thresholds, and manufacturing complexity increases.

**TABLE 12:** Formal comparison of recognition architectures. Selectivity is the ratio of engagement probability on the pathological cell to engagement probability in the healthy compartment. The correct comparator for any multivalent panel is the best qualified single antigen, not the untreated condition.

Architecture	Recognition rule	Target engagement	Healthy-compartment risk	Selectivity scaling with panel size	Modality compatibility	Principal failure mode
OR: conventional multivalent vaccination or pooled effectors	Engage if any component is recognized	One minus the product of the complements of the component probabilities; rises with panel size	The union of all component risks; rises with panel size	Bounded above by the best single component and strictly degraded by any weaker component	Vaccines, polyclonal antibody responses, pooled monospecific products	Accumulation of component liabilities presented as breadth
AND: logic-gated engineered receptors	Engage only if both inputs are present on the same cell	The joint probability; falls if the two antigens are not reliably co-expressed on target cells	The joint probability in the healthy compartment	Approximately multiplicative when healthy co-expression is independent; collapses toward the better component when it is correlated	Engineered cells only; not achievable by vaccination	Priming delay, and failure where target and healthy cells are intermingled (Srivastava et al., 2019)
NOT: inhibitory receptor veto	Engage unless a healthy-restricted antigen is present	Unchanged from the single activating antigen	Reduced in proportion to the coverage and reliability of the veto antigen	Does not scale with panel size; scales with the completeness of the healthy-restricted marker	Engineered cells only	Incomplete veto coverage leaves unprotected healthy populations (Fedorov et al., 2013)
Single qualified antigen	Engage if the antigen is recognized	The single component probability	The single component risk	Not applicable; this is the comparator against which every panel must be judged	All routes	Antigen-loss escape and incomplete senotype coverage

Abbreviations: AND, boolean conjunction operator, engagement only when both inputs are present on the same cell; CAR, chimeric antigen receptor; NOT, boolean negation operator, engagement vetoed when a healthy-restricted antigen is present; OR, boolean disjunction operator, engagement when any single component is recognized. The operators are capitalized because they name Boolean logic operations and not for emphasis.

Two further architectures belong in the comparison. NOT gating with inhibitory receptors addresses healthy-cell sparing directly by using a healthy-restricted antigen as a veto (Fedorov et al., 2013), an approach with particular appeal in senescence because healthy-restricted markers may prove easier to define than senescence-restricted ones, and its protection is limited by the completeness of the veto marker rather than by the quality of the activating antigen. Proximal-signaling receptor designs have improved the fidelity of Boolean gating relative to earlier synthetic Notch implementations (Roybal et al., 2016; Tousley et al., 2023). Across all four architectures, the correct comparator for a panel is the best-qualified single antigen rather than the untreated condition, which is the comparison the field has generally failed to make.

## 9 Validation, translation, safety, and equity

Computational prioritization narrows the molecular search space but generates hypotheses rather than validated targets. Prospective validation should proceed as a sequence of independent evidence gates: orthogonal confirmation of the persistent pathological senescent state and, where the target product profile has selected a mechanistic stratum, confirmation of that specific mechanism, which is direct telomere dysfunction solely for a telomere-stratified product and the corresponding oncogenic, mitochondrial, inflammatory, or therapy-induced signature otherwise; molecular verification of surface antigens via intact-cell detection and of HLA peptides through targeted immunopeptidomics; functional immune-recognition assays demonstrating antigen-dependent cytotoxicity with blocking, knockout, and antigen-negative controls; healthy-state and off-target counter-screening in primary human cells, organoids, and tissues; *in vivo* validation with consideration of species-specific differences; and efficacy endpoints where the therapeutic benefit is lost upon depletion of the responsible effector or deletion of the target. Each stage should include explicit go/no-go criteria, and results should be documented under version control with standardized identifiers to enable future benchmarking of the framework against experimental outcomes (Purcell et al., 2019; Suryadevara et al., 2024; Wilkinson et al., 2016; Dekkers et al., 2023).

Translation initiation should target a serious disease with a rich biomarker profile, a substantial burden of pathological senescent cells, direct tissue access, measurable pharmacodynamic markers, and a short-term, clinically meaningful endpoint. This approach supports a more justifiable benefit-risk assessment than vaccinating otherwise healthy adults against a broadly defined aging phenotype. The design of first-in-human studies should prioritize the most controllable modality, defined operationally: reversible indicates that the administered agent functions solely within a specified interval and can be halted or dose-adjusted; controllable signifies that its activity can be modulated or deactivated; and durable refers to self-amplifying or long-lived activity that cannot be readily terminated. These properties are modality-specific rather than absolute, acknowledging that even a finite-duration formulation may induce persistent immune priming; thus, a reversible platform mitigates but does not eliminate durable risks. The assertion that a finite-duration antibody trial could support a subsequent vaccine must therefore be qualified. Because an antibody and a vaccine differ in effector mechanisms, tissue penetration, tolerance engagement, pharmacodynamics, and reversibility, a favorable antibody outcome supports the biological hypothesis that removing an antigen-positive population is advantageous. However, it does not validate a vaccine targeting the same antigen, which must separately demonstrate tolerance, immunogenicity, and durability. Early definition of manufacturing protocols, potency assurance measures, and product comparability is essential, especially for cellular and multivalent products, where potency depends on the balance of components (Cummings and Kritchevsky, 2022; Amor et al., 2024a; Zhao et al., 2024; Justice et al., 2018).

Safety for long-lived, self-directed immunity requires an exceptionally high standard. Short-term assessments such as histology, serum chemistry, body weight, and autoantibody measurements are necessary but not sufficient. Relevant models should encompass infection challenge, vaccination response, wound healing, surgical recovery, regeneration, latent-virus control, autoimmunity-prone backgrounds, and long-term tumor surveillance, conducted in aged animals whose target burden and compensatory capacity differ from those of young, healthy animals. Ethical considerations stem from the intervention’s enduring nature and the fact that some recipients might later require suppression of the beneficial aspects of senescence. Therefore, informed consent must address issues of irreversibility and delayed risks, in accordance with contemporary research ethics guidelines (Grosse et al., 2020; Zhao et al., 2024; World Medical Association, 2025; Percie du Sert et al., 2020).

Regulatory translation requires establishing endpoints and product-characterization standards before initial human testing, rather than retroactively incorporating them. Geroscience-oriented interventions currently lack a universally accepted clinical endpoint; therefore, a senolytic vaccine initiative should incorporate disease-specific functional and biomarker endpoints aligned with the selected indication. It should also prespecify pharmacodynamic evidence of senescent-cell reduction, consistent with emerging guidelines on trial design for aging-related interventions. Cellular and gene-based senolytic products also require clearly defined potency assurance measures, long-term follow-up to assess durability or integration, and comparability strategies that account for a multivalent product’s potency dependence on component balance. To align the framework’s evidence gates with regulatory expectations, it is crucial to distinguish final guidance from draft recommendations issued for comment and marked as not for implementation, which reflect the regulatory agency’s evolving perspective but do not yet constitute definitive positions. The potency-assurance recommendations referenced herein are of the latter, draft category. Generally, guidance documents, regardless of their status, describe current regulatory thinking rather than impose legally binding obligations, unless they explicitly restate statutory or regulatory requirements. Recognizing this distinction, aligning the framework with expectations pertaining to potency, safety follow-up, and inclusive enrollment enhances both scientific integrity and the pathway toward approval (Cummings and Kritchevsky, 2022; Rolland et al., 2023; Food and Drug Administration, 2020; 2023; European Medicines Agency, 2025; National Institutes of Health, 2019).

Population equity is a scientific imperative as much as an ethical obligation, because HLA-restricted components inherit the allele bias inherent in immunopeptidome data and the training datasets used for prediction models. Alleles prevalent in underrepresented populations tend to have less extensive eluted-ligand evidence and exhibit lower predictive accuracy. Consequently, a panel calibrated on well-characterized alleles may systematically underperform when applied to other populations. Therefore, achieving equity requires designing panels that account for allele frequencies, reporting predicted coverage across diverse populations, and broadening immunopeptidomic data to include underrepresented alleles. Additionally, researchers must carefully consider the access and affordability constraints that currently impede the use of engineered cell therapies (Sarkizova et al., 2020; Atkins et al., 2024; Toussaint et al., 2008; Ahmed et al., 2022). The evidence-based sequence in Figure 8 outlines these requirements hierarchically, from foundational stages to targeted convergence toward disease-specific human testing, with progression governed by explicit entry and exit criteria rather than chronological timelines. Reversible modalities are prioritized before establishing durable, self-directed immunity; no stage signals readiness for human testing until all mandated criteria are satisfactorily met or thoroughly tested. Table 13 documents the controversies whose resolution substantially influences candidate selection, trial design, and interpretative analyses.

**FIGURE 8:**
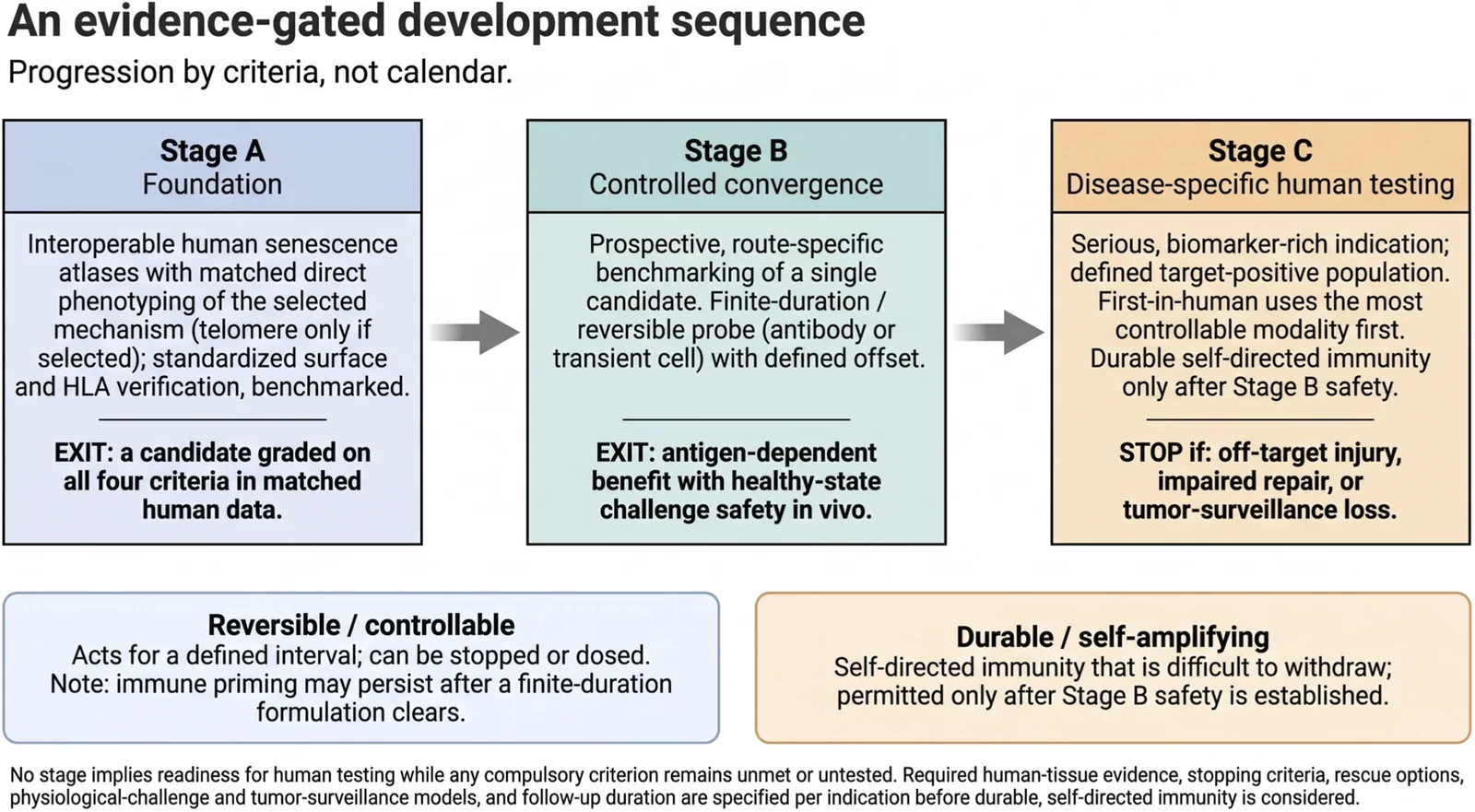
An evidence-gated development sequence. Progression is governed by explicit entry and stopping criteria rather than calendar dates. Reversible and controllable modalities, defined through operational parameters, precede the establishment of durable self-directed immunity. It is worth noting that immune priming may persist even after a formulation of finite duration has been cleared. No stage indicates readiness for human testing until all mandatory criteria are met and thoroughly evaluated; these include required human-tissue evidence, stopping and rescue criteria, physiological-challenge and tumor-surveillance models, and specified follow-up durations for each indication. Abbreviations: HLA, human leukocyte antigen.

**TABLE 13:** Major controversies in bioinformatics-guided seno-antigen and vaccine development, with the decisive experiment and provisional resolution supported by current evidence.

Controversy	Decisive experiment	Provisional resolution
Universal antigen versus intersectional target	Lineage-resolved functional targeting across senotypes and tissues	Specificity arises from healthy-state exclusion by each individual antigen or from an engineered AND-gated recognition system; a conventional combination improves coverage or reduces antigen-loss escape only after every component is independently selective (Sanborn et al., 2025; Zhao et al., 2024)
Telomere length versus dysfunction	Direct damage, shortest-end, and perturbation-rescue assays paired with expression	Antigen discovery needs a state variable near the dysfunction axis, not a bulk mean (Rossiello et al., 2022)
Presentation versus immunogenicity	Functional recognition and cross-reactivity in HLA-diverse primary cells	Presentation and prediction cannot infer function; recognition stays experimental (Purcell et al., 2019; Goyer et al., 2026)
Durable memory versus controllability	Physiological-challenge safety before committing durable immunity	Durability is an advantage only after challenge safety; reversible platforms precede irreversible ones (Amor et al., 2024a; Grosse et al., 2020)
Disease treatment versus preventive vaccination	Disease-specific human benefit and long-term repair competence	Begin in serious, target-rich disease; prevention is conditional on a stronger evidence base (Cummings and Kritchevsky, 2022; World Medical Association, 2025)

Abbreviations: AND, boolean conjunction operator denoting engagement only when both specified inputs are present on the same cell; HLA, human leukocyte antigen.

### 9.1 Population-level determinants of translational potential

Population-level factors enter the framework as four distinct requirements rather than a single equity consideration, because they fail in different ways and require different measures.

The first is allele coverage. For any HLA-restricted component, the evidence card reports population coverage computed from allele frequencies in the intended population using curated frequency data, reported per population rather than as a single global figure (Gonzalez-Galarza et al., 2020; Toussaint et al., 2008). A candidate whose coverage exceeds eighty percent in one population and falls below forty percent in another is not one product with a caveat but a different product in each setting, and must be described that way. The second is the reliability of the coverage estimate itself. Predictor accuracy varies across alleles, and alleles with the thinnest eluted-ligand support are disproportionately common in populations underrepresented in immunopeptidomic datasets (Sarkizova et al., 2020; Atkins et al., 2024), so a coverage figure computed from predictions alone is a hypothesis rather than a measurement. Allele-level confidence is therefore reported alongside coverage, and the estimate is downgraded explicitly where eluted-ligand evidence is absent for the alleles carrying that coverage.

The third is inter-individual and inter-context variation in the presenting cell, which is distinct from allele frequency and is often neglected. Presentation by senescent cells is not a fixed property: total HLA class I surface level varies with interferon tone, with the inducing stress, with cell type, and with age-associated changes in proteasome composition and antigen-processing machinery, and senescent cells upregulate inhibitory ligands, including HLA-E that alter natural killer recognition independently of the peptide repertoire (Pereira et al., 2019; Goyer et al., 2026). The framework therefore requires that immunopeptidomic and functional evidence derive from more than one donor and more than one inducer before criterion two or criterion three is graded as met, and that total class I surface level be quantified alongside the peptide, because a given copy number on a class I low cell and on a class I high cell are not the same antigen from the effector’s point of view.

The fourth is that surface-antigen routes are not exempt from population variation merely because they are HLA-independent. Expression levels vary between individuals, and Fc receptor polymorphism substantially alters the potency of antibody-dependent cellular cytotoxicity (Tang et al., 2007), so a native-surface-antigen vaccine whose mechanism is antibody-dependent clearance inherits a population-dependent effect size even without allele restriction. Translational potential is therefore reported as a coverage-adjusted statement naming the population, the alleles or genotypes on which the estimate rests, and the confidence attached to each, rather than as a single global claim. For uPAR and GPNMB, both surface-antigen-route candidates, allele considerations do not apply while expression-variation and Fc polymorphism considerations do, and neither has been characterized across populations.

### 9.2 A prospective test of the framework itself

A decision framework that cannot be falsified is not a scientific contribution, so this study specifies how to test it rather than leaving it as a general call for validation. The design is a prospective, pre-registered, blinded benchmarking study in three steps.

Step one assembles a candidate set of twenty to thirty senescence-associated antigens spanning the full grade range, drawn from published discovery datasets, and grades each against the rubric using only evidence available at a fixed cutoff date, with grading performed independently by two investigators blinded to downstream outcomes and agreement reported as a chance-corrected coefficient (Landis and Koch, 1977). Step two pre-registers the framework’s predictions as an ordered list with explicit predicted outcomes for each candidate: candidates graded met on all four criteria are predicted to show antigen-dependent clearance of the target population with sparing of the healthy panel; candidates carrying a hard stop on healthy-state exclusion are predicted to show antigen-dependent injury in the named compartment; and candidates graded untested on accessibility are predicted to fail intact-cell verification at a rate substantially exceeding that of graded-met candidates. Step three runs a uniform experimental panel on every candidate, regardless of grade, which is the methodological requirement that makes the study informative, since testing only promising candidates would estimate sensitivity while leaving specificity unknown. The uniform panel comprises intact-cell surface quantification or targeted immunopeptidomics, route-appropriate functional recognition with antigen-dependence controls, and Table 7 healthy-compartment panel with at least one *in vivo* physiological challenge.

Table 14 states the success and falsification criteria in advance. The framework is supported if the positive predictive value of a met-on-all-four grade for antigen-dependent clearance with healthy sparing exceeds 0.7, if the negative predictive value of a hard stop exceeds 0.9, and if the grade ordering predicts experimental outcome better than a differential expression ranking of the same candidates by a prespecified margin in the area under the receiver operating characteristic curve. The framework is falsified in its current form if graded-met and graded-untested candidates show indistinguishable outcome rates, or if fold-change ranking performs as well as the grades, since either result would demonstrate that the criteria add no information beyond differential abundance, which is precisely the claim made on their behalf. Every assay in the uniform panel is in routine use, so the limiting resources are matched human tissue and immunopeptidomic capacity rather than methodological novelty. A study of this scale exceeds a single laboratory and is proposed as a community benchmark with deposited data and a public candidate registry, structured as epitope prediction benchmarks are structured, so that the framework can be assessed by parties who did not construct it (Wilkinson et al., 2016; Perez-Riverol et al., 2025).

**TABLE 14:** Predetermined success and falsification criteria for the prospective test of the framework described in Section 9.2. Predictions are registered before the uniform experimental panel is executed, and the panel is run on every candidate regardless of grade so that specificity as well as sensitivity can be estimated.

Prediction under test	Measurement	Supports the framework	Falsifies the framework
A met grade on all four criteria predicts antigen-dependent clearance with healthy sparing	Positive predictive value across the uniform experimental panel	Positive predictive value above 0.7	Positive predictive value at or near chance given the base rate
A hard stop predicts antigen-dependent injury in the named compartment	Negative predictive value of the hard stop	Negative predictive value above 0.9	Hard-stop candidates show no excess injury relative to others
Grade ordering outperforms differential expression ranking	Difference in area under the receiver operating characteristic curve, prespecified margin of 0.1	Grade ordering superior by at least the prespecified margin	Fold-change ranking equals or exceeds grade ordering
An untested grade on accessibility predicts failure at intact-cell verification	Failure rate at intact-cell verification, untested versus met candidates	Substantially higher failure rate among untested candidates	Failure rates indistinguishable between grades
Grading is reproducible between investigators	Chance-corrected agreement between two blinded graders	Agreement in the substantial range or above	Agreement below the moderate range, indicating that the rubric is not operational

Abbreviations: AUC, area under the curve; NPV, negative predictive value; PPV, positive predictive value; ROC, receiver operating characteristic.

## 10 Discussion and conclusion

The evidence synthesized here supports modifying the central research inquiry. The field should not ask whether a universal marker can identify all senescent cells and be adapted into a therapeutic antigen; rather, it should investigate which combinations of cellular state, initiating mechanism, tissue context, immune accessibility, and healthy-state exclusion delineate a clinically targetable population. Telomere dysfunction remains a significant mechanistic axis, as critically short, uncapped, oxidatively damaged, or transcriptionally perturbed telomeres evoke persistent DNA damage signaling and senescence. However, it is neither equivalent to average telomere shortening nor solely responsible for every pathological senescent population. Its primary value in antigen discovery lies in defining a biologically coherent subclass that can be contrasted with other mechanisms (Ogrodnik et al., 2024; Rossiello et al., 2022; Sanborn et al., 2025).

Immune-mediated senolysis is achievable; however, its implementation depends on antigen specificity, contextual factors, administration routes, and technological platforms. Engineered cells, vaccines, and antibodies can eliminate specific senescence-associated cell populations and enhance preclinical outcomes. Nonetheless, human data indicate that immunogenicity varies by cell type and inducer, and evidence seldom transfers seamlessly across therapeutic routes. Currently, no intervention targets an antigen validated against all four criteria within the intended modality. This contribution is not a scoring system or an operational pipeline but a conceptual decision architecture. This architecture distinguishes target-state evidence from factors such as accessibility, functional recognition, safety in healthy tissues, and population coverage. It separates target nomination from product validation and grades each criterion, including an explicit category for untested aspects. When applied to uPAR and GPNMB as illustrative examples of distinct routes, the framework yields a transparent, intentionally critical outcome: no product candidate passes the assessment; the telomere axis remains untested for each; and no other reviewed candidate has demonstrated the capacity to meet all four criteria within its respective modality. The example identifies the necessary experiments for each candidate, without endorsing any for development (Amor et al., 2020; Amor et al., 2024; Yang et al., 2023; Goyer et al., 2026; Dai and Gifford, 2023).

Several controversies remain genuinely open, including whether a broadly recurrent senescence core exists, how directly telomere dysfunction should be measured, the limits of predictive presentation, the balance between durable memory and therapeutic controllability, and whether translation should begin with disease treatment or preventive vaccination. Each changes candidate selection and trial design, and each is resolvable by a defined experiment rather than by argument. The most credible path is a staged program that moves from standardized human target definition to prospective candidate benchmarking, validated disease-specific panels, reversible human target testing, and, only then, durable multivalent vaccination (Zhao et al., 2024; Goyer et al., 2026; Cummings and Kritchevsky, 2022).

This review possesses limitations that constrain its conclusions. As a structured narrative synthesis rather than a systematic review involving quantitative pooling, it remains susceptible to selection and interpretation biases that, although mitigated by documentation and quality self-audit procedures, cannot be entirely eradicated. Furthermore, the heterogeneity of the underlying endpoints renders formal meta-analysis unsuitable. The described framework is a decision architecture rather than a validated algorithm; its core components, matched-sample reference sets, extensive direct telomere phenotyping, and standardized functional immunogenicity assays, are emerging capabilities, so the illustrative example is provisional. Its gate assignments are expected to evolve as additional matched data become available. The proposed pipeline has yet to undergo prospective benchmarking against experimental outcomes, a pertinent next step. Lastly, a significant portion of primary evidence derives from murine models, and due to species-specific differences in markers of senescence, HLA biology, immunosenescence, and repair mechanisms, human validation cannot be presumed based on preclinical efficacy (Snyder, 2019; Baethge et al., 2019; Goyer et al., 2026; Suryadevara et al., 2024).

In conclusion, pathological senescence constitutes a heterogeneous target class, and a clinically useful senescence-antigen must satisfy, within the same cells and in the intended modality, four essential criteria: persistent pathological senescence, verified surface or HLA accessibility, functional immune recognition, and strict exclusion from critical healthy and reparative states. Mechanistic origin, including telomere dysfunction, offers a valuable optional stratification axis for defining coherent subsets; however, it is not obligatory and should be documented as untested when matched data are unavailable. The framework presented herein is a proposed, unvalidated conceptual decision architecture, not an implementation or optimization pipeline. It has not been operationalized or benchmarked, and such efforts are identified as necessary future work. Based on current evidence, no developed example product meets all four criteria, and no other reviewed candidate has yet demonstrated the capacity to satisfy these criteria within its intended modality. Furthermore, because typical multivalent vaccination combinations combine components rather than intersecting them, combination strategies cannot replace the selectivity each component inherently possesses. This framework enhances the selectivity and transparency of development processes and directs experiments toward the most influential evidence gaps to inform progression decisions. Nevertheless, it cannot replace direct antigen presentation, functional immune recognition, selective cytotoxicity, safety under physiological challenge, or clinical efficacy. Accordingly, the worked example outlines the experiments needed before progression, rather than endorsing the development of multivalent senolytic vaccines (Purcell et al., 2019; Rossiello et al., 2022; Ogrodnik et al., 2024; Wilkinson et al., 2016).

## Glossary

8-oxoG: 8-oxoguanine
ADC: antibody-drug conjugate
ADCC: antibody-dependent cellular cytotoxicity
AND: Boolean conjunction operator
APC: antigen-presenting cell
ApoE: apolipoprotein E
ATM: ataxia-telangiectasia mutated
ATR: ATM- and Rad3-related
AUC: area under the curve
CAR: chimeric antigen receptor
CCl4: carbon tetrachloride
CD: cluster of differentiation
CDKN1A: cyclin-dependent kinase inhibitor 1A
CDKN2A: cyclin-dependent kinase inhibitor 2A
cGAS: cyclic GMP-AMP synthase
c-ABL: cellular Abelson tyrosine-protein kinase
DC: dendritic cell
DDR: DNA-damage response
DNA: deoxyribonucleic acid
EC50: half-maximal effective concentration
EV: extracellular vesicle
Fc: fragment crystallizable region of an immunoglobulin
FDR: false discovery rate
FISH: fluorescence *in situ* hybridization
Flow-FISH: flow cytometry combined with fluorescence *in situ* hybridization
GD3: disialoganglioside 3
GMP-AMP: guanosine monophosphate-adenosine monophosphate
GPNMB: glycoprotein nonmetastatic melanoma protein B
Gy: gray
HFD: high-fat diet
HLA: human leukocyte antigen
HLA-E: human leukocyte antigen E
IgM: immunoglobulin M
IL-18: interleukin-18
Ki-67: marker of proliferation Ki-67
LGR5: leucine-rich repeat-containing G-protein-coupled receptor 5;
mRNA: messenger ribonucleic acid
NHP: non-human primate
NK: natural killer
NKG2D: natural killer group 2 member D
NOT: Boolean negation operator
NPV: negative predictive value
OR: Boolean disjunction operator
p16: cyclin-dependent kinase inhibitor 2A protein product
p21: yclin-dependent kinase inhibitor 1A protein product
p53: tumor protein p53
PanIN: pancreatic intraepithelial neoplasia
PCR: polymerase chain reaction
PD-L1: programmed death-ligand 1
PD-L2: programmed death-ligand 2
POT1: protection of telomeres 1
PPV: positive predictive value
PRISMA: Preferred Reporting Items for Systematic Reviews and Meta-Analyses
Q-FISH: quantitative fluorescence *in situ* hybridization
qPCR: quantitative polymerase chain reaction
QC: quality control
RB: retinoblastoma protein
RNA: ribonucleic acid
ROC: receiver operating characteristic
ROS: reactive oxygen species
SA-beta-galactosidase: senescence-associated beta-galactosidase
SANRA: Scale for the Assessment of Narrative Review Articles
SASP: senescence-associated secretory phenotype
STELA: single telomere length analysis
STING: stimulator of interferon genes
TAF: telomere-associated DNA damage focus
TCR: T-cell receptor
TERRA: telomeric repeat-containing RNA
TeSLA: telomere shortest length assay
TFIIS: transcription elongation factor IIS
TIF: telomere dysfunction-induced focus
TIN2: TRF1-interacting nuclear factor 2
TPP1: TIN2-interacting protein 1
TRF: terminal restriction fragment
TRF1: telomeric repeat-binding factor 1
TRF2: telomeric repeat-binding factor 2
uPAR: urokinase-type plasminogen activator receptor
UT: untested
YAP1: Yes-associated protein 1; The Boolean operators AND, OR, and NOT are capitalized throughout because they denote logic operations that define a recognition rule, not emphasis.

## References

[B1] Acosta J. C. Banito A. Wiggins H. Georgilis A. Hay T. Athineos D. (2013). A complex secretory program orchestrated by the inflammasome controls paracrine senescence. Nat. Cell Biol. 15, 978–990. 10.1038/ncb2784 PMC3732483 23770676

[B2] Adams C. Gabriel W. Laukens K. Picciani M. Wilhelm M. Bittremieux W. (2024). Fragment ion intensity prediction improves the identification rate of non-tryptic peptides in timsTOF. Nat. Commun. 15, 3956. 10.1038/s41467-024-48322-0 PMC11087512 38730277

[B3] Ahmed N. Shahzad M. Shippey E. Bansal R. Mushtaq M. U. Mahmoudjafari Z. (2022). Socioeconomic and racial disparity in chimeric antigen receptor T cell therapy access. Transplant. Cell. Ther. 28 (7), 358–364. 10.1016/j.jtct.2022.04.008 35429662

[B4] Alanio C. Lemaitre F. Law H. K. W. Hasan M. Albert M. L. (2010). Enumeration of human antigen-specific naive CD8+ T cells reveals conserved precursor frequencies. Blood 115, 3718–3725. 10.1182/blood-2009-10-251124 20200354

[B5] Amor C. Feucht J. Leibold J. Ho Y. J. Zhu C. Alonso-Curbelo D. (2020). Senolytic CAR T cells reverse senescence-associated pathologies. Nature 583, 127–132. 10.1038/s41586-020-2403-9 32555459 PMC7583560

[B6] Amor C. Fernández-Maestre I. Chowdhury S. Ho Y. J. Nadella S. Graham C. (2024a). Prophylactic and long-lasting efficacy of senolytic CAR T cells against age-related metabolic dysfunction. Nat. Aging 4, 336–349. 10.1038/s43587-023-00560-5 PMC10950785 38267706

[B7] Amor C. Feucht J. Leibold J. Ho Y.-J. Zhu C. Alonso-Curbelo D. (2024b). Author correction: senolytic CAR T cells reverse senescence-associated pathologies. Nature 627, E9. 10.1038/s41586-024-07197-3 38383793

[B8] Anerillas C. Altés G. Grešová K. Tsitsipatis D. Mazan-Mamczarz K. Banerjee R. (2026). SenCat: cataloging human cell senescence through multi-omic profiling of multiple senescent primary cell types. Mol. Cell 86 (13), 2605–2616.e8. 10.1016/j.molcel.2026.05.017 PMC13267859 42276073

[B9] Atkins T. K. Solanki A. Vasmatzis G. Cornette J. Riedel M. (2024). Evaluating NetMHCpan performance on Non-European HLA alleles not present in training data. Front. Immunol. 14, 1288105. 10.3389/fimmu.2023.1288105 PMC10825027 38292493

[B10] Azzalin C. M. Reichenbach P. Khoriauli L. Giulotto E. Lingner J. (2007). Telomeric repeat containing RNA and RNA surveillance factors at mammalian chromosome ends. Science 318, 798–801. 10.1126/science.1147182 17916692

[B11] Baethge C. Goldbeck-Wood S. Mertens S. (2019). SANRA–a scale for the quality assessment of narrative review articles. Res. Integr. Peer Rev. 4, 5. 10.1186/s41073-019-0064-8 30962953 PMC6434870

[B12] Baird D. M. Rowson J. Wynford-Thomas D. Kipling D. (2003). Extensive allelic variation and ultrashort telomeres in senescent human cells. Nat. Genet. 33, 203–207. 10.1038/ng1084 12539050

[B13] Baker D. J. Wijshake T. Tchkonia T. LeBrasseur N. K. Childs B. G. van de Sluis B. (2011). Clearance of p16^Ink4a^-positive senescent cells delays ageing-associated disorders. Nature 479, 232–236. 10.1038/nature10600 22048312 PMC3468323

[B14] Baker D. J. Childs B. G. Durik M. Wijers M. E. Sieben C. J. Zhong J. (2016). Naturally occurring p16^Ink4a^-positive cells shorten healthy lifespan. Nature 530, 184–189. 10.1038/nature16932 26840489 PMC4845101

[B15] Barnes R. P. de Rosa M. Thosar S. A. Detwiler A. C. Roginskaya V. Van Houten B. (2022). Telomeric 8-oxo-guanine drives rapid premature senescence in the absence of telomere shortening. Nat. Struct. and Mol. Biol. 29, 639–652. 10.1038/s41594-022-00790-y PMC9287163 35773409

[B16] Basisty N. Kale A. Jeon O. H. Kuehnemann C. Payne T. Rao C. (2020). A proteomic atlas of senescence-associated secretomes for aging biomarker development. PLoS Biol. 18, e3000599. 10.1371/journal.pbio.3000599 31945054 PMC6964821

[B17] Bausch-Fluck D. Goldmann U. Müller S. van Oostrum M. Müller M. Schubert O. T. (2018). The *in silico* human surfaceome. Proc. Natl. Acad. Sci. U. S. A. 115, E10988–E10997. 10.1073/pnas.1808790115 30373828 PMC6243280

[B18] Bramer W. M. de Jonge G. B. Rethlefsen M. L. Mast F. Kleijnen J. (2018). A systematic approach to searching: an efficient and complete method to develop literature searches. J. Med. Libr. Assoc. 106, 531–541. 10.5195/jmla.2018.283 30271302 PMC6148622

[B19] Bulek A. M. Cole D. K. Skowera A. Dolton G. Gras S. Madura F. (2012). Structural basis for the killing of human beta cells by CD8+ T cells in type 1 diabetes. Nat. Immunol. 13, 283–289. 10.1038/ni.2206 22245737 PMC3378510

[B20] Cao Y. Du X. Yu J. Wang Y. Jin X. Gu B. (2024). Seno-antigen-pulsed dendritic cell vaccine induce anti-aging immunity to improve adipose tissue senescence and metabolic abnormalities. Biomed. and Pharmacother. 179, 117433. 10.1016/j.biopha.2024.117433 39260327

[B21] Carreno B. M. Magrini V. Becker-Hapak M. Kaabinejadian S. Hundal J. Petti A. A. (2015). A dendritic cell vaccine increases the breadth and diversity of melanoma neoantigen-specific T cells. Science 348, 803–808. 10.1126/science.aaa3828 25837513 PMC4549796

[B22] Cawthon R. M. (2002). Telomere measurement by quantitative PCR. Nucleic Acids Res. 30, e47. 10.1093/nar/30.10.e47 12000852 PMC115301

[B23] Chakravarti D. LaBella K. A. DePinho R. A. Rashid A. Li J. Li J. (2020). Telomere dysfunction activates YAP1 to drive tissue inflammation. Nat. Commun. 11, 4766. 10.1038/s41467-020-18420-w PMC7505960 32958778

[B24] Clopper C. J. Pearson E. S. (1934). The use of confidence or fiducial limits illustrated in the case of the binomial. Biometrika 26, 404–413. 10.1093/biomet/26.4.404

[B25] Cummings S. R. Kritchevsky S. B. (2022). Endpoints for geroscience clinical trials: health outcomes, biomarkers, and biologic age. GeroScience 44, 2925–2931. 10.1007/s11357-022-00671-8 PMC9768060 36260264

[B26] Cvjetkovic A. Jang S. C. Konečná B. Höög J. L. Sihlbom C. Lässer C. (2016). Detailed analysis of protein topology of extracellular vesicles–evidence of unconventional membrane protein orientation. Sci. Rep. 6, 36338. 10.1038/srep36338 27821849 PMC5099568

[B27] Dai Z. Gifford D. K. (2023). Constrained submodular optimization for vaccine design. Proc. AAAI Conf. Artif. Intell. 37, 5045–5053. 10.1609/aaai.v37i4.25632

[B28] de Lange T. (2005). Shelterin: the protein complex that shapes and safeguards human telomeres. Genes and Dev. 19, 2100–2110. 10.1101/gad.1346005 16166375

[B29] de Lange T. (2018). Shelterin-mediated telomere protection. Annu. Rev. Genet. 52, 223–247. 10.1146/annurev-genet-032918-021921 30208292

[B30] Dekkers J. F. Alieva M. Wellens L. M. Ariese H. C. R. Jamieson P. R. Vonk A. M. (2023). Uncovering the mode of action of engineered T cells in patient cancer organoids. Nat. Biotechnol. 41, 60–69. 10.1038/s41587-022-01397-w PMC9849137 35879361

[B31] Demaria M. Ohtani N. Youssef S. A. Rodier F. Toussaint W. Mitchell J. R. (2014). An essential role for senescent cells in optimal wound healing through secretion of PDGF-AA. Dev. Cell 31, 722–733. 10.1016/j.devcel.2014.11.012 25499914 PMC4349629

[B32] Deng Y. Kumar A. Xie K. Schaaf K. Scifo E. Morsy S. (2024). Targeting senescent cells with NKG2D-CAR T cells. Cell Death Discov. 10, 217. 10.1038/s41420-024-01976-7 PMC11069534 38704364

[B33] di Fagagna F. A. Reaper P. M. Clay-Farrace L. Fiegler H. Carr P. von Zglinicki T. (2003). A DNA damage checkpoint response in telomere-initiated senescence. Nature 426, 194–198. 10.1038/nature02118 14608368

[B34] Dou Z. Ghosh K. Vizioli M. G. Zhu J. Sen P. Wangensteen K. J. (2017). Cytoplasmic chromatin triggers inflammation in senescence and cancer. Nature 550, 402–406. 10.1038/nature24050 28976970 PMC5850938

[B35] Eskiocak O. Gewolb J. Shah V. Rouse J. A. Chowdhury S. Akyildiz E. O. (2026). Anti-uPAR CAR T cells reverse and prevent aging-associated defects in intestinal regeneration and fitness. Nat. Aging 6, 108–126. 10.1038/s43587-025-01022-w PMC12823409 41291258

[B36] European Medicines Agency (2025). Guideline on Quality, Non-clinical and Clinical Requirements for Investigational Advanced Therapy Medicinal Products in Clinical Trials. EMA/CAT/22473/2025. Available online at: https://www.ema.europa.eu/en/guideline-quality-non-clinical-clinical-requirements-investigational-advanced-therapy-medicinal-products-clinical-trials-scientific-guideline (Accessed July 16, 2026).

[B37] Fedorov V. D. Themeli M. Sadelain M. (2013). PD-1- and CTLA-4-based inhibitory chimeric antigen receptors (iCARs) divert off-target immunotherapy responses. Sci. Transl. Med. 5, 215ra172. 10.1126/scitranslmed.3006597 24337479 PMC4238416

[B38] Ferrari R. (2015). Writing narrative style literature reviews. Med. Writ. 24, 230–235. 10.1179/2047480615z.000000000329

[B39] Food and Drug Administration (2020). Long Term Follow-up After Administration of Human Gene Therapy Products: Guidance for Industry. Available online at: https://www.fda.gov/regulatory-information/search-fda-guidance-documents/long-term-follow-after-administration-human-gene-therapy-products (Accessed July 16, 2026).

[B40] Food and Drug Administration (2023). Potency Assurance for Cellular and Gene Therapy Products: Draft Guidance for Industry. Available online at: https://www.fda.gov/regulatory-information/search-fda-guidance-documents/potency-assurance-cellular-and-gene-therapy-products (Accessed July 16, 2026).

[B41] Frescas D. Roux C. M. Aygun-Sunar S. Gleiberman A. S. Krasnov P. Kurnasov O. V. (2017). Senescent cells expose and secrete an oxidized form of membrane-bound vimentin as revealed by a natural polyreactive antibody. Proc. Natl. Acad. Sci. U. S. A. 114, E1668–E1677. 10.1073/pnas.1614661114 28193858 PMC5338544

[B42] Fritsche J. Kowalewski D. J. Backert L. Gwinner F. Dorner S. Priemer M. (2021). Pitfalls in HLA ligandomics–how to catch a li(e)gand. Mol. and Cell. Proteomics 20, 100110. 10.1016/j.mcpro.2021.100110 PMC8313844 34129939

[B43] Fumagalli M. Rossiello F. Clerici M. Barozzi S. Cittaro D. Kaplunov J. M. (2012). Telomeric DNA damage is irreparable and causes persistent DNA-damage-response activation. Nat. Cell Biol. 14, 355–365. 10.1038/ncb2466 22426077 PMC3717580

[B44] Gfeller D. Schmidt J. Croce G. Guillaume P. Bobisse S. Genolet R. (2023). Improved predictions of antigen presentation and TCR recognition with MixMHCpred2.2 and PRIME2.0 reveal potent SARS-CoV-2 CD8^+^ T-cell epitopes. Cell Syst. 14, 72–83.e5. 10.1016/j.cels.2022.12.002 PMC9811684 36603583

[B45] Giesen C. Wang H. A. O. Schapiro D. Zivanovic N. Jacobs A. Hattendorf B. (2014). Highly multiplexed imaging of tumor tissues with subcellular resolution by mass cytometry. Nat. Methods 11, 417–422. 10.1038/nmeth.2869 24584193

[B46] Glück S. Guey B. Gulen M. F. Wolter K. Kang T. W. Schmacke N. A. (2017). Innate immune sensing of cytosolic chromatin fragments through cGAS promotes senescence. Nat. Cell Biol. 19, 1061–1070. 10.1038/ncb3586 28759028 PMC5826565

[B47] Gonzalez-Galarza F. F. McCabe A. Santos E. J. M. D. Jones J. Takeshita L. Ortega-Rivera N. D. (2020). Allele frequency net database (AFND) 2020 update: gold-standard data classification, open access genotype data and new query tools. Nucleic Acids Res. 48, D783–D788. 10.1093/nar/gkz1029 31722398 PMC7145554

[B48] Gorgoulis V. Adams P. D. Alimonti A. Bennett D. C. Bischof O. Bishop C. (2019). Cellular senescence: defining a path forward. Cell 179, 813–827. 10.1016/j.cell.2019.10.005 31675495

[B49] Goyer M. L. Sonn A. Benabdallah B. Le O. Dulong J. Oschwald J. (2026). The immunogenicity of human senescent cells is dependent on the senescence inducer and cell type. Aging Cell 25 (2), e70410. 10.1111/acel.70410 PMC12900895 41681034

[B50] Grant M. J. Booth A. (2009). A typology of reviews: an analysis of 14 review types and associated methodologies. Health Inf. Libr. J. 26, 91–108. 10.1111/j.1471-1842.2009.00848.x 19490148

[B51] Grosse L. Wagner N. Emelyanov A. Molina C. Lacas-Gervais S. Wagner K. D. (2020). Defined p16^High^ senescent cell types are indispensable for mouse healthspan. Cell Metab. 32, 87–99.e6. 10.1016/j.cmet.2020.05.002 32485135

[B52] Gurkar A. U. Gerencser A. A. Mora A. L. Witkowski J. M. Passos J. F. Sedivy J. M. (2023). Spatial mapping of cellular senescence: emerging challenges and opportunities. Nat. Aging 3, 776–790. 10.1038/s43587-023-00446-6 PMC10505496 37400722

[B53] Gut G. Herrmann M. D. Pelkmans L. (2018). Multiplexed protein maps link subcellular organization to cellular states. Science 361, eaar7042. 10.1126/science.aar7042 30072512

[B54] Guyatt G. H. Oxman A. D. Vist G. E. Kunz R. Falck-Ytter Y. Alonso-Coello P. (2008). GRADE: an emerging consensus on rating quality of evidence and strength of recommendations. BMJ 336, 924–926. 10.1136/bmj.39489.470347 PMC2335261 18436948

[B55] Hao Y. Hao S. Andersen-Nissen E. Mauck W. M. Zheng S. Butler A. (2021). Integrated analysis of multimodal single-cell data. Cell 184, 3573–3587.e29. 10.1016/j.cell.2021.04.048 PMC8238499 34062119

[B56] Harley C. B. Futcher A. B. Greider C. W. (1990). Telomeres shorten during ageing of human fibroblasts. Nature 345, 458–460. 10.1038/345458a0 2342578

[B57] Herbig U. Jobling W. A. Chen B. P. C. Chen D. J. Sedivy J. M. (2004). Telomere shortening triggers senescence of human cells through a pathway involving ATM, p53, and p21^CIP1^, but not p16^INK4a^ . Mol. Cell 14, 501–513. 10.1016/s1097-2765(04)00256-4 15149599

[B58] Hernandez-Segura A. de Jong T. V. Melov S. Guryev V. Campisi J. Demaria M. (2017). Unmasking transcriptional heterogeneity in senescent cells. Curr. Biol. 27, 2652–2660.e4. 10.1016/j.cub.2017.07.033 28844647 PMC5788810

[B59] Hewitt G. Jurk D. Marques F. D. M. Correia-Melo C. Hardy T. Gackowska A. (2012). Telomeres are favoured targets of a persistent DNA-damage response in ageing and stress-induced senescence. Nat. Commun. 3, 708. 10.1038/ncomms1708 22426229 PMC3292717

[B60] Hu Z. Leet D. E. Allesøe R. L. Oliveira G. Li S. Luoma A. M. (2021). Personal neoantigen vaccines induce persistent memory T-cell responses and epitope spreading in patients with melanoma. Nat. Med. 27, 515–525. 10.1038/s41591-020-01206-4 PMC8273876 33479501

[B61] Ichim T. E. Lopes G. Reznik R. Bykoriz V. Fortunati C. A. Pascual K. A. (2025). Reduction of solid tumors by senescent cell immunization. J. Transl. Med. 23, 1365. 10.1186/s12967-025-07393-3 PMC12661736 41316207

[B62] Iltis C. Moskalevska I. Debiesse A. Seguin L. Fissoun C. Cervera L. (2025). A ganglioside-based immune checkpoint enables senescent cells to evade immunosurveillance during aging. Nat. Aging 5, 219–236. 10.1038/s43587-024-00776-z PMC11839482 39730825

[B63] Jacobs J. J. L. de Lange T. (2004). Significant role for p16^INK4a^ in p53-independent telomere-directed senescence. Curr. Biol. 14, 2302–2308. 10.1016/j.cub.2004.12.025 15620660

[B64] Justice J. N. Ferrucci L. Newman A. B. Aroda V. R. Bahnson J. L. Divers J. (2018). A framework for selection of blood-based biomarkers for geroscience-guided clinical trials. GeroScience 40, 419–436. 10.1007/s11357-018-0042-y 30151729 PMC6294728

[B65] Karlseder J. Smogorzewska A. de Lange T. (2002). Senescence induced by altered telomere state, not telomere loss. Science 295, 2446–2449. 10.1126/science.1069523 11923537

[B66] Kim N. W. Piatyszek M. A. Prowse K. R. Harley C. B. West M. D. Ho P. L. C. (1994). Specific association of human telomerase activity with immortal cells and cancer. Science 266, 2011–2015. 10.1126/science.7605428 7605428

[B67] Kirkland J. L. Tchkonia T. (2020). Senolytic drugs: from discovery to translation. J. Intern. Med. 288, 518–536. 10.1111/joim.13141 32686219 PMC7405395

[B68] Kranz L. M. Diken M. Haas H. Kreiter S. Loquai C. Reuter K. C. (2016). Systemic RNA delivery to dendritic cells exploits antiviral defence for cancer immunotherapy. Nature 534, 396–401. 10.1038/nature18300 27281205

[B69] Kreiter S. Vormehr M. van de Roemer N. Diken M. Löwer M. Diekmann J. (2015). Mutant MHC class II epitopes drive therapeutic immune responses to cancer. Nature 520, 692–696. 10.1038/nature14426 25901682 PMC4838069

[B70] Krizhanovsky V. Yon M. Dickins R. A. Hearn S. Simon J. Miething C. (2008). Senescence of activated stellate cells limits liver fibrosis. Cell 134, 657–667. 10.1016/j.cell.2008.06.049 18724938 PMC3073300

[B71] Lai T. P. Zhang N. Noh J. Mender I. Tedone E. Huang E. (2017). A method for measuring the distribution of the shortest telomeres in cells and tissues. Nat. Commun. 8, 1356. 10.1038/s41467-017-01291-z 29116081 PMC5676791

[B72] Landis J. R. Koch G. G. (1977). The measurement of observer agreement for categorical data. Biometrics 33, 159–174. 10.2307/2529310 843571

[B73] Lansdorp P. M. Verwoerd N. P. van de Rijke F. M. Dragowska V. Little M. T. Dirks R. W. (1996). Heterogeneity in telomere length of human chromosomes. Hum. Mol. Genet. 5, 685–691. 10.1093/hmg/5.5.685 8733138

[B74] Liu G. Carter B. Bricken T. Jain S. Viard M. Carrington M. (2020). Computationally optimized SARS-CoV-2 MHC class I and II vaccine formulations predicted to target human haplotype distributions. Cell Syst. 11, 131–144.e6. 10.1016/j.cels.2020.06.009 32721383 PMC7384425

[B75] Liu D. X. Xu Y. H. Qian C. (2024). “Peptide vaccine design by evolutionary multi-objective optimization,” in In Proceedings of the Thirty-Third International Joint Conference on Artificial Intelligence, 6967–6975. 10.24963/ijcai.2024/770

[B76] Love M. I. Huber W. Anders S. (2014). Moderated estimation of fold change and dispersion for RNA-seq data with DESeq2. Genome Biol. 15, 550. 10.1186/s13059-014-0550-8 25516281 PMC4302049

[B77] Luecken M. D. Theis F. J. (2019). Current best practices in single-cell RNA-seq analysis: a tutorial. Mol. Syst. Biol. 15, e8746. 10.15252/msb.20188746 31217225 PMC6582955

[B78] Luecken M. D. Büttner M. Chaichoompu K. Danese A. Interlandi M. Müller M. F. (2022). Benchmarking atlas-level data integration in single-cell genomics. Nat. Methods 19, 41–50. 10.1038/s41592-021-01336-8 PMC8748196 34949812

[B79] Majewska J. Agrawal A. Mayo A. Roitman L. Chatterjee R. Sekeresova Kralova J. (2024). p16-dependent increase of PD-L1 stability regulates immunosurveillance of senescent cells. Nat. Cell Biol. 26, 1336–1345. 10.1038/s41556-024-01465-0 PMC11321988 39103548

[B80] Majzner R. G. Rietberg S. P. Sotillo E. Dong R. Vachharajani V. T. Labanieh L. (2020). Tuning the antigen density requirement for CAR T-cell activity. Cancer Discov. 10, 702–723. 10.1158/2159-8290.CD-19-0945 32193224 PMC7939454

[B82] Mender I. Shay J. W. (2015). Telomere restriction fragment analysis. Bio-protocol 5, e1658. 10.21769/bioprotoc.1658 27500189 PMC4972328

[B83] Miake-Lye I. M. Hempel S. Shanman R. Shekelle P. G. (2016). What is an evidence map? A systematic review of published evidence maps and their definitions, methods, and products. Syst. Rev. 5, 28. 10.1186/s13643-016-0204-x 26864942 PMC4750281

[B84] Muñoz D. P. Yannone S. M. Daemen A. Sun Y. Vakar-Lopez F. Kawahara M. (2019). Targetable mechanisms driving immunoevasion of persistent senescent cells link chemotherapy-resistant cancer to aging. JCI Insight 4, e124716. 10.1172/jci.insight.124716 31184599 PMC6675550

[B85] Muñoz-Espín D. Cañamero M. Maraver A. Gómez-López G. Contreras J. Murillo-Cuesta S. (2013). Programmed cell senescence during mammalian embryonic development. Cell 155, 1104–1118. 10.1016/j.cell.2013.10.019 24238962

[B86] Murphy A. E. Skene N. G. (2022). A balanced measure shows superior performance of pseudobulk methods in single-cell RNA-sequencing analysis. Nat. Commun. 13, 7851. 10.1038/s41467-022-35519-4 PMC9780232 36550119

[B87] National Institutes of Health (2019). Inclusion Across the Lifespan in Human Subjects Research. Available online at: https://grants.nih.gov/policy-and-compliance/policy-topics/inclusion/lifespan (Accessed July 16, 2026).

[B88] Nelson G. Wordsworth J. Wang C. Jurk D. Lawless C. Martin-Ruiz C. (2012). A senescent cell bystander effect: senescence-induced senescence. Aging Cell 11, 345–349. 10.1111/j.1474-9726.2012.00795.x 22321662 PMC3488292

[B89] O'Donnell T. J. Rubinsteyn A. Laserson U. (2020). MHCflurry 2.0: improved pan-allele prediction of MHC class I-presented peptides by incorporating antigen processing. Cell Syst. 11, 42–48.e7. 10.1016/j.cels.2020.06.010 32711842

[B90] Ogrodnik M. Carlos Acosta J. Adams P. D. d'Adda di Fagagna F. Baker D. J. Bishop C. L. (2024). Guidelines for minimal information on cellular senescence experimentation *in vivo* . Cell 187, 4150–4175. 10.1016/j.cell.2024.05.059 PMC11790242 39121846

[B91] Oppezzo A. Sepe S. Cicio G. Cancila V. Sasso E. Conti A. (2026). Therapeutic inhibition of telomeric DNA damage response rescues hematopoietic dysfunction driven by telomere shortening and aging. Nat. Aging 6, 1377–1394. 10.1038/s43587-026-01136-9 PMC13375536 42380622

[B92] Ott P. A. Hu Z. Keskin D. B. Shukla S. A. Sun J. Bozym D. J. (2017). An immunogenic personal neoantigen vaccine for patients with melanoma. Nature 547, 217–221. 10.1038/nature22991 28678778 PMC5577644

[B93] Ovadya Y. Landsberger T. Leins H. Vadai E. Gal H. Biran A. (2018). Impaired immune surveillance accelerates accumulation of senescent cells and aging. Nat. Commun. 9, 5435. 10.1038/s41467-018-07825-3 30575733 PMC6303397

[B94] Page M. J. McKenzie J. E. Bossuyt P. M. Boutron I. Hoffmann T. C. Mulrow C. D. (2021). The PRISMA 2020 statement: an updated guideline for reporting systematic reviews. BMJ 372, n71. 10.1136/bmj.n71 PMC8005924 33782057

[B95] Passos J. F. Nelson G. Wang C. Richter T. Simillion C. Proctor C. J. (2010). Feedback between p21 and reactive oxygen production is necessary for cell senescence. Mol. Syst. Biol. 6, 347. 10.1038/msb.2010.5 20160708 PMC2835567

[B96] Percie du Sert N. Hurst V. Ahluwalia A. Alam S. Avey M. T. Baker M. (2020). The ARRIVE guidelines 2.0: updated guidelines for reporting animal research. PLoS Biol. 18, e3000410. 10.1371/journal.pbio.3000410 32663219 PMC7360023

[B97] Pereira B. I. Devine O. P. Vukmanovic-Stejic M. Chambers E. S. Subramanian P. Patel N. (2019). Senescent cells evade immune clearance via HLA-E-mediated NK and CD8^+^ T-cell inhibition. Nat. Commun. 10, 2387. 10.1038/s41467-019-10335-5 31160572 PMC6547655

[B98] Perez-Riverol Y. Bandla C. Kundu D. J. Kamatchinathan S. Bai J. Hewapathirana S. (2025). The PRIDE database at 20 years: 2025 update. Nucleic Acids Res. 53 (D1), D543–D553. 10.1093/nar/gkae1011 PMC11701690 39494541

[B99] Piedra-Quintero Z. L. Wilson Z. Nava P. Guerau-de-Arellano M. (2020). CD38: an immunomodulatory molecule in inflammation and autoimmunity. Front. Immunol. 11, 597959. 10.3389/fimmu.2020.597959 PMC7734206 33329591

[B100] Purcell A. W. Ramarathinam S. H. Ternette N. (2019). Mass-spectrometry-based identification of MHC-bound peptides for immunopeptidomics. Nat. Protoc. 14, 1687–1707. 10.1038/s41596-019-0133-y 31092913

[B101] Rethlefsen M. L. Kirtley S. Waffenschmidt S. Ayala A. P. Moher D. Page M. J. (2021). PRISMA-S: an extension to the PRISMA statement for reporting literature searches in systematic reviews. J. Med. Libr. Assoc. 109, 174–200. 10.5195/jmla.2021.962 PMC8270366 34285662

[B102] Reynisson B. Alvarez B. Paul S. Peters B. Nielsen M. (2020). NetMHCpan-4.1 and NetMHCIIpan-4.0: improved predictions of MHC antigen presentation by concurrent motif deconvolution and integration of mass-spectrometry MHC-eluted ligand data. Nucleic Acids Res. 48, W449–W454. 10.1093/nar/gkaa379 PMC7319546 32406916

[B103] Richman L. P. Vonderheide R. H. Rech A. J. (2019). Neoantigen dissimilarity to the self-proteome predicts immunogenicity and response to immune checkpoint blockade. Cell Syst. 9, 375–382.e4. 10.1016/j.cels.2019.08.009 31606370 PMC6813910

[B104] Rojas L. A. Sethna Z. Soares K. C. Olcese C. Pang N. Patterson E. (2023). Personalized RNA neoantigen vaccines stimulate T cells in pancreatic cancer. Nature 618, 144–150. 10.1038/s41586-023-06063-y PMC10171177 37165196

[B105] Rolland Y. Sierra F. Ferrucci L. Barzilai N. De Cabo R. Mannick J. (2023). Challenges in developing geroscience trials. Nat. Commun. 14, 5038. 10.1038/s41467-023-39786-7 PMC10439920 37598227

[B106] Rossiello F. Jurk D. Passos J. F. d'Adda di Fagagna F. (2022). Telomere dysfunction in ageing and age-related diseases. Nat. Cell Biol. 24, 135–147. 10.1038/s41556-022-00842-x PMC8985209 35165420

[B107] Roybal K. T. Rupp L. J. Morsut L. Walker W. J. McNally K. A. Park J. S. (2016). Precision tumor recognition by T cells with combinatorial antigen-sensing circuits. Cell 164, 770–779. 10.1016/j.cell.2016.01.011 26830879 PMC4752902

[B108] Rufer N. Brümmendorf T. H. Kolvraa S. Bischoff C. Christensen K. Wadsworth L. (1998). Telomere length dynamics in human lymphocyte subpopulations measured by flow cytometry. Nat. Biotechnol. 16, 743–747. 10.1038/nbt0898-743 9702772

[B109] Sagiv A. Biran A. Yon M. Simon J. Lowe S. W. Krizhanovsky V. (2013). Granule exocytosis mediates immune surveillance of senescent cells. Oncogene 32, 1971–1977. 10.1038/onc.2012.206 22751116 PMC3630483

[B110] Sahin U. Derhovanessian E. Miller M. Kloke B. P. Simon P. Löwer M. (2017). Personalized RNA mutanome vaccines mobilize poly-specific therapeutic immunity against cancer. Nature 547, 222–226. 10.1038/nature23003 28678784

[B111] Sanborn M. A. Wang X. Gao S. Dai Y. Rehman J. (2025). Unveiling the cell-type-specific landscape of cellular senescence through single-cell transcriptomics using SenePy. Nat. Commun. 16, 1884. 10.1038/s41467-025-57047-7 PMC11846890 39987255

[B112] Sarkizova S. Klaeger S. Le P. M. Li L. W. Oliveira G. Keshishian H. (2020). A large peptidome dataset improves HLA class I epitope prediction across most of the human population. Nat. Biotechnol. 38, 199–209. 10.1038/s41587-019-0322-9 31844290 PMC7008090

[B113] Saul D. Kosinsky R. L. Atkinson E. J. Doolittle M. L. Zhang X. LeBrasseur N. K. (2022). A new gene set identifies senescent cells and predicts senescence-associated pathways across tissues. Nat. Commun. 13, 4827. 10.1038/s41467-022-32552-1 PMC9381717 35974106

[B114] Sethna Z. Guasp P. Reiche C. Milighetti M. Ceglia N. Patterson E. (2025). RNA neoantigen vaccines prime long-lived CD8^+^ T cells in pancreatic cancer. Nature 639, 1042–1051. 10.1038/s41586-024-08508-4 PMC11946889 39972124

[B115] Sfeir A. de Lange T. (2012). Removal of shelterin reveals the telomere end-protection problem. Science 336, 593–597. 10.1126/science.1218498 22556254 PMC3477646

[B116] Siametis A. Stratigi K. Giamaki D. Chatzinikolaou G. Akalestou-Clocher A. Goulielmaki E. (2024). Transcription stress at telomeres leads to cytosolic DNA release and paracrine senescence. Nat. Commun. 15, 4061. 10.1038/s41467-024-48443-6 PMC11094137 38744897

[B117] Snyder H. (2019). Literature review as a research methodology: an overview and guidelines. J. Bus. Res. 104, 333–339. 10.1016/j.jbusres.2019.07.039

[B118] Squair J. W. Gautier M. Kathe C. Anderson M. A. James N. D. Hutson T. H. (2021). Confronting false discoveries in single-cell differential expression. Nat. Commun. 12, 5692. 10.1038/s41467-021-25960-2 PMC8479118 34584091

[B119] Srivastava S. Salter A. I. Liggitt D. Yechan-Gunja S. Sarvothama M. Cooper K. (2019). Logic-gated ROR1 chimeric antigen receptor expression rescues T cell-mediated toxicity to normal tissues and enables selective tumor targeting. Cancer Cell 35, 489–503.e8. 10.1016/j.ccell.2019.02.003 30889382 PMC6450658

[B120] Stoeckius M. Hafemeister C. Stephenson W. Houck-Loomis B. Chattopadhyay P. K. Swerdlow H. (2017). Simultaneous epitope and transcriptome measurement in single cells. Nat. Methods 14, 865–868. 10.1038/nmeth.4380 28759029 PMC5669064

[B121] Storer M. Mas A. Robert-Moreno A. Pecoraro M. Ortells M. C. Di Giacomo V. (2013). Senescence is a developmental mechanism that contributes to embryonic growth and patterning. Cell 155, 1119–1130. 10.1016/j.cell.2013.10.041 24238961

[B122] Suda M. Shimizu I. Katsuumi G. Yoshida Y. Hayashi Y. Ikegami R. (2021). Senolytic vaccination improves normal and pathological age-related phenotypes and increases lifespan in progeroid mice. Nat. Aging 1, 1117–1126. 10.1038/s43587-021-00151-2 37117524

[B123] Suda M. Shimizu I. Katsuumi G. Hsiao C. L. Yoshida Y. Matsumoto N. (2022). Glycoprotein nonmetastatic melanoma protein B regulates lysosomal integrity and lifespan of senescent cells. Sci. Rep. 12, 6522. 10.1038/s41598-022-10522-3 PMC9021310 35444208

[B124] Suram A. Kaplunov J. Patel P. L. Ruan H. Cerutti A. Boccardi V. (2012). Oncogene-induced telomere dysfunction enforces cellular senescence in human cancer precursor lesions. EMBO J. 31, 2839–2851. 10.1038/emboj.2012.132 22569128 PMC3395091

[B125] Suryadevara V. Hudgins A. D. Rajesh A. Pappalardo A. Karpova A. Dey A. K. (2024). SenNet recommendations for detecting senescent cells in different tissues. Nat. Rev. Mol. Cell Biol. 25, 1001–1023. 10.1038/s41580-024-00738-8 PMC11578798 38831121

[B126] Suryadevara V. Farzad N. Yang M. Xu K. Tsankov A. Thompson R. C. (2026). Charting human cellular senescence in aging and disease. Cell 189 (12), 3501–3505. 10.1016/j.cell.2026.05.028 42276030

[B127] Sykulev Y. Joo M. Vturina I. Tsomides T. J. Eisen H. N. (1996). Evidence that a single peptide-MHC complex on a target cell can elicit a cytolytic T cell response. Immunity 4, 565–571. 10.1016/S1074-7613(00)80483-5 8673703

[B128] Takai H. Smogorzewska A. de Lange T. (2003). DNA damage foci at dysfunctional telomeres. Curr. Biol. 13, 1549–1556. 10.1016/s0960-9822(03)00542-6 12956959

[B129] Tang Y. Lou J. Alpaugh R. K. Robinson M. K. Marks J. D. Weiner L. M. (2007). Regulation of antibody-dependent cellular cytotoxicity by IgG intrinsic and apparent affinity for target antigen. J. Immunol. 179, 2815–2823. 10.4049/jimmunol.179.5.2815 17709495

[B130] Tousley A. M. Rotiroti M. C. Labanieh L. Rysavy L. W. Kim W. J. Lareau C. (2023). Co-opting signalling molecules enables logic-gated control of CAR T cells. Nature 615, 507–516. 10.1038/s41586-023-05778-2 PMC10564584 36890224

[B131] Toussaint N. C. Dönnes P. Kohlbacher O. (2008). A mathematical framework for the selection of an optimal set of peptides for epitope-based vaccines. PLoS Comput. Biol. 4, e1000246. 10.1371/journal.pcbi.1000246 19112482 PMC2588662

[B132] Uhlén M. Fagerberg L. Hallström B. M. Lindskog C. Oksvold P. Mardinoglu A. (2015). Tissue-based map of the human proteome. Science 347, 1260419. 10.1126/science.1260419 25613900

[B134] Watanabe K. Terakura S. Martens A. C. van Meerten T. Uchiyama S. Imai M. (2015). Target antigen density governs the efficacy of anti-CD20-CD28-CD3 zeta chimeric antigen receptor-modified effector CD8+ T cells. J. Immunol. 194, 911–920. 10.4049/jimmunol.1402346 25520398

[B135] Wells D. K. van Buuren M. M. Dang K. K. Hubbard-Lucey V. M. Sheehan K. C. F. Campbell K. M. (2020). Key parameters of tumour epitope immunogenicity revealed through a consortium approach improve neoantigen prediction. Cell 183, 818–834.e13. 10.1016/j.cell.2020.09.015 PMC7652061 33038342

[B136] Wilhelm M. Zolg D. P. Graber M. Gessulat S. Schmidt T. Schnatbaum K. (2021). Deep learning boosts sensitivity of mass-spectrometry-based immunopeptidomics. Nat. Commun. 12, 3346. 10.1038/s41467-021-23713-9 PMC8184761 34099720

[B137] Wilkinson M. D. Dumontier M. Aalbersberg I. J. Appleton G. Axton M. Baak A. (2016). The FAIR guiding principles for scientific data management and stewardship. Sci. Data 3, 160018. 10.1038/sdata.2016.18 26978244 PMC4792175

[B138] Wollscheid B. Bausch-Fluck D. Henderson C. O'Brien R. Bibel M. Schiess R. (2009). Mass-spectrometric identification and relative quantification of N-linked cell-surface glycoproteins. Nat. Biotechnol. 27, 378–386. 10.1038/nbt.1532 19349973 PMC2829300

[B139] World Medical Association (2025). World medical association declaration of Helsinki: ethical principles for medical research involving human participants. JAMA 333 (1), 71–74. 10.1001/jama.2024.21972 39425955

[B140] Xu M. Pirtskhalava T. Farr J. N. Weigand B. M. Palmer A. K. Weivoda M. M. (2018). Senolytics improve physical function and increase lifespan in old age. Nat. Med. 24, 1246–1256. 10.1038/s41591-018-0092-9 29988130 PMC6082705

[B141] Yang D. Sun B. Li S. Wei W. Liu X. Cui X. (2023). NKG2D-CAR T cells eliminate senescent cells in aged mice and nonhuman primates. Sci. Transl. Med. 15, eadd1951. 10.1126/scitranslmed.add1951 37585504

[B142] Yoshida S. Nakagami H. Hayashi H. Ikeda Y. Sun J. Tenma A. (2020). The CD153 vaccine is a senotherapeutic option for preventing the accumulation of senescent T cells in mice. Nat. Commun. 11, 2482. 10.1038/s41467-020-16347-w 32424156 PMC7235045

[B143] Yu S. Li Z. Tang Y. Chen Y. Ma Y. Du K. (2025). CD38-targeting peptide vaccine ameliorates aging-associated phenotypes in mice. Aging Cell 24 (9), e70147. 10.1111/acel.70147 PMC12419845 40557469

[B144] Zeng H. Gifford D. K. (2019). Quantification of uncertainty in peptide–MHC binding prediction improves high-affinity peptide selection for therapeutic design. Cell Syst. 9, 159–166.e3. 10.1016/j.cels.2019.05.004 31176619 PMC6715517

[B145] Zhang K. Yin Q. Ma Y. Cao M. Li L. Jin X. (2025). Nanovaccine loaded with seno-antigen target senescent cells to improve metabolic disorders of adipose tissue and cardiac dysfunction. Hum. Vaccines and Immunother. 21, 2479229. 10.1080/21645515.2025.2479229 PMC11916409 40088037

[B146] Zhao H. Liu Z. Chen H. Han M. Zhang M. Liu K. (2024). Identifying specific functional roles for senescence across cell types. Cell 187, 7314–7334.e21. 10.1016/j.cell.2024.09.021 39368477

[B147] Zhu Y. Tchkonia T. Pirtskhalava T. Gower A. C. Ding H. Giorgadze N. (2015). The Achilles' heel of senescent cells: from transcriptome to senolytic drugs. Aging Cell 14, 644–658. 10.1111/acel.12344 25754370 PMC4531078

[B148] Zimmerman K. D. Espeland M. A. Langefeld C. D. (2021). A practical solution to pseudoreplication bias in single-cell studies. Nat. Commun. 12, 738. 10.1038/s41467-021-21038-1 33531494 PMC7854630

