# Supplementary material for: From telomere dysfunction to seno-antigen selection: a conceptual framework and route-specific evidence appraisal for senolytic immunotherapy
Source: Front Syst Biol. 2026 Oct 27;6:1940319. doi: 10.3389/fsysb.2026.1940319 (PMC13748548; doi:10.3389/fsysb.2026.1940319)
Supplement: Supplementary file 1 [file DataSheet1.pdf]

# Supplementary Methods

*From Telomere Dysfunction to Seno-Antigen Selection: A Conceptual Framework and Route-Specific Evidence Appraisal for Senolytic Immunotherapy*

This document supports the Methods section. It records the review type and its limitations, the representative search strategy and its block-combination logic, the eligibility rules applied, the handling of duplicates, overlapping datasets, and contradictory evidence, a SANRA self-assessment, the grading rubric, and a candidate-evidence log for the studies appraised in Tables 3 and 10 of the main article. Because this is a single-author conceptual narrative review rather than a systematic review, formal PRISMA screening counts and a validated flow diagram are not claimed, and the strings below are a representative strategy rather than exact executable queries validated for each database; the material documents how the literature was identified and appraised so that the synthesis can be audited, not exactly reproduced.

## 1. Review type, authorship, and limitations

This is a single-author structured narrative review with a conceptual-framework component. Searching, screening, extraction, interpretation, and candidate grading were performed by one investigator, without second-reviewer adjudication. This introduces risks of selective retrieval and interpretation that documentation reduces but cannot eliminate, and independent verification of the search, the appraisal in Table 3, and the grading in Table 10 is recommended before the framework informs any experimental or clinical decision.

## 2. Information sources and final search date

PubMed (NCBI), Embase (Elsevier), Scopus (Elsevier), and Web of Science Core Collection (Clarivate) were searched from database inception to 15 July 2026. Searches were restricted to English-language, peer-reviewed articles and recognized preprints. Backward and forward citation searching was performed on key primary studies and reviews, and primary resource and dataset publications (for example atlases, immunopeptidome repositories, and allele-frequency databases) were consulted directly.

## 3. Concept blocks and representative search strings

This was a targeted narrative search rather than a systematic search, so the strings below are the representative strategy used to identify evidence, not exhaustive executable queries validated for completeness in each database. Terms were combined within a block with OR. The core of every search combined the senescence-phenotype block (B1) with the immune-accessibility block (B3) using AND. The telomere (B2), multi-omics (B4), safety (B5), and senolysis (B6) blocks were then added one or two at a time as optional expanders, using AND only to focus a specific sub-question, and were never all required simultaneously; requiring every block at once would have excluded the non-telomeric and non-bioinformatic evidence the framework depends on. Candidate-specific searches were run separately for each named antigen (B-cand below) combined with B1 and B5. Platform-specific syntax (Emtree, Scopus field codes, Web of Science topic fields) was adapted from the PubMed-style strings shown.

```
B1 Senescence phenotype: ("cellular senescence"[tiab] OR senescent[tiab] OR SASP[tiab] OR "senescence-associated"[tiab] OR p16[tiab] OR p21[tiab])
```

```
B2 Telomere dysfunction: (telomere*[tiab] OR shelterin[tiab] OR "telomere dysfunction"[tiab] OR TIF[tiab] OR "telomere-associated foci"[tiab] OR TERRA[tiab])
```

```
B3 Immune accessibility / presentation: (surfaceome[tiab] OR "cell surface"[tiab] OR immunopeptidom*[tiab] OR "HLA ligand*"[tiab] OR "antigen presentation"[tiab] OR "eluted ligand*"[tiab])

B4 Bioinformatics / multi-omics: ("single-cell"[tiab] OR scRNA*[tiab] OR spatial[tiab] OR proteomic*[tiab] OR "epitope prediction"[tiab] OR NetMHC*[tiab] OR surfaceomic*[tiab])

B5 Selectivity / safety: (selectiv*[tiab] OR "off-target"[tiab] OR "on-target off-tumor"[tiab] OR tolerance[tiab] OR autoimmun*[tiab] OR "healthy tissue"[tiab])

B6 Immune senolysis / translation: (senolytic[tiab] OR "senolytic vaccine"[tiab] OR CAR-T[tiab] OR "chimeric antigen receptor"[tiab] OR "senescence vaccine"[tiab] OR immunosurveillance[tiab])

B-cand Candidate-specific (example): ("GPNMB"[tiab] OR "glycoprotein nonmetastatic melanoma protein B"[tiab]) AND (senescen*[tiab] OR senolytic[tiab]) AND (vaccine[tiab] OR antibody[tiab] OR "cell surface"[tiab] OR safety[tiab])
```

Candidate-specific searches were run separately for each named antigen appraised in the main text (for example uPAR/PLAUR, GPNMB, CD153, CD38, and NKG2D ligands), combined with the senescence-phenotype block (B1) and the safety block (B5), as shown in B-cand.

## 4. Eligibility criteria

Included: primary experimental studies, method and resource papers, and reviews addressing senescence-associated antigen identification, immune recognition or clearance of senescent cells, telomere dysfunction relevant to senescence, or computational methods for antigen and epitope prioritization. Excluded: records without senescence relevance; studies with no accessible antigen, immune-recognition, or safety content; conference abstracts lacking primary data; and non-English records. Where multiple reports analyzed overlapping samples or datasets, they were linked and treated as a single body of evidence so that reanalysis was not counted as independent replication. Recognized preprints were eligible; the publication status of every cited item was checked, and the studies underlying the candidate appraisal in Tables 3 and 10 are peer-reviewed journal articles (for example Amor et al., 2020, Nature; Suda et al., 2021, Nature Aging; Yoshida et al., 2020, Nature Communications). Any item that remains a preprint at submission is labeled as such with its server and posting date in the main reference list.

## 5. Handling of duplicates, overlap, and contradictory evidence

Records were de-duplicated across databases by digital object identifier and title. Overlapping datasets were identified by matching cohorts, cell sources, and accession identifiers, and were annotated in the candidate-evidence log. Contradictory findings were sought explicitly by pairing each positive claim (for example senescence enrichment or antigen-dependent killing) with searches for negative controls, antigen-negative or knockout experiments, and normal-tissue expression, and were retained in the synthesis because they reveal cell-type restriction, inducer dependence, or lack of reproducibility.

## 6. SANRA self-assessment

The Scale for the Assessment of Narrative Review Articles (SANRA) was applied by the author as a drafting self-audit. Each item is scored 0 to 2.

| SANRA item | Score | Basis |
|---|---|---|
| Justification of importance | 2 | The clinical rationale for selective senescent-cell clearance and the specificity problem are established with primary evidence. |

| SANRA item | Score | Basis |
|---|---|---|
| Statement of aims | 2 | The aim, a route-aware conceptual framework and evidence appraisal, is stated explicitly and distinguished from a validated pipeline. |
| Description of literature search | 2 | Databases, dates, restrictions, concept blocks, and representative strings are provided here. |
| Referencing | 2 | Claims are tied to primary sources; contradictory evidence is cited. |
| Scientific reasoning | 2 | Evidence is graded by directness and route; nomination is separated from validation. |
| Data presentation | 2 | Appraisal, rubric, and worked-example cards are tabulated with explicit grades including untested. |

Total self-assessed score: 12 of 12. Self-assessment by a single author is not a substitute for independent appraisal.

## 7. Grading rubric (reproduces Table 6)

The rubric below defines each grade for the four compulsory criteria and the optional telomere axis, and is reproduced from the main article for completeness.

| Criterion | Met (minimum evidence) | Partial | Not met | Untested / hard stop |
|---|---|---|---|---|
| Persistent senescence | Durable proliferative arrest where arrest is biologically assessable, or, in postmitotic lineages, lineage-appropriate evidence of a persistent pathological senescent state, plus ≥2 orthogonal senescence features in the target cells in the disease context | One orthogonal feature only, arrest of uncertain durability, or a single-marker call | Phenotype attributable to quiescence, differentiation, or another non-senescent state | Untested if multi-feature data are absent; conflicting findings are not merged into one grade but yield a context-stratified or lower-certainty grade |
| Immune accessibility | Direct detection on intact cells (surface) or eluted HLA ligand with FDR control | Direct total-protein evidence without intact-cell surface localization | Intracellular-only or shed-decoy evidence | mRNA or transcript-only evidence, or no intact-cell surface or eluted-ligand assay performed |
| Functional recognition | Route-specific antigen-dependent recognition and senolytic clearance in the intended modality (killing, complement, antibody-dependent cellular cytotoxicity, phagocytic clearance, natural-killer, or checkpoint de-repression), confirmed by antigen blocking, knockout, or antigen-negative controls (activity is lost when the antigen is removed) | Recognition or clearance in the intended modality but without antigen-dependence controls (uncontrolled recognition) | No clearance despite an adequate route-appropriate assay in the intended modality | Untested if not assayed in the intended modality; a positive result from another route (for example CAR-T for a vaccine) is nomination only and is graded untested |
| Healthy-state exclusion | A prespecified route-specific safety margin is met across essential, activated, progenitor, reparative, and beneficial-senescent cells: target density below the effector-activation threshold, or no antigen-dependent injury at clinically relevant exposure, shown by a defined assay | Margin met in some but not all required healthy compartments, or demonstrated only in vitro | Antigen-dependent injury, or expression at or above the activation threshold on an indispensable healthy population (HARD STOP) | Untested (default) whenever the prespecified healthy-cell panel and exposure have not been tested; graded untested, not met |
| Telomere axis (optional) | Direct telomere-damage measurement paired with the antigen in the same cells | Telomere data in a related but not matched sample | Antigen enriched in telomere-intact senescence | Untested (default) whenever matched same-cell data are absent; unrelated models do not establish convergence |

## 8. Route-specific evidence requirements (reproduces Table 7)

| Route / product | Recognition mechanism | What functional recognition requires | What transfers to other routes |
|---|---|---|---|
| Antibody / ADC | Intact-surface epitope; Fc or payload effector | Intact-cell binding at adequate density; antigen-dependent killing; healthy-cell sparing | Nominates a surface target for engineered cells; does not establish vaccine tolerance or T-cell immunity |
| Engineered cell (CAR / logic-gated) | Receptor-defined surface antigen; HLA-independent; AND-gating possible | Receptor-dependent lysis with antigen-negative controls; on-target off-tumor mapping | Nominates the surface antigen; does not establish natural HLA presentation, tolerance, or vaccine immunogenicity |
| Native-surface-antigen vaccine | Host antibody and T-cell response to a self surface protein | Overcome tolerance; effective titer or T-cell response; antigen-dependent clearance; healthy sparing | Confirms accessibility of the antigen; does not transfer CAR killing data as vaccine efficacy |
| HLA-restricted peptide vaccine | CD8/CD4 recognition of naturally presented peptides | Eluted human ligands; population coverage; demonstrated human immunogenicity and cytotoxicity | Murine or predicted data nominate; human presentation and immunogenicity remain required |

## 9. Candidate-evidence log

This log records, for the candidates graded in Tables 3 and 10, the study identifier, model and population, the comparator and target-dependence controls, the key reported effects, safety and healthy-state findings, contradictory or limiting evidence, and the precise finding used to justify each grade. Numerical detail, meaning total and per-group sample sizes and principal quantitative effects, is provided for the worked-example candidates (uPAR, GPNMB) and the GPNMB cellular-route variants (Cao, Zhang); the remaining reports appraised in Table 3 are summarized qualitatively, with complete numerical data in the cited primary sources. Where a per-group count appears only within a specific figure of a source, it is cited to that source rather than reproduced in full.

### 9.1 uPAR (PLAUR), engineered-cell (CAR-T) route

**Study identifiers and per-report contribution:** Three reports contribute, and their contributions are distinguished here. (i) Amor et al. (2020) Nature 583:127-132, https://doi.org/10.1038/s41586-020-2403-9 is the primary study providing target discovery, the CAR design and dosing, the senescence characterization, and the in-vivo efficacy and sample sizes described below. (ii) Amor et al. (2024) Nature Aging contributes the finding that low-dose uPAR CAR-T improves metabolic function in aged mice, extending the engineered-cell efficacy evidence to an aging context; its specific cohorts are reported in that paper. (iii) Eskiocak et al. (2026) Nature Aging, https://doi.org/10.1038/s43587-025-01022-w, contributes physiological-challenge evidence for the CAR route: uPAR CAR-T cells were tested in a 15 Gy abdominal-irradiation intestinal-injury model and reported to improve epithelial regeneration, barrier function, and mucosal immune function in aged mice, with its own cohorts distinct from the 2020 figures. All three are peer-reviewed.

**Model and population (Amor 2020):** Target identified by comparing diseased versus normal transcriptomes across three senescence models: therapy-induced (murine KrasG12D;p53-null lung adenocarcinoma after MEK plus CDK4/6 inhibition), oncogene-induced (murine hepatocytes), and culture-induced (hepatic stellate cells). In-vivo tests used immunocompetent C57BL/6J mice with lung adenocarcinoma and with carbon tetrachloride-induced and diet-induced (steatohepatitis) liver fibrosis, and immunodeficient mice for oncogene-induced senescent hepatocytes.

**Comparator and target-dependence controls (Amor 2020):** Anti-uPAR single-chain-variable-fragment CAR with CD28 and CD3-zeta domains, versus untransduced or antigen-negative controls; killing shown to be uPAR-dependent in vitro and in vivo, with antigen-negative cells spared. Dose $3 \times 10^6$ murine CAR T cells

intravenously, 24 h after cyclophosphamide 200 mg/kg; liver-fibrosis arm dosed over a 6-week carbon tetrachloride regimen and assessed at day 20.

**Senescence characterization, persistence evidence (Amor 2020):** uPAR-positive cells were non-proliferative (Ki67-negative), SA-beta-galactosidase-positive, and co-expressed p16 and the SASP factor IL-6, across multiple models; in human hepatitis- and HCV-related liver fibrosis, uPAR co-localized with p16 and with IL-6 by co-immunofluorescence. This provides durable arrest plus at least two orthogonal senescence features in the same cells.

**Key reported effects and sample sizes (Amor 2020):** Human validation: immunohistochemistry n = 7 patients and co-immunofluorescence n = 3 for hepatitis-induced fibrosis, n = 7 and n = 3 for HCV-related residual fibrosis, n = 5 carotid endarterectomy, and n = 3 pancreatic-intraepithelial-neoplasia samples (mean +/- s.e.m.). Mouse efficacy: extended survival in the lung-adenocarcinoma model and reduced established liver fibrosis; senescent cells ablated in vitro and in vivo; no T-cell exhaustion at 15 days; representative of two independent experiments with n = 3 mice per group.

**Safety and healthy-state findings:** uPAR is expressed on injury-responsive myeloid and stromal cells and induced during inflammation and repair; dose-related cytokine toxicity is a recognized CAR-T concern. Relevant physiological-challenge evidence exists for the CAR route: Eskiocak et al. (2026) used a 15 Gy abdominal-irradiation intestinal-injury model and reported improved rather than impaired epithelial regeneration, barrier function, and mucosal immune function after uPAR CAR-T. This is genuine on-target challenge evidence but does not constitute a prespecified safety margin across all required healthy compartments (infection, surgery, other repair), and no route-specific target-density-versus-threshold assay was performed.

**Contradictory or limiting evidence:** All efficacy evidence is in the engineered-cell route; no vaccine experiment against uPAR has been performed, so vaccine functional recognition is untested rather than negative. Injury-associated uPAR expression remains a healthy-state liability; the irradiation-regeneration data mitigate but do not resolve it, since a full multi-compartment safety margin has not been demonstrated.

**Grade justification, CAR route:** Persistent senescence: MET (Ki67-negative, SA-beta-gal-positive, p16- and IL-6-co-expressing across models and in human tissue). Immune accessibility: MET (intact cell-surface uPAR by immunohistochemistry and CAR binding). Functional recognition: MET (receptor-dependent, antigen-negative-controlled killing). Healthy-state exclusion: PARTIAL (irradiation-regeneration challenge shows on-target benefit, but no prespecified multi-compartment safety margin). Human translation: not established (recorded separately, not by downgrading the preclinical grade). Telomere axis (optional): UNTESTED. Verdict: does not pass (healthy-state partial).

**Grade justification, vaccine route:** Persistent senescence: MET. Immune accessibility: MET. Functional recognition: UNTESTED (no vaccine assay; a CAR result is nomination only). Healthy-state exclusion: UNTESTED (no vaccine-route safety margin). Human translation: not established. Telomere axis (optional): UNTESTED. Verdict: does not pass, because two of the four compulsory criteria (functional recognition and healthy-state exclusion) are untested; the telomere axis is optional and is not counted among the four.

## 9.2 GPNMB, native-surface-antigen vaccine route

**Study identifiers and status:** Suda et al. (2021) Nature Aging 1:1117-1126, https://doi.org/10.1038/s43587-021-00151-2; Suda et al. (2022) follow-up. Peer-reviewed. Related target-safety context: Yoshida et al. (2020) Nature Communications 11:2482 (CD153).

**Intended human product and route:** A native-surface-antigen vaccine that raises antibodies against the GPNMB extracellular domain; graded against the antibody-mediated native-surface route, not against HLA-peptide requirements.

**Model and population:** GPNMB identified from senescent-cell transcriptomes filtered to genes with predicted transmembrane domains, then confirmed increased on senescent vascular endothelial cells and in atherosclerotic

patient samples; a Gpnmb-promoter luciferase reporter mouse was generated. Vaccine studies used high-fat-diet mice, apolipoprotein-E-knockout atherosclerosis mice, middle-aged wild-type mice, and a Hutchinson-Gilford-progeria-like accelerated-aging model.

**Comparator and target-dependence controls:** Peptide vaccine from the GPNMB extracellular domain versus control vaccine; genetic ablation of Gpnmb-positive cells as an orthogonal target-defining control; efficacy also benchmarked against dasatinib-plus-quercetin and navitoclax.

**Senescence characterization (persistence evidence):** GPNMB was enriched in cells identified as senescent from senescence transcriptomes and increased in atherosclerotic patient tissue, and genetic ablation of GPNMB-positive cells attenuated adipose-tissue senescence. This is population- and tissue-level evidence: same-cell in-vivo co-localization of GPNMB with two or more orthogonal senescence markers (for example SA-beta-galactosidase plus p16 or p21 in the individual targeted cells, at a defined time point) is not documented in the source, and genetic ablation establishes pathogenicity rather than the senescent phenotype of the targeted cells. The persistent-senescence criterion is therefore graded partial, not met.

**Key reported effects and sample sizes:** High-fat-diet mice vaccinated 4 weeks into a 12-week diet showed decreased GPNMB, reduced senescent-cell burden, and improved metabolic parameters; atherosclerotic plaque burden (aortic plaque area) was reduced in apolipoprotein-E-knockout mice on a high-fat diet, each comparison being GPNMB vaccine versus control vaccine; median lifespan in the Hutchinson-Gilford-progeria-like model increased from about 21 to 25 weeks (about 20 percent, males). These are the selected quantitative values available from the report; the exact per-group animal counts for the metabolic, aortic-plaque, and survival cohorts are given in the corresponding figures of Suda et al. (2021) (the atherosclerosis-characteristics and progeroid-survival panels) and are not reproduced in full here, so this log provides selected values while the complete numerical data remain in the primary report.

**Safety and healthy-state findings:** GPNMB is expressed on macrophage-lineage, bone-associated, and neural cells; no prespecified normal-tissue safety margin was demonstrated in any required healthy compartment.

**Contradictory or limiting evidence:** In young mice GPNMB has been reported to play a protective metabolic role, with knockout associated with impaired metabolism and macrophage-driven inflammation, indicating context-dependent normal function; whether all GPNMB-high cells are senescent is unresolved. Human immunogenicity and antibody-dependent clearance are not established.

**Grade justification:** Persistent senescence: PARTIAL (population- and tissue-level enrichment in senescent-defined cells and patient tissue, but same-cell multi-marker co-localization not documented; ablation shows pathogenicity, not the senescent phenotype of the targeted cells). Immune accessibility: MET (direct surface detection of GPNMB on intact senescent cells, the basis for antibody targeting, supported by the reporter and surface staining). Functional recognition: MET on the preclinical grading unit (mouse vaccination raised antibodies and reduced GPNMB-positive cells versus a control vaccine through an antibody-dependent, natural-killer-mediated mechanism, which is controlled antigen-dependent clearance in the intended native-surface route). Human translation: not established (human immunogenicity and antibody-dependent clearance untested; recorded separately, not by downgrading the preclinical grade). Healthy-state exclusion: UNTESTED (no prespecified safety margin demonstrated in any required compartment). Telomere axis (optional): UNTESTED. Verdict: does not pass, because persistent senescence is partial and healthy-state exclusion is untested.

### 9.3 GPNMB cellular-route variants (distinct from the Suda antibody route)

**Cao et al., 2024 (dendritic-cell vaccine):** Contribution: a distinct GPNMB product that engages cellular immunity rather than antibody/NK-ADCC. Model and design: high-fat-diet young and aged mice and a natural-aging model; a cationic-protein-fused GPNMB peptide (Gpnmb-CP) pulsed into dendritic cells (Gpnmb-CP-DC), compared with the Gpnmb-CP protein vaccine. Effector mechanism: enhanced dendritic-cell maturation and activation of CD8-positive T cells with cytotoxic and memory responses. Principal effect: selective elimination of senescent adipocytes and improved metabolic abnormalities, including in aged mice where the protein vaccine

was ineffective. Safety evidence: no prespecified healthy-tissue safety margin reported. Per-group sample sizes are in the source figures. Grade summary (cellular vaccine route, preclinical grading): accessibility met; functional recognition met on controlled mouse CD8 evidence, with human translation not established (recorded separately, not by downgrading the preclinical grade); healthy-state untested; telomere untested; does not pass. (Cao et al., 2024, Biomedicine & Pharmacotherapy 179, 117433, https://doi.org/10.1016/j.biopha.2024.117433)

**Zhang et al., 2025 (nanovaccine GK-NaV):** Contribution: a second cellular-route GPNMB product; should not be merged with the antibody-route Suda vaccine. Model and design: high-fat-diet-induced progeroid mice; a self-assembled nanovaccine (GK-NaV) fusing cationic protein K36 with the Gpnmb seno-antigen peptide. Effector mechanism: efficient dendritic-cell uptake, DC maturation (CD80/CD86 upregulation), and activation of CD8-positive T cells with immunological memory; an antigen-depot effect after subcutaneous immunization. Principal effect: selective elimination of GPNMB-positive senescent adipocytes and cardiomyocytes and improvement of adipose metabolic disorder and cardiac dysfunction. Safety evidence: proposed higher selectivity and fewer off-target effects, but no prespecified healthy-tissue safety margin reported. Per-group sample sizes are in the source figures. Grade summary (cellular vaccine route, preclinical grading): accessibility met; functional recognition met on controlled mouse CD8 evidence, with human translation not established (recorded separately); healthy-state untested; telomere untested; does not pass. (Zhang et al., 2025, Hum Vaccin Immunother 21:2479229, https://doi.org/10.1080/21645515.2025.2479229)

## 9.4 Additional appraised candidates (summary)

| Candidate (route) | Model / population | Comparator / control | Key reported effect | Principal limiting evidence | Grade summary |
|---|---|---|---|---|---|
| Frescas et al. 2017 (Table 3) | Human fibroblasts; oxidative surface epitope (oxidized vimentin) | Antibody-specificity controls; no in-vivo model | Discovery of a senescence-associated oxidized-vimentin surface epitope and specific antibodies | No senolysis or safety data; discovery only | Contributes to accessibility/antigen discovery only; no functional-recognition or healthy-state grade |
| CD153 (peptide vaccine) | High-fat-diet mice; adipose senescent T cells | Complement-dependent cytotoxicity; control vaccine | Reduced senescent T cells; improved glucose metabolism | CD153 also on non-senescent activated T cells | Selective within niche; narrow coverage; telomere untested |
| CD38 (peptide vaccine) | Aged mice; myeloid immunometabolic cells | Control vaccine; cellular response readout | Immune response and reduction of target cells reported | CD38 has broad essential immunometabolic roles | Healthy-state exclusion under-tested; telomere untested |
| Yang et al. 2023 (NKG2D; CAR-T) | Mouse; stress-ligand+ cells | Ligand-dependent killing | Killing of ligand-positive senescent cells | General stress ligands on activated healthy cells | Selectivity unresolved; telomere untested |
| Deng et al. 2024 (NKG2D; CAR-T) | Mouse and non-human primate; stress-ligand+ cells | Ligand-dependent killing; primate tolerability | Cross-species killing of ligand-positive cells | General stress ligands on activated healthy cells | Selectivity unresolved; telomere untested |
| Majewska et al. 2024 (PD-L1/PD-L2; checkpoint) | Mouse therapy-induced senescence | Mechanism-linked; reversible modality | Checkpoint-mediated clearance reported | Checkpoints inducible and not senescence-exclusive | Reversible; not senescence-specific; telomere untested |
| Iltis et al. 2025 (GD3; antibody) | Mouse therapy-induced senescence | Mechanism-linked; NK-linked | NK-mediated clearance reported | GD3 absent from several oncogene-induced senescent states | Different target from Majewska; telomere untested |
| Senescent-cell lysate (DC vaccine) | Mouse tumor models | Polyclonal response; no defined target | Antitumor activity | Mediating antigens undefined | Discovery-stage; not a defined-target test |

Sources for this log: Amor et al. (2020, 2024); Eskiocak et al. (2026); Frescas et al. (2017); Suda et al. (2021, 2022); Cao et al. (2024); Zhang et al. (2025); Yoshida et al. (2020); Yu et al. (2025); Yang et al. (2023); Deng et al. (2024); Majewska et al. (2024); Iltis et al. (2025); Ichim et al. (2025). Together these cover every study appraised in Tables 3 and 10; full bibliographic details appear in the main article reference list.
